# A mammalian *Wnt5a–Ror2–Vangl2* axis controls the cytoskeleton and confers cellular properties required for alveologenesis

**DOI:** 10.7554/eLife.53688

**Published:** 2020-05-12

**Authors:** Kuan Zhang, Erica Yao, Chuwen Lin, Yu-Ting Chou, Julia Wong, Jianying Li, Paul J Wolters, Pao-Tien Chuang

**Affiliations:** 1 Cardiovascular Research Institute, University of California, San FranciscoSan FranciscoUnited States; University of PennsylvaniaUnited States; University of PennsylvaniaUnited States

**Keywords:** lung, alveolus, planar cell polarity, cytoskeleton, myofibroblast, alveolar epithelial cell, Mouse

## Abstract

Alveolar formation increases the surface area for gas-exchange and is key to the physiological function of the lung. Alveolar epithelial cells, myofibroblasts and endothelial cells undergo coordinated morphogenesis to generate epithelial folds (secondary septa) to form alveoli. A mechanistic understanding of alveologenesis remains incomplete. We found that the planar cell polarity (PCP) pathway is required in alveolar epithelial cells and myofibroblasts for alveologenesis in mammals. Our studies uncovered a *Wnt5a–Ror2–Vangl2* cascade that endows cellular properties and novel mechanisms of alveologenesis. This includes PDGF secretion from alveolar type I and type II cells, cell shape changes of type I cells and migration of myofibroblasts. All these cellular properties are conferred by changes in the cytoskeleton and represent a new facet of PCP function. These results extend our current model of PCP signaling from polarizing a field of epithelial cells to conferring new properties at subcellular levels to regulate collective cell behavior.

## eLife digest

The lungs enable the exchange of gases between inhaled air and the bloodstream. This exchange happens in structures called alveoli, which have a large surface area that aids in efficient gas exchange. Shortly after birth in mice, or during the last few months before birth in humans, alveoli develop folds called secondary septa that increase their surface area and improve the efficiency of gas exchange.

Several types of cells work together to form secondary septa. Surface cells called epithelia and underlying “myofibroblast” cells and small blood vessels must both communicate and move together to build the septa. The processes that control the formation of septa have not been fully studied. In other cases, a cell signaling pathway known as the planar cell polarity (PCP) pathway has been shown to help coordinate cell movements. The PCP pathway works by changing the cytoskeleton of cells, which is the series of protein fibers that give cells their shape and structure and the ability to move.

Zhang et al. have now studied septa in mouse lungs and revealed how three genes – *Wnt5a*, *Ror2* and *Vangl2* – in the PCP pathway control this process. This pathway oversees changes to the cytoskeleton in both epithelial cells and myofibroblasts, helping the cells to change shape and move together to form septa. Unusually, the PCP pathway has different effects in different cells, rather than affecting all cells similarly. This is partly due to so-called PDGF signals from the epithelial cells that help to guide the growth and movement of myofibroblasts. This process is helped by the epithelial cells changing their shape to accommodate myofibroblasts during septa formation.

Further analysis also showed reduced PCP signaling in patients with chronic obstructive pulmonary disease, also known as COPD. This could be a factor in the extensive lung damage seen in these patients. These findings help to explain a key lung development process and may provide new insights to understand lung diseases such as COPD.

## Introduction

Gas exchange, the essential function of the lung, depends on the production of a sufficient number of functional alveoli to provide surface area for gas exchange (Burri, 2006; Whitsett and Weaver, 2015; Chao et al., 2016). Elucidating the molecular mechanisms by which alveoli are formed remains a major unresolved question. Lung branching morphogenesis is followed by the construction of primary saccules at the distal end of the branching lung tree. The smooth wall of the primary saccules is further modified by the generation of secondary crests or septa, which divide the saccules into alveoli. As a result, the surface area of gas exchange is greatly increased to meet the high demand of oxygen consumption in terrestrial, warm-blooded animals. Uncovering the molecular basis of alveolar development will also provide insight into diseases that affect the alveoli. For instance, bronchopulmonary dysplasia (BPD), in which maturation of alveoli fails to occur (Silva et al., 2015), is prevalent in premature babies. Moreover, insults to the lung in adult life such as infectious diseases or chronic obstructive pulmonary disease (COPD) can lead to destruction of alveoli and respiratory failure (Patel et al., 2019). A mechanistic understanding of alveolar formation will offer new therapies to regenerate alveolar surface area and treat diseases caused by loss of alveoli (Rodríguez-Castillo et al., 2018).

The most important step in alveolar development is the formation of epithelial folds (secondary septa) within the saccules, in which thin and flat alveolar type I (AT1) cells cover a core of myofibroblasts, connective tissue and capillaries (Branchfield et al., 2016). Compared to AT1 cells, alveolar type II (AT2) cells contribute to a much smaller surface area for gas exchange but they play a central role in lung expansion after birth by secreting pulmonary surfactants. During the first 2–3 days of postnatal life, the smooth wall (the primary septa) of saccules in wild-type mouse lungs is modified by epithelial folding, which is termed rudimentary secondary crests or septa, to increase the surface area for gas exchange. Secondary septa consist of alveolar type I cells that cover a core of myofibroblasts, connective tissue and capillaries (Chao et al., 2016). Elongation of secondary septa is associated with deposition of elastin by myofibroblasts and maturation of capillaries. At around postnatal day 5, many of the primary saccules have been subdivided into alveoli by secondary septation although this process will continue for another 25 days and beyond. Secondary septa formation greatly increases the surface area for gas exchange in mammals and is the most critical event during alveolar formation.

The current model posits that interstitial fibroblasts respond to platelet-derived growth factor (PDGF) signaling and migrate to the prospective secondary septa to form alveolar myofibroblasts (Chao et al., 2016), which express smooth muscle actin (SMA). It is postulated that myofibroblast migration and elastin deposition by myofibroblasts provide the driving force for secondary septa elongation. However, the molecular mechanisms that control cell migration remain poorly understood. Similarly, the molecular processes that mediate interactions between alveolar epithelial cells and prospective myofibroblasts during secondary septa formation are unknown. Importantly, whether alveolar epithelial cells also play an active role in promoting alveologenesis has not been determined. New insight into these key issues is pivotal to our mechanistic understanding of alveolar formation.

We reasoned that signaling pathways that control the actomyosin cytoskeleton likely play a crucial role in cell migration and interaction during secondary septa formation. The planar cell polarity (PCP; also known as tissue polarity) pathway (Torban et al., 2012; Campanale et al., 2017; Davey and Moens, 2017; Butler and Wallingford, 2017; Gray et al., 2011; Humphries and Mlodzik, 2018; Sokol, 2015; Zallen, 2007; Blair and McNeill, 2018; Peng and Axelrod, 2012) has been shown to regulate the actomyosin cytoskeleton. In this study, we employed PCP signaling as a tool to reveal the molecular basis of secondary septation and alveolar formation in mice. PCP (a non-canonical Wnt pathway) is a fundamental, conserved mechanism for polarizing a field of cells within the plane of an epithelial cell sheet and is essential in many tissues. PCP signaling is initiated by the binding of Wnt ligands to the Frizzled (Fz) receptors (Wang et al., 2016). The signal is relayed by a set of core PCP components that include cytoplasmic and membrane proteins. The outcome of PCP signaling is an altered actomyosin cytoskeleton that is local and is associated with polarized cellular function in a field of cells. The role of PCP components in lung development has been described (Yates et al., 2010; Poobalasingam et al., 2017), but its molecular mechanisms are unknown. Other well-characterized systems that require PCP include convergent extension in neural tube closure, hair cell orientation in the cochlea of the inner ear, hair follicle orientation, and motile cilia positioning in the trachea. It is somewhat surprising that, in each system, the molecular mechanisms that mediate PCP’s effects on cellular behavior are largely underexplored.

Our work described in this report has discovered a new mechanism of PCP signaling in which a *Wnt5a–Ror2–Vangl2* axis controls tissue patterning through regulating cellular properties and not tissue polarity. Specifically, PCP signaling controls PDGF ligand secretion from alveolar type I and type II cells, instructs cell shape change of alveolar type I cells, and regulates myofibroblast migration. All of these are mediated though the actomyosin cytoskeleton. These novel insights significantly extend our current understanding of alveolar formation and PCP signaling in tissue patterning. They not only provide a new conceptual framework for understanding how the alveolus is generated during development but also have a major impact on advancing disease mechanisms caused by alveolar malformation or destruction.

## Results

### Inactivation of *Vangl2* in the distal lung epithelium disrupts alveolar formation

To understand how PCP pathway components control alveolar formation, we eliminated PCP signaling in the distal lung epithelium by generating *Vangl2^f/f^; Sox9^Cre/+^* mice. A floxed (f) allele of *Vangl2* (*Vangl2^f^*) (Song et al., 2010) was converted into a null allele through strong expression of Cre from the *Sox9* locus (*Sox9-Cre*) (Akiyama et al., 2005) in the SOX9^+^ distal lung epithelium. During canalicular (16.5–17.5 *days post coitus* (*dpc*)) and saccular development (17.5 *dpc*–postnatal (P) day 4), following the completion of branching morphogenesis, airspace at the terminal bronchioles is subdivided to form saccules (Schittny, 2017). Lined by alveolar type I and type II cells, saccules function as the primitive gas-exchange unit. As expected, VANGL2 protein was barely detectable in the distal epithelium of *Vangl2^f/f^; Sox9^Cre/+^* lungs by 18.5 *dpc* (shortly before birth), while VANGL2 expression in the lung mesenchyme and elsewhere was unaffected (Figure 1—figure supplements 1, 2 and 3). Loss of *Vangl2* abolishes PCP signaling since *Vangl2* encodes a four-pass transmembrane protein and is an essential component of mammalian PCP signaling (Bailly et al., 2018).

**Figure 1.**
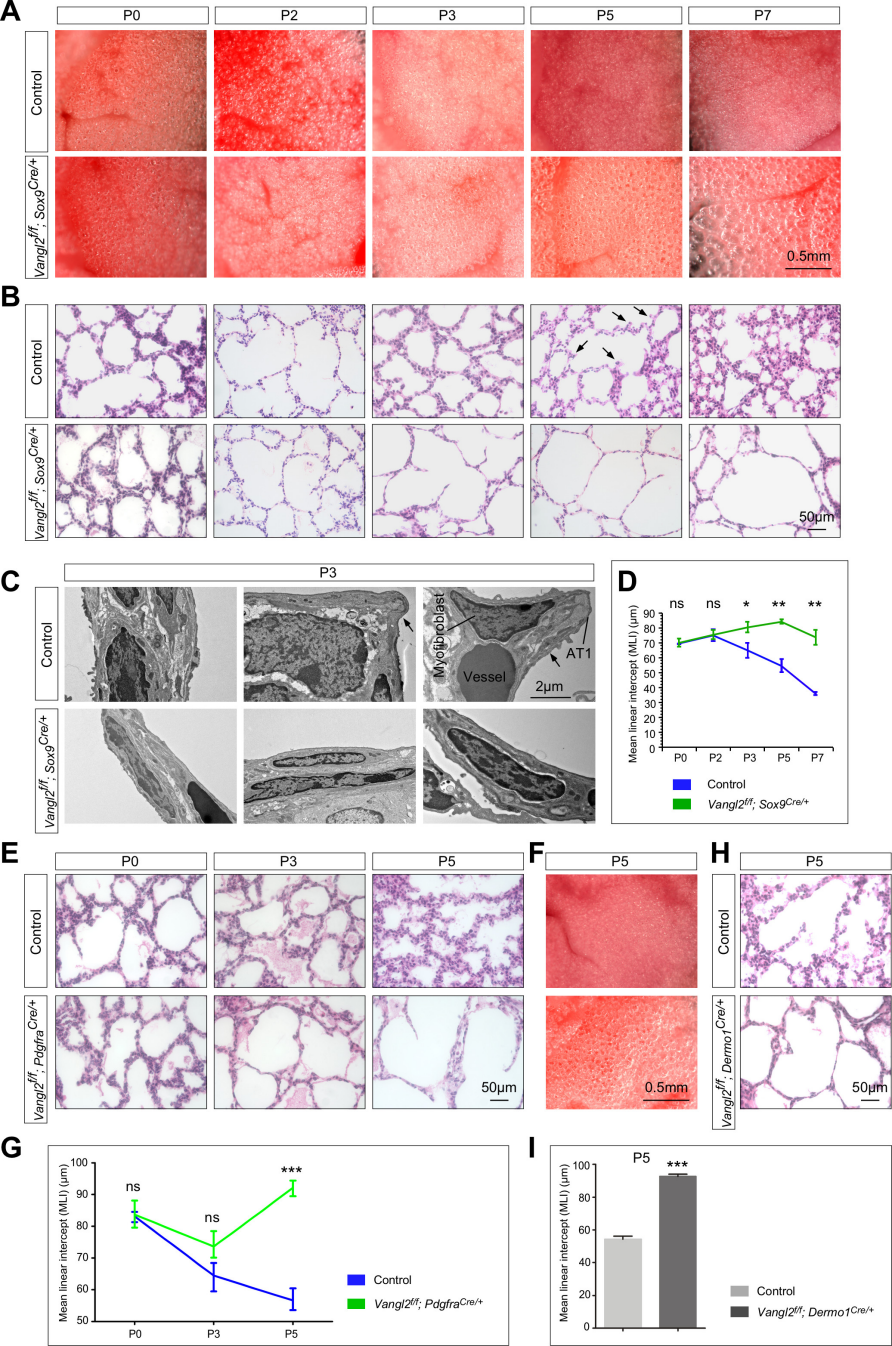
*Vangl2* is required in both the lung epithelium and mesenchyme for alveolar formation. (**A**) Surface view of dissected lungs from wild-type and *Vangl2^f/f^; Sox9^Cre/+^* mice at different postnatal (P) stages as indicated. Enlarged saccules were discerned in *Vangl2^f/f^; Sox9^Cre/+^* lungs at P3 and their size increased significantly as postnatal lung development proceeded. (**B**) Hematoxylin and eosin-stained lung sections of wild-type and *Vangl2^f/f^; Sox9^Cre/+^* mice at different postnatal stages. Histological analysis confirmed the presence of enlarged saccules in *Vangl2*-deficient lungs starting at P3. Arrows point to rudimentary secondary septa. (**C**) Transmission electron micrographs of lungs from wild-type and *Vangl2^f/f^; Sox9^Cre/+^* mice at P3. Rudimentary secondary septa (arrows), in which the alveolar type I (AT1) cells encased myofibroblasts and blood vessels, were seen in control but not *Vangl2*-deficient lungs. (**D**) Measurement of the mean linear intercept (MLI) in wild-type and *Vangl2^f/f^; Sox9^Cre/+^* lungs (n = 3 for each group). The MLI was increased in *Vangl2*-deficient lungs, starting at P3. (**E**) Hematoxylin and eosin-stained lung sections of wild-type and *Vangl2^f/f^; Pdgfra^Cre/+^* mice at different postnatal stages. Enlarged saccules were detected in *Vangl2^f/f^; Pdgfra^Cre/+^* lungs at P3 and their size increased significantly as postnatal lung development proceeded. (**F**) Surface view of dissected lungs from wild-type and *Vangl2^f/f^; Pdgfra^Cre/+^* mice at P5. Larger saccules were found in *Vangl2* mutant lungs induced by *Pdgfra^Cre^*. (**G**) Measurement of the MLI in wild-type and *Vangl2^f/f^; Pdgfra^Cre/+^* lungs (n = 3 for each group). The MLI was increased in *Vangl2*-deficient lungs, starting at P5. (**H**) Hematoxylin and eosin-stained lung sections of wild-type and *Vangl2^f/f^; Dermo1^Cre/+^* mice at P5. Larger saccules were found in *Vangl2* mutant lungs induced by *Dermo1^Cre^*. (**I**) Measurement of the MLI in wild-type and *Vangl2^f/f^; Dermo1^Cre/+^* lungs (n = 3 for each group). The MLI was increased in *Vangl2*-deficient lungs. All values are mean ± SEM. (*) p<0.05; (**) p<0.01; ns, not significant (unpaired Student’s *t*-test).

*Vangl2^f/f^; Sox9^Cre/+^* animals were born alive and cannot be distinguished from their wild-type littermates by their outer appearance or activity at birth (Figure 1—figure supplement 3). However, at postnatal day 2, *Vangl2^f/f^; Sox9^Cre/+^* mice can be identified by their slightly diminished body size. The reduced size and activity in these mutants became more pronounced as postnatal development proceeded. Mortality was observed in *Vangl2^f/f^; Sox9^Cre/+^* mice at various time points postnatally, especially between postnatal day 3 and 7 (mostly after day 4/5). Interestingly, a small number of *Vangl2^f/f^; Sox9^Cre/+^* mice survived beyond postnatal day 7. *Vangl2*-deficient lungs consisted of enlarged saccules (Figure 1A) and failed to generate alveoli (Figure 1B). These results establish an essential role of *Vangl2* in alveolar formation (traditionally defined as P5-25 in mice) during lung development.

**Figure 1—figure supplement 1.**
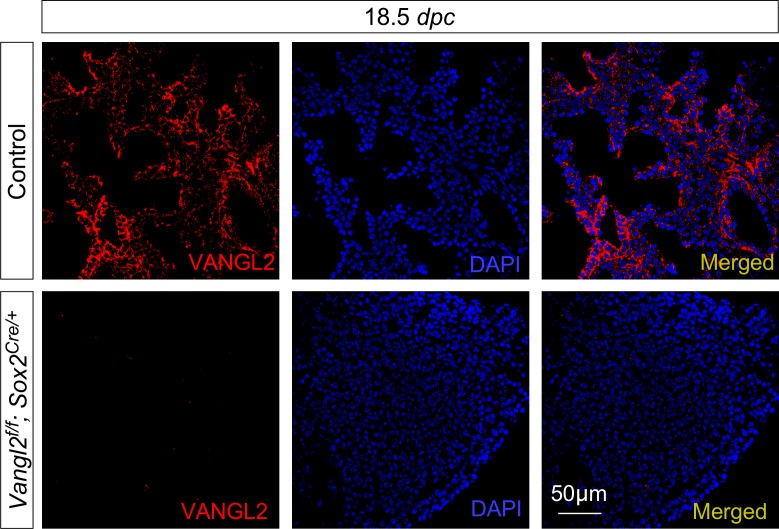
VANGL2 is broadly expressed in both the lung epithelium and mesenchyme. Immunostaining of lung sections collected from control and *Vangl2^f/f^; Sox2^Cre/+^* mice at 18.5 *days post coitus* (*dpc*). VANGL2 was detected in both the lung epithelium and mesenchyme in wild-type lungs. VANGL2 immunoreactivity was completely absent in the lungs of *Vangl2^f/f^; Sox2^Cre/+^* mice, validating the VANGL2 immunoreactivity detected by VANGL2 antibodies. Note that early expression of *Sox2-Cre* in all epiblast cells by 6.5 *dpc* effectively converted *Vangl2^f^* into a null allele in all embryonic lineages. *Vangl2^f/f^; Sox2^Cre/+^* is in essence equivalent to *Vangl2^–/–^*.

**Figure 1—figure supplement 2.**
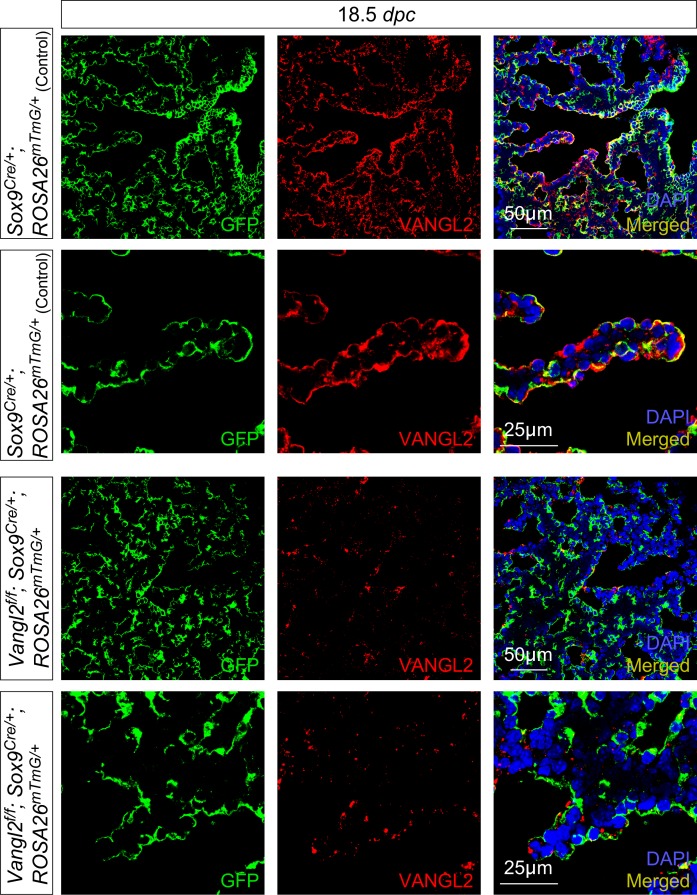
VANGL2 is selectively removed in the distal lung epithelium by *Sox9-Cre*. Immunostaining of lung sections collected from *Sox9^Cre/+^; ROSA26^mTmG/+^* (control) and *Vangl2^f/f^; Sox9^Cre/+^; ROSA26^mTmG/+^* mice at 18.5 *days post coitus* (*dpc*). *Sox9-Cre* induced GFP expression from the *ROSA26^mTmG^* locus. VANGL2 immunoreactivity was selectively lost in the lung epithelium (GFP^+^) of *Vangl2^f/f^; Sox9^Cre/+^; ROSA26^mTmG/+^* mice; VANGL2 expression was retained in the lung mesenchyme.

**Figure 1—figure supplement 3.**
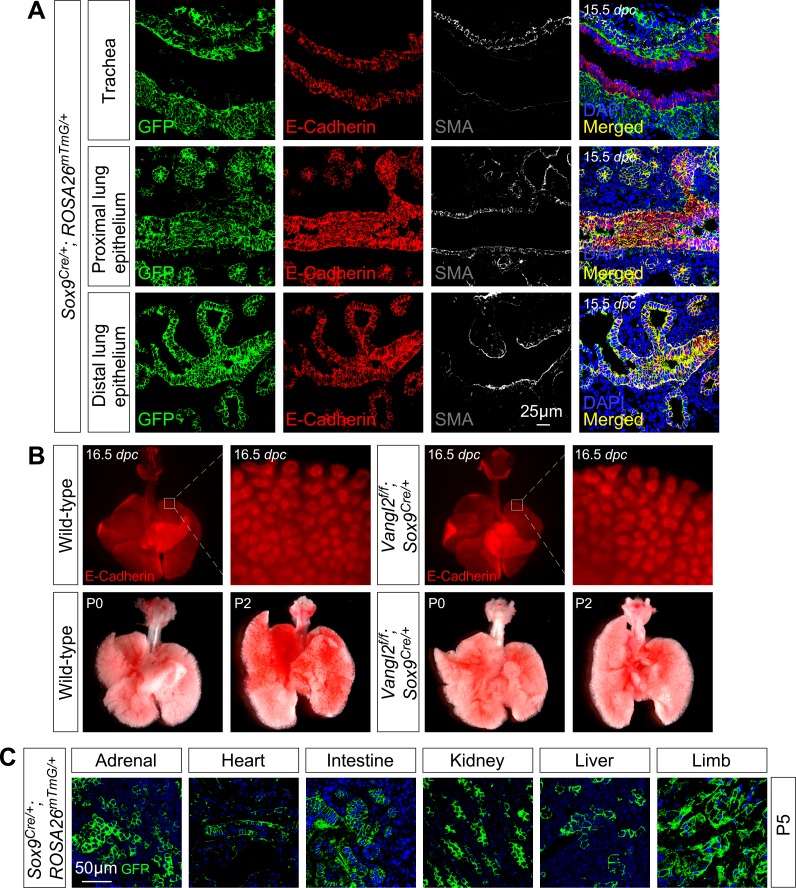
*Sox9-Cre* is broadly expressed but *Vangl2^f/f^; Sox9^Cre/+^* mice do not display defects in lung branching or saccule formation. (**A**) Immunostaining of lung sections collected from *Sox9^Cre/+^; ROSA26^mTmG/+^* mice at 15.5 *days post coitus* (*dpc*). (**B**) Ventral views of dissected lungs from control or *Vangl2^f/f^; Sox9^Cre/+^* mice at 16.5 *dpc*, postnatal (**P**) day 0 or 2. No apparent phenotypes in branching (likely due to the presence of *Vangl1*) or sacculation were discerned in the mutant lungs. (**C**) Immunostaining of sections of various tissues collected from *Sox9^Cre/+^; ROSA26^mTmG/+^* mice at P5.

**Figure 1—figure supplement 4.**
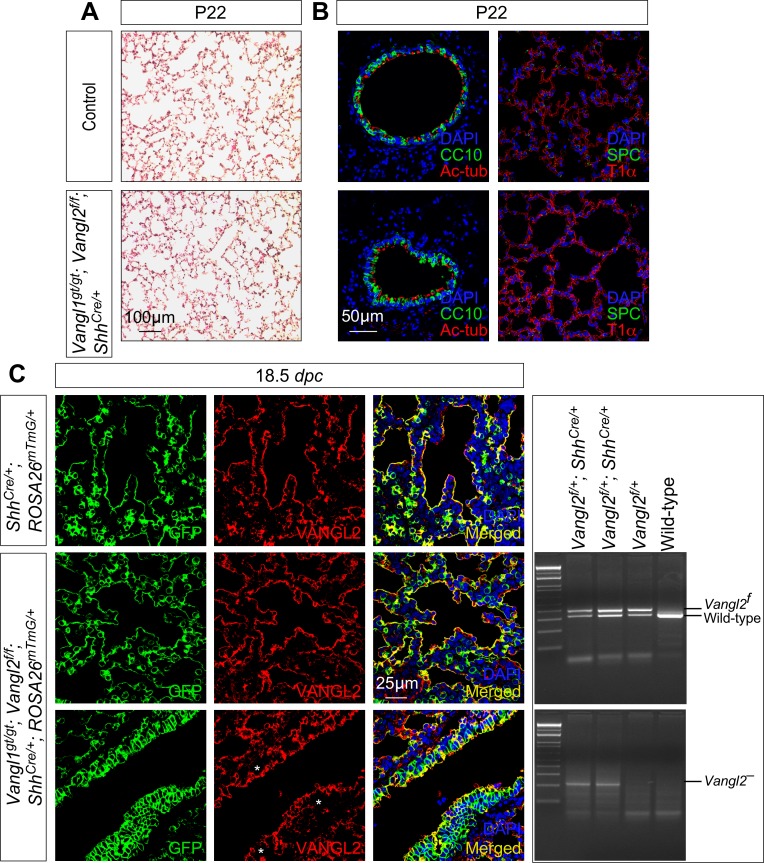
*Shh-Cre* fails to efficiently remove *Vangl2* in the lung epithelium. (**A**) Hematoxylin and eosin-stained lung sections of control and *Vangl1^gt/gt^; Vangl2^f/f^; Shh^Cre/+^* mice at postnatal (P) day 22. Alveoli formed properly in *Vangl1^gt/gt^; Vangl2^f/f^; Shh^Cre/+^* lungs, which were indistinguishable from control lungs. (**B**) Immunostaining of lung sections collected from control and *Vangl1^gt/gt^; Vangl2^f/f^; Shh^Cre/+^* mice at P22. There was no discernable difference in cell type specification and distribution between control and *Vangl1^gt/gt^; Vangl2^f/f^; Shh^Cre/+^* lungs. CC10 marked club (Clara) cells and Ac-tub marked ciliated cells in the airways. SPC labeled alveolar type II cells and T1α labeled alveolar type I cells in alveoli. (**C**) Immunostaining of lung sections collected from *Shh^Cre/+^; ROSA26^mTmG/+^* (control) and *Vangl1^gt/gt^; Vangl2^f/f^; Shh^Cre/+^; ROSA26^mTmG/+^* mice at 18.5 *days post coitus* (*dpc*). *Shh-Cre* induced epithelial GFP expression from the *ROSA26^mTmG^* locus. Only small pockets (*) of the proximal lung epithelium in *Vangl1^gt/gt^; Vangl2^f/f^; Shh^Cre/+^; ROSA26^mTmG/+^* mice displayed VANGL2 loss in comparison with controls. This is consistent with the presence of *Vangl2^–^* (null) by PCR.

**Figure 1—figure supplement 5.**
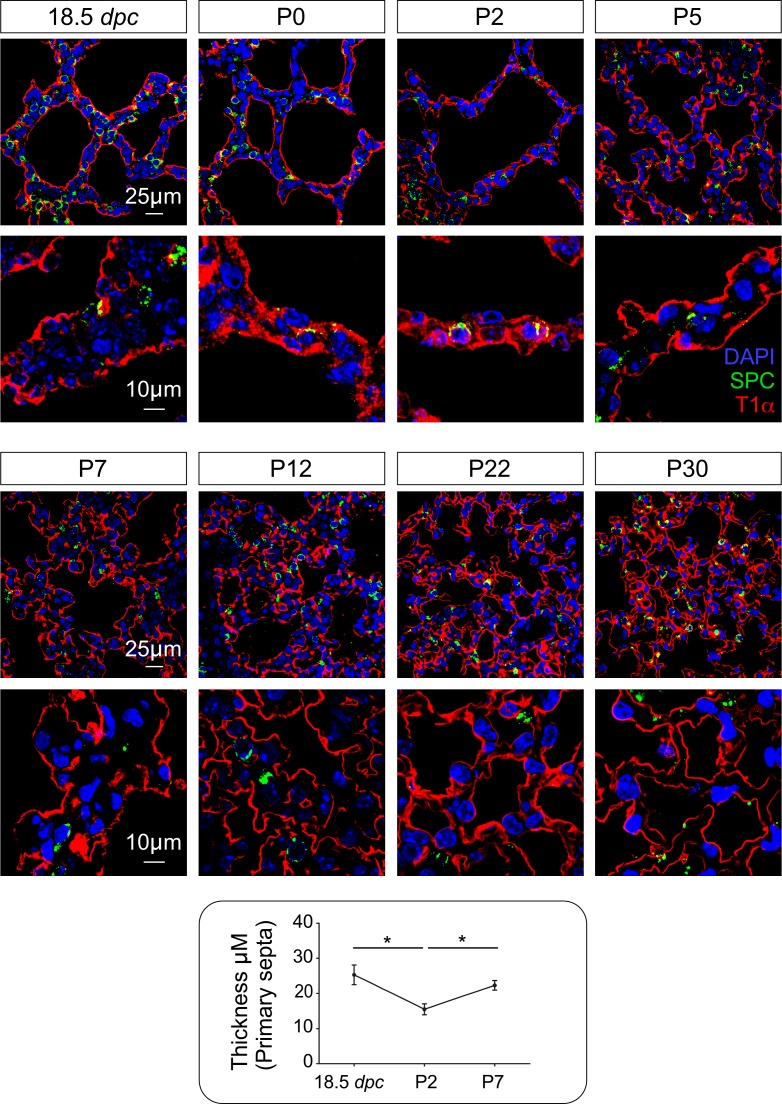
A time course of the development of the alveolar septa. Immunostaining of lung sections collected from wild-type mice at 18.5 *days post coitus* (*dpc*) and various postnatal (P) days as indicated. The primary septa appeared to be visibly thinner during the first three days of postnatal life but its thickness increased subsequently. T1α labeled alveolar type I cells while SPC marked alveolar type II cells. All values are mean SEM. (*) p<0.05 (unpaired Student’s *t*-test).

**Figure 1—figure supplement 6.**
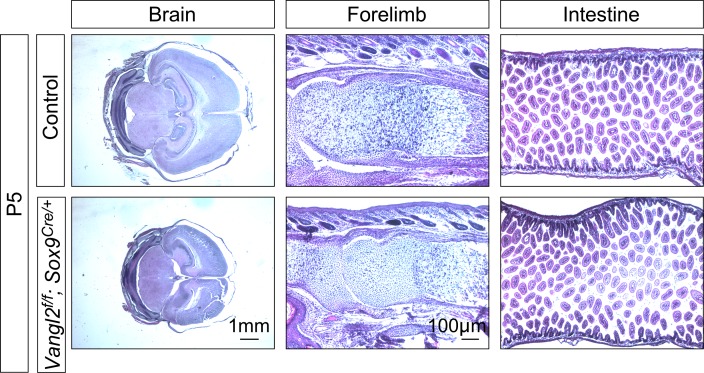
Histological analysis of tissues and organs in *Vangl2^f/f^; Sox9^Cre/+^* mice. Hematoxylin and eosin-stained tissue sections of control and *Vangl2^f/f^; Sox9^Cre/+^* mice at postnatal (P) day 5. Histological analysis revealed no apparent defects in the brain, bone and small intestine where *Sox9-Cre* is expressed.

**Figure 1—figure supplement 7.**
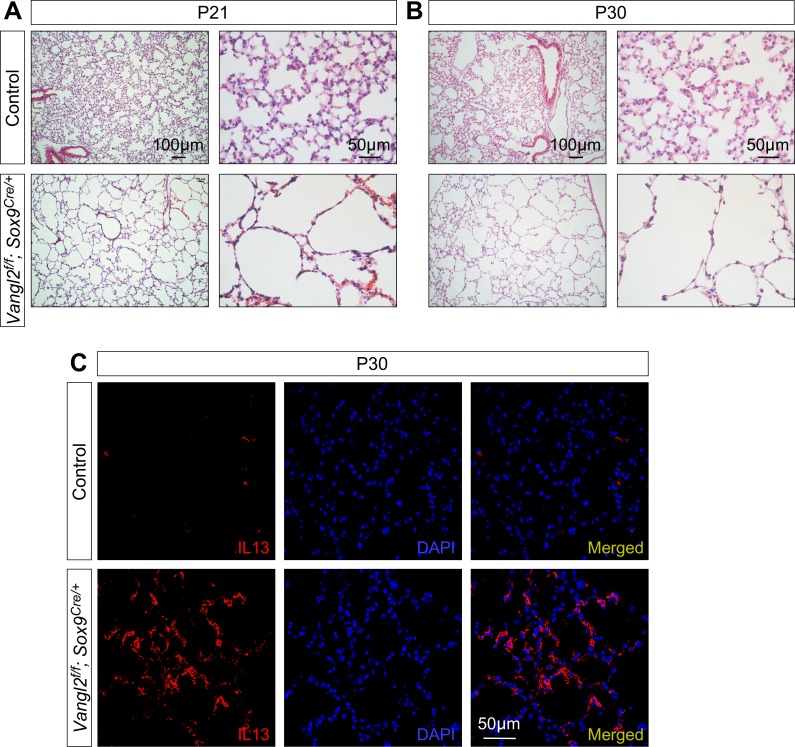
Loss of epithelial *Vangl2* disrupts secondary septa and alveolar formation. (**A, B**) Hematoxylin and eosin-stained lung sections of control and *Vangl2^f/f^; Sox9^Cre/+^* mice at postnatal (P) day 21 and 30. Secondary septa and alveoli failed to form and only thin primary septa persisted in *Vangl2^f/f^; Sox9^Cre/+^* lungs. (**C**) Immunostaining of lung sections collected from control and *Vangl2^f/f^; Sox9^Cre/+^* mice at P30. IL13 expression levels were elevated in the mutant lungs. Note that these *Vangl2^f/f^; Sox9^Cre/+^* animals were survivors since many of them succumbed to death prior to P10. No survivors beyond four weeks carry the genotype of *Vangl1^gt/+^; Vangl2^f/f^; Sox9^Cre/+^*, consistent with a minor role of *Vangl1* in alveologenesis.

Of note, the mammalian genome contains two homologs (*Vangl1* and *Vangl2*) of fly *Van Gogh (Van)/strabismus. Vangl1*-deficient mice that are homozygous for a gene-trapped null allele of *Vangl1* (*Vangl1^gt^*) (Torban et al., 2008) are fully viable without apparent lung phenotypes and reproduce with normal litter size. This suggests that *Vangl1* plays a minor role in alveolar formation and loss of *Vangl1* is compensated by *Vangl2* in alveologenesis. Of note, we failed to recover live *Vangl1^gt/gt^; Vangl2^f/f^; Sox9^Cre/+^* mice postnatally. Hence, our study on alveologenesis has focused on *Vangl2*. Surprisingly, the widely used *Shh^Cre^* (Harfe et al., 2004), which drives Cre expression along the entire developing lung epithelium, failed to effectively eliminate VANGL2 (Figure 1—figure supplement 4). *Vangl1^gt/gt^; Vangl2^f/f^; Shh^Cre/+^* mice were fully viable and fertile without apparent phenotypes (Figure 1—figure supplement 4). In the literature, a hypomorphic (reduced function) allele of *Vangl2*, *Vangl2^Lp^* (*loop tail*) (Murdoch et al., 2001), has been reported in several studies. Homozygous *Vangl2^Lp/Lp^* mice die in utero due to neural tube defects while heterozygous *Vangl2^Lp/+^* mice display mild alveolar defects in adults but minimal alveolar phenotypes at postnatal day 7 (Poobalasingam et al., 2017). Thus, *Vangl2^Lp/+^* mice are unsuitable for studying the molecular mechanisms of alveolar formation. This would require complete removal of *Vangl2* function in a select compartment (*e.g.*, the lung epithelium, mesenchyme or others) through conditional inactivation of *Vangl2^f^* using various mouse Cre lines. Our approach thus enables us to reveal how PCP signaling regulates alveolar development.

To determine the cause of neonatal lethality in *Vangl2^f/f^; Sox9^Cre/+^* mice, we examined lungs from control and *Vangl2^f/f^; Sox9^Cre/+^* mice at different time points postnatally. We noticed that the primary septa in control lungs became visibly thinner during the first three days of postnatal life likely due to flattening of AT1 cells (Figure 1—figure supplement 5). The thickness of the primary septa subsequently increased, presumably reflecting cell proliferation and structural changes in the mesenchyme and folding of AT1 cells (Figure 1—figure supplement 5), which is correlated with the progression of alveolar development (Yang et al., 2016). No obvious difference in the gross morphology of the lungs was noticed between control and *Vangl2^f/f^; Sox9^Cre/+^* mice prior to postnatal day 3 (Figure 1A). Histological analysis of control and *Vangl2^f/f^; Sox9^Cre/+^* lungs showed no apparent difference prior to postnatal day 3 (Figure 1B). Loss of *Vangl2* mediated by *Sox9-Cre* thus does not disrupt branching morphogenesis or saccule formation (Figure 1—figure supplement 3).

At postnatal day 3, enlarged saccules were discerned in *Vangl2^f/f^; Sox9^Cre/+^* lungs in comparison with controls under a dissecting microscope (Figure 1A). This finding was confirmed by morphological analysis of dissected lungs using light microscopy (Figure 1B). Transmission electron microscopy (TEM) of control and *Vangl2^f/f^; Sox9^Cre/+^* lungs revealed lack of rudimentary secondary septa (arrows) in the mutant lungs (Figure 1C). No apparent defects in other organs were found in *Vangl2^f/f^; Sox9^Cre/+^* mice (Figure 1—figure supplement 6). The primary septa in *Vangl2^f/f^; Sox9^Cre/+^* lungs underwent a similar thinning process in the first three days of postnatal life. However, the primary septa in mutant lungs failed to engender subsequent modification and retained a relatively thin appearance (Figure 1B). These results suggest that loss of PCP signaling in the distal lung cells has a profound influence on the normal progression and development of the primary septa to generate secondary septa. Only a very small number of primordial secondary septa could be detected in *Vangl2^f/f^; Sox9^Cre/+^* lungs. As a result, *Vangl2*-deficient lungs contained enlarged saccules (Figure 1B, Figure 1—figure supplement 7) with an increased mean linear intercept (MLI, a measure of air space size) (Figure 1D). Disruption of secondary septa formation in the absence of PCP signaling in the distal lung epithelium likely led to respiratory failure and neonatal lethality.

### Inactivation of *Vangl2* in interstitial fibroblasts/myofibroblasts disrupts alveolar formation, resembling loss of epithelial *Vangl2*

Most studies on PCP signaling have focused on how this pathway controls collective cell behavior in epithelial sheets. Whether PCP signaling functions in mesenchymal cells is underexplored. To determine if PCP signaling is required in the lung mesenchyme for alveolar formation, we generated *Vangl2^f/f^; Dermo1^Cre/+^* and *Vangl2^f/f^; Pdgfra^Cre/+^* mice. *Dermo1* (*Twist2*) is broadly expressed in mesenchymal cells while *Pdgfra*, which encodes a receptor for the platelet-derived growth factor A (PDGFA), is expressed in interstitial fibroblasts and induces smooth muscle actin (SMA) expression, a hallmark of myofibroblasts. Thus, Cre expression from the *Dermo1* locus (*Dermo1^Cre^*) (Yu et al., 2003) would eliminate *Vangl2* in most mesenchymal cells whereas Cre expression from the *Pdgfra* locus (*Pdgfra^Cre^*) (Roesch et al., 2008) would inactivate *Vangl2* in interstitial fibroblasts/myofibroblasts and disrupt PCP signaling.

We found that *Vangl2^f/f^; Dermo1^Cre/+^* and *Vangl2^f/f^; Pdgfra^Cre/+^* mice displayed lung phenotypes (Figure 1E, F, G, H and I) resembling those in *Vangl2^f/f^; Sox9^Cre/+^* mice. Secondary septa failed to form in *Vangl2^f/f^; Dermo1^Cre/+^* or *Vangl2^f/f^; Pdgfra^Cre/+^* mice, saccules were enlarged with an increased mean linear intercept, and many of the mutants failed to survive beyond postnatal day 7 (Figure 1E, F, G, H and I). We noticed that more *Vangl2^f/f^; Pdgfra^Cre/+^* survivors with less severe alveolar defects were observed than *Vangl2^f/f^; Sox9^Cre/+^* survivors. This could be due to differential Cre activity or differential contributions of epithelial and mesenchymal PCP signaling to alveolar formation. Loss of one copy of *Vangl1* did not exacerbate the lung phenotypes in *Vangl2^f/f^; Pdgfra^Cre/+^* mice, highlighting the major role of *Vangl2* in interstitial fibroblasts/myofibroblasts during alveolar formation. We conclude that PCP signaling operates in both the lung epithelium and mesenchyme and each process is essential for secondary septa formation by controlling distinct aspects of alveologenesis.

### Alveolar defects in mice carrying *Ror2*-deficient lung epithelium or interstitial fibroblasts/myofibroblasts recapitulate phenotypes caused by *Vangl2* loss in the corresponding compartment

**Figure 2.**
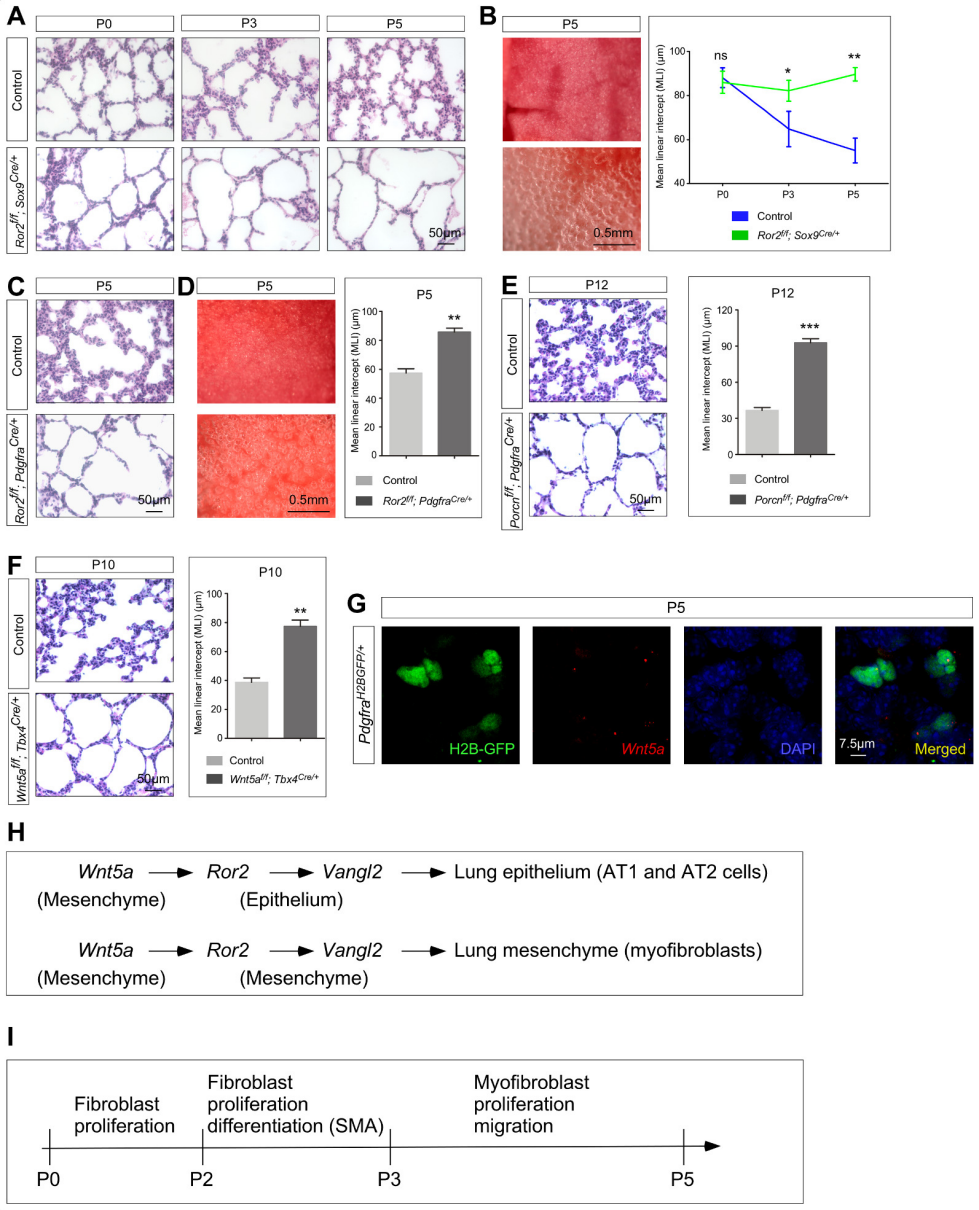
*Ror2* is required in both the lung epithelium and mesenchyme while *Wnt5a* is required in the lung mesenchyme for alveolar formation. (**A**) Hematoxylin and eosin-stained lung sections of wild-type and *Ror2^f/f^; Sox9^Cre/+^* mice at different postnatal (P) stages. Histological analysis revealed the presence of enlarged saccules starting at P3 in *Ror2*-deficient lungs. (**B**) Surface view of dissected lungs from wild-type and *Ror2^f/f^; Sox9^Cre/+^* mice at P5. Larger saccules were found in *Ror2* mutant lungs induced by *Sox9^Cre^* and the MLI was correspondingly increased in *Ror2*-deficient lungs. (**C**) Hematoxylin and eosin-stained lung sections of wild-type and *Ror2^f/f^; Pdgfra^Cre/+^* mice at P5. Histological analysis confirmed the presence of enlarged saccules starting at P3 in *Ror2*-deficient lungs. (**D**) Surface view of dissected lungs from wild-type and *Ror2^f/f^; Pdgfra^Cre/+^* mice at P5. Larger saccules were found in *Ror2* mutant lungs induced by *Pdgfra^Cre^* with an increased MLI. (**E**) Hematoxylin and eosin-stained lung sections of wild-type and *Porcn^f/f^; Pdgfra^Cre/+^* mice at P12. Larger saccules were found in *Porcn* mutant lungs induced by *Pdgfra-Cre* with an increased MLI. (**F**) Hematoxylin and eosin-stained lung sections of wild-type and *Wnt5a^f/f^; Tbx4^Cre/+^* mice at P10. Larger saccules were found in *Wnt5a* mutant lungs induced by *Tbx4-Cre* with an increased MLI. (**G**) Combined in situ hybridization (PLISH)/immunohistochemistry on lung sections of *Pdgfra^H2BGFP/+^* mice to examine *Wnt5a* expression. *Wnt5a* mRNA was mainly detected in myofibroblasts (H2BGFP^+^) and not in the lung epithelium. (**H**) Schematic diagram of a *Wnt5a–Ror2–Vangl2* axis that functions in both the lung epithelium and mesenchyme to regulate alveologenesis. (**I**) Schematic diagram of the temporal sequence of fibroblast/myofibroblast proliferation, differentiation and migration.

To reveal the signaling cascade that controls *Vangl2* function and subsequently alveolar formation, we tested the role of *Ror2* (receptor tyrosine kinase-like orphan receptor 2), which has been implicated in PCP signaling in other tissues (Ho et al., 2012). To this end, we generated *Ror2^f/f^; Sox9^Cre/+^* mice and anticipated that a floxed allele of *Ror2* (*Ror2^f^*) (Ho et al., 2012) would be converted to a null allele in the distal lung epithelium by *Sox9-Cre*. If ROR2 is responsible for mediating VANGL2 activity, we predict that loss of *Ror2* in the distal lung epithelium in *Ror2^f/f^; Sox9^Cre/+^* mice would result in alveolar defects similar to those in *Vangl2^f/f^; Sox9^Cre/+^* mice. Indeed, *Ror2^f/f^; Sox9^Cre/+^* mice displayed neonatal lethality and their lungs failed to produce secondary septa (Figure 2A and B). A detailed phenotypic analysis (Figure 2A and B) confirmed the similarity of lung phenotypes between *Ror2^f/f^; Sox9^Cre/+^* and *Vangl2^f/f^; Sox9^Cre/+^* mice.

**Figure 2—figure supplement 1.**
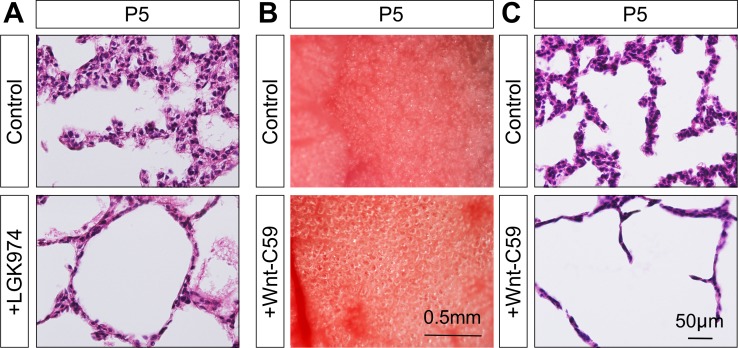
Inhibition of PORCUPINE activity leads to alveolar defects. (**A**) Hematoxylin and eosin-stained lung sections of mice at postnatal (P) day 5, which were treated with vehicles (control) or LGK974 (PORCUPINE inhibitor) at birth. (**B**) Surface view of dissected lungs from mice at P5, which were treated with vehicles or Wnt-C59 (PORCUPINE inhibitor) at birth. Larger saccules were found in lungs treated with Wnt-C59 compared to controls. (**C**) Histological analysis confirmed the presence of enlarged saccules in Wnt-C59-treated lungs.

**Figure 2—figure supplement 2.**
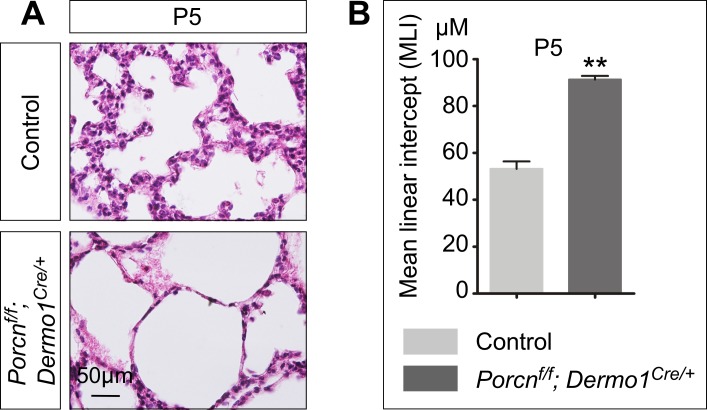
Elimination of mesenchymal *Porcupine* affects alveolar formation. (**A**) Hematoxylin and eosin-stained lung sections of control and *Porcn^f/f^; Dermo1^Cre/+^* mice at postnatal (P) day 5. Histological analysis revealed the presence of enlarged saccules in the absence of mesenchymal *Porcn* induced by *Dermo1-Cre*. (**B**) Measurement of the mean linear intercept (MLI) in control and *Porcn^f/f^; Dermo1^Cre/+^* lungs at P5. The MLI was increased in the mutant lungs. All values are mean SEM. (**) p<0.01 (unpaired Student’s *t*-test).

**Figure 2—figure supplement 3.**
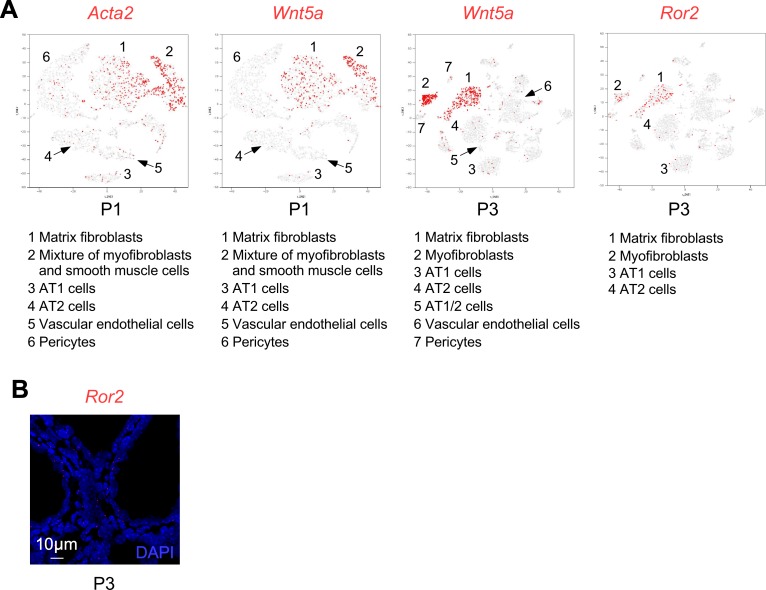
Single-cell RNA-Seq reveals expression of *Wnt5a* in myofibroblasts. (**A**) Single-cell RNA-Seq data of mouse lung cells at postnatal (P) 1 and 3 were downloaded from LungMAP, which is an NIH-funded consortium to generate publicly available data. Expression of *Acta2* (*SMA*), *Wnt5a* and *Ror2* (red color) was superimposed with predicted mouse lung cell populations on a t-SNE plot. At P1, *Wnt5a* is expressed in airway/vascular smooth muscle cells (ACTA2^+^) in addition to alveolar fibroblasts (ACTA2^–^). At P3, *Wnt5a* is highly expressed in fibroblasts and myofibroblasts but not in alveolar epithelial cells, endothelial cells or pericytes. At P3, *Ror2* is also highly expressed in fibroblasts and myofibroblasts with lower levels of expression in alveolar epithelial cells. (**B**) In situ hybridization (PLISH) on lung sections of wild-type mice to examine *Ror2* expression. *Ror2* was broadly expressed in both the lung epithelium and mesenchyme.

**Figure 2—figure supplement 4.**
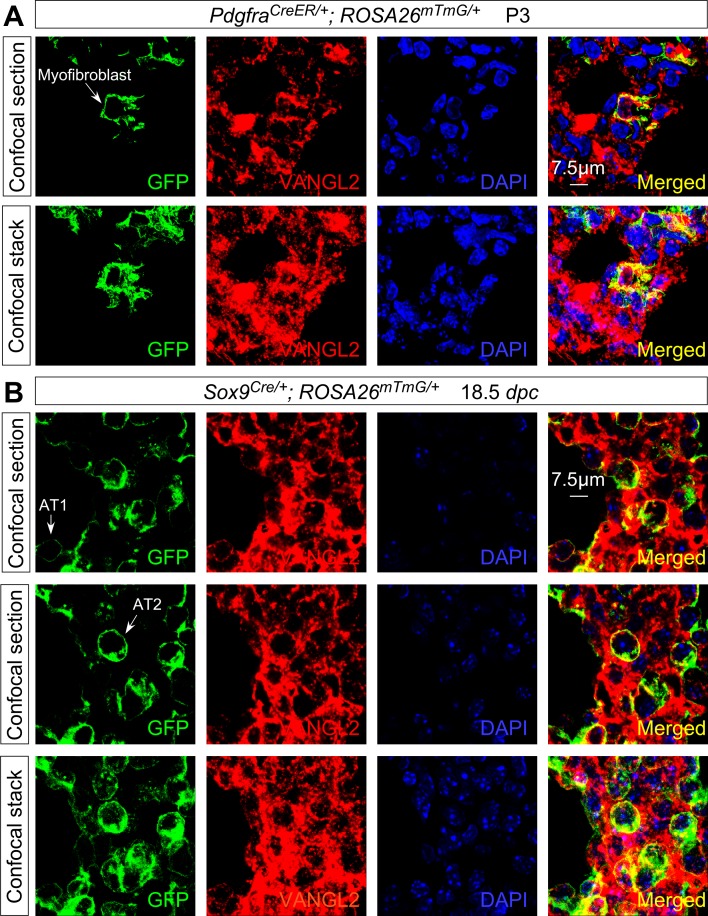
VANGL2 is not asymmetrically localized in lung epithelial and mesenchymal cells. (**A**) Immunostaining of lung sections collected from *Pdgfra^CreER^/+; ROSA26^mTmG/+^* mice at postnatal (P) day 3. Leaky expression of CreER led to GFP-labeling (from the *ROSA26^mTmG/+^* locus) of individual myofibroblasts. No apparent asymmetric distribution of VANGL2 was detected in myofibroblasts. This was revealed by examining each confocal section. The confocal stack represents the compilation of all confocal sections for a given myofibroblast examined. (**B**) Immunostaining of lung sections collected from *Sox9^Cre/+^; ROSA26^mTmG/+^* mice at 18.5 *days post coitus* (*dpc*). Activation of GFP by *Sox9-Cre* resulted in labeling of alveolar type I (AT1) and type II (AT2) cells, which could be distinguished by morphology. No apparent asymmetric distribution of VANGL2 was detected in AT1 or AT2 cells. This was revealed by examining each confocal section. The confocal stack represents the compilation of all confocal sections for a given AT1 or AT2 cell examined. The extended morphology of AT1 cells placed an inherent limitation on the proportion of cell membrane that could be visualized in a given AT1 cell. Nevertheless, in regions that could be discerned, especially cell membranes surrounding the nucleus, no asymmetric distribution of VANGL2 was revealed.

We also produced *Ror2^f/f^; Pdgfra^Cre/+^* mice to remove *Ror2* in lung interstitial fibroblasts/myofibroblasts. As predicted by our model of a *Ror2–Vangl2* cascade, these mice died postnatally due to alveolar defects (Figure 2C and D), resembling phenotypes observed in *Vangl2^f/f^; Pdgfra^Cre/+^* mice. Taken together, these studies established a key role of *Ror2* in activating *Vangl2* in either alveolar epithelial cells or mesenchymal myofibroblasts. The downstream events mediated by *Ror2–Vangl2* are critical for secondary septa formation and alveolar formation.

### Mesenchymal *Wnt5a* signaling to lung epithelium and mesenchyme is essential for alveolar development

A key question in further understanding how PCP signaling controls alveolar development is to uncover the signals that trigger a *Ror2–Vangl2* cascade. Several non-canonical Wnt ligands are expressed in the lungs (Li et al., 2015) and are candidates for activating a *Ror2–Vangl2* axis. We first manipulated the activity of *Porcupine* (*Porcn*) in the lung, which is required for the release of all WNT proteins from *Wnt*-producing cells (Cadigan and Peifer, 2009; MacDonald et al., 2009). Consistent with the involvement of *Wnt* signaling in alveolar development, treatment of neonatal mice with Wnt-C59 (Proffitt et al., 2013) or LGK974 (WNT-974) (Liu et al., 2013), inhibitors of PORCUPINE, resulted in alveolar defects (Figure 2—figure supplement 1). Moreover, inactivation of *Porcn* in the lung mesenchyme by creating *Porcn^f/f^; Dermo1^Cre/+^* mice or in lung myofibroblasts by generating *Porcn^f/f^; Pdgfra^Cre/+^* mice also led to alveolar defects (Figure 2—figure supplement 2, Figure 2E). In this setting, a floxed allele of *Porcn* (*Porcn^f^*) (Liu et al., 2012) was removed by *Dermo1-Cre* or *Pdgfra-Cre*. These results support the notion that Wnt signals in the lung mesenchyme initiate PCP signaling for alveolar development.

Among the Wnt ligands, WNT5A is a likely candidate in activating a *Ror2–Vangl2* cascade in the lung mesenchyme. *Wnt5a* transcripts were detected in fibroblasts and myofibroblasts (Figure 2—figure supplement 3) and a very small number of alveolar epithelial cells by single cell RNA-Seq analysis of postnatal lungs (Guo et al., 2019). Moreover, *Wnt5a*-deficient lungs exhibit a shortened trachea (Li et al., 2002; Kishimoto et al., 2018), consistent with defective PCP signaling. Whether *Wnt5a* controls PCP signaling during alveolar development is unknown. We first performed PLISH (proximity ligation in situ hybridization) (Nagendran et al., 2018) on lungs from *Pdgfra^H2BGFP^* mice (Hamilton et al., 2003) to examine the expression patterns of *Wnt5a* in postnatal lungs. Expression of the H2B-GFP fusion protein in the nucleus of *Pdgfra*-expressing cells (*Pdgfra^H2BGFP^*) unambiguously marked myofibroblasts. We found that *Wnt5a* mRNA was mainly expressed in myofibroblasts and barely any signal was detected in the lung epithelium (Figure 2G).

To test the role of *Wnt5a* in activating a *Ror2–Vangl2* cascade, we initially utilized *Sox9-Cre*, *Pdgfra-Cre* and *Dermo1-Cre* to convert a floxed allele of *Wnt5a* (*Wnt5a^f^*) (Ryu et al., 2013) into a null allele. Unfortunately, *Wnt5a^f/f^; Sox9^Cre/+^*, *Wnt5a^f/f^; Pdgfra^Cre/+^* and *Wnt5a^f/f^; Dermo1^Cre/+^* mice all died soon after birth due to craniofacial defects. The perinatal lethality prevented us from analyzing alveolar defects in these animals. To circumvent this problem, we employed *Tbx4-Cre* (Kumar et al., 2014) to inactivate *Wnt5a* selectively in the lung mesenchyme but not the mesenchyme of other tissues. *Wnt5a^f/f^; Tbx4^Cre/+^* mice were born alive but many displayed a smaller body size at postnatal day 4 or 5. Phenotypic analysis of *Wnt5a^f/f^; Tbx4^Cre/+^* lungs revealed defective alveolar formation (Figure 2F) albeit the alveolar defects were not as pervasive as those caused by loss of *Ror2* or *Vangl2*. In other experimental settings, we have found that *Tbx4-Cre* is less efficient in conditional gene inactivation in comparison to other Cre lines.

Taken together, our genetic studies have established a *Wnt5a–Ror2–Vangl2* axis in controlling alveolar formation (Figure 2H). In this process, mesenchymal *Wnt5a* triggers a *Ror2/Vangl2*–mediated cascade in both lung epithelium and mesenchyme.

In many studies where PCP signaling controls the collective cell behavior of epithelial sheets, PCP components display asymmetric distribution within individual epithelial cells. This is proposed to underlie the coordination between epithelial cells through organizing the cytoskeleton along the same axis among individual cells. We thus examined VANGL2 distribution in either lung epithelial cells or mesenchymal myofibroblasts. To our surprise, we found no apparent asymmetric localization of VANGL2 in alveolar type I cells, alveolar type II cells or myofibroblasts (Figure 2—figure supplement 4). This led to our new model in which a *Wnt5a–Ror2–Vangl2* axis controls cellular properties of individual lung cells regardless of asymmetric activation of PCP components. We speculate that this cascade sets in motion PDGF signaling from alveolar type I and type II cells, morphological changes of alveolar type I cells and migration of myofibroblasts, all of which are required for alveolar formation. We will test these hypotheses in subsequent sections.

### Loss of epithelial *Vangl2* leads to defective PDGF signal reception in mesenchymal myofibroblasts

**Figure 3.**
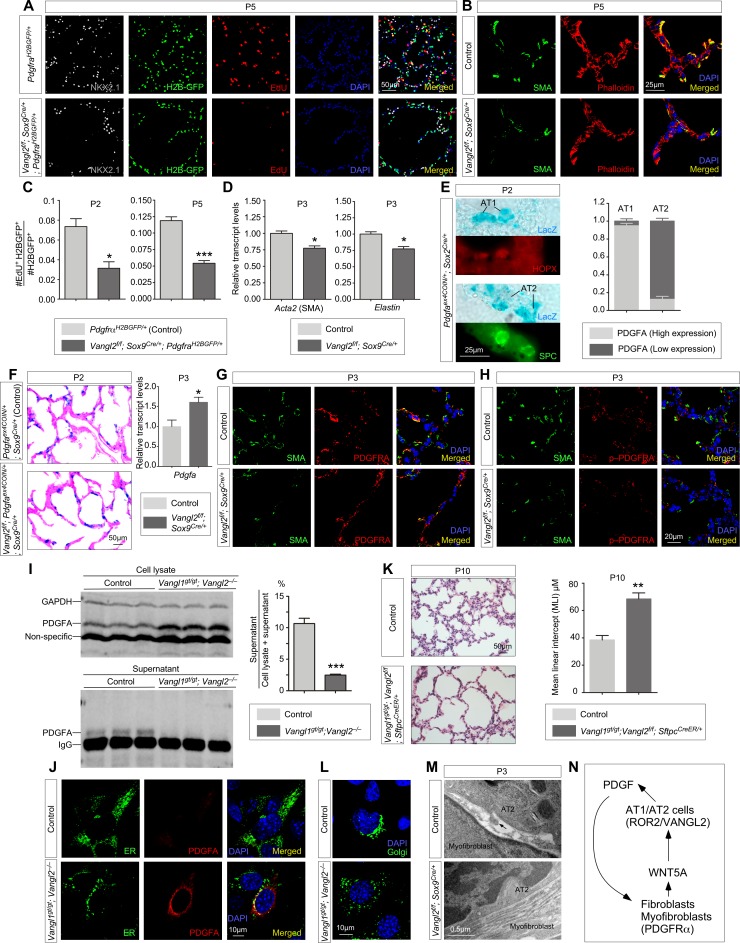
*Vangl2* is required for PDGF ligand trafficking/release from PDGF-producing cells and subsequently PDGF signal reception. (**A**) Immunostaining of lung sections collected from *Pdgfra^H2BGFP/+^* (control) and *Vangl2^f/f^; Sox9^Cre/+^; Pdgfra^H2BGFP/+^* mice injected with EdU at postnatal (P) day 5. Lung epithelial cells were distinguished by NKX2.1 staining while myofibroblasts were marked by H2B-GFP from the *Pdgfra* locus (*Pdgfra^H2BGFP^*). The number of EdU^+^ cells was reduced in *Vangl2^f/f^; Sox9^Cre/+^; Pdgfra^H2BGFP/+^* lungs compared to controls. (**B**) Immunostaining of lung sections collected from control and *Vangl2^f/f^; Sox9^Cre/+^* mice at P5. Organization of the cytoskeleton and stress fibers was disrupted and SMA at the prospective sites of secondary septation was sparse in *Vangl2*-deficient lungs. (**C**) Quantification of myofibroblast proliferation in *Pdgfra^H2BGFP/+^* (control) and *Vangl2^f/f^; Sox9^Cre/+^; Pdgfra^H2BGFP/+^* lungs at P2 and P5. The rate of myofibroblast proliferation was calculated as the ratio of the number of EdU^+^ myofibroblasts (EdU^+^H2BGFP^+^) to the number of myofibroblasts (H2BGFP^+^). An apparent reduction in the percentage of proliferating myofibroblasts was detected in *Vangl2^f/f^; Sox9^Cre/+^; Pdgfra^H2BGFP/+^* lungs compared to controls (n = 3 for each group) at P2 and P5. (**D**) qPCR analysis of *Acta2* (*SMA*) and *Elastin* in control and *Vangl2^f/f^; Sox9^Cre/+^* lungs at P3. The mRNA levels of *Acta2* and *Elastin* were significantly reduced in the absence of epithelial *Vangl2* induced by *Sox9-Cre* (n = 3 for each group). (**E**) Immunostaining of lung sections collected from *Pdgfa^ex4COIN/+^; Sox2^Cre/+^* mice at P2. *β-galactosidase* (*LacZ*) was induced in PDGFA-producing cells by *Sox2-Cre*. LacZ-staining (blue) was followed by immunostaining against HOPX (marker for AT1 cells) and SPC (marker for AT2 cells). LacZ-positive cells also expressed either HOPX or SPC. The number of AT1 or AT2 cells that harbored either high or low PDGFA (LacZ) was counted. (**F**) LacZ-staining (blue) of lung sections collected from *Pdgfa^ex4COIN/+^; Sox9^Cre/+^* (control) and *Vangl2^f/f^; Pdgfa^ex4COIN/+^; Sox9^Cre/+^* mice at P2. The slides were counterstained with eosin (red). No difference in the intensity of LacZ (+) cells in the lung was found in these two mouse lines. The mRNA levels of *Pdgfa* in control and mutant lungs were determined by qPCR. (**G**) Immunostaining of lung sections collected from control and *Vangl2^f/f^; Sox9^Cre/+^* mice at P3. No difference in PDGFRA expression levels in individual myofibroblasts was noted between control and *Vangl2^f/f^; Sox9^Cre/+^* lungs. (**H**) Immunostaining of lung sections collected from control and *Vangl2^f/f^; Sox9^Cre/+^* mice at P3. A significant reduction in the levels of phosphorylated (p) PDGFRA in individual myofibroblasts was found in *Vangl2^f/f^; Sox9^Cre/+^* lungs compared to controls. (**I**) Western blot analysis of cell lysates and supernatants from control and *Vangl1^gt/gt^; Vangl2^–/–^* cells lentivirally transduced with PDGFA-expressing constructs. The amount of PDGFA released into the media was significantly reduced in *Vangl1^gt/gt^; Vangl2^–/–^* cells compared to controls (n = 3 for each group). GAPDH served as a loading control. We noticed that the amount of secreted proteins (normalized to the cell number) from *Vangl1/2* mutant cells was reduced compared to controls. This suggests a general defect in protein processing/secretion in the absence of *Vangl1/2*. In this case, it is possible that other secreted ligands could also impact alveolar development. (**J**) Immunostaining of controls and *Vangl1^gt/gt^; Vangl2^–/–^* cells lentivirally transduced with PDGFA-expressing constructs. Endoplasmic reticulum (ER) was marked by mEmerald-ER-5. (**K**) Hematoxylin and eosin-stained lung sections of wild-type and *Vangl1^gt/gt^; Vangl2^f/f^; Sftpc^CreER/+^* mice injected with tamoxifen and collected at P10. Enlarged saccules were found in *Vangl1^gt/gt^; Vangl2^f/f^; Sftpc^CreER/+^* lungs in comparison with controls. (**L**) Immunostaining of controls and *Vangl1^gt/gt^; Vangl2^–/–^* cells. The Golgi stacks were dispersed in *Vangl1^gt/gt^; Vangl2^–/–^* cells compared to controls. Golgi was marked by mEmerald-Golgi-7. (**M**) Transmission electron micrographs of lungs from wild-type and *Vangl2^f/f^; Sox9^Cre/+^* mice at P3. Cellular extension (arrow) from alveolar type II cells to myofibroblasts was observed in control lungs but were absent in *Vangl2^f/f^; Sox9^Cre/+^* lungs. (**N**) Schematic diagram of a positive feedback loop between WNT5A and PDGF to generate a pool of fibroblasts/myofibroblasts for alveologenesis. All values are mean ± SEM. (*) p<0.05; (**) p<0.01; (***) p<0.001; ns, not significant (unpaired Student’s *t*-test).

Lack of secondary septa in *Vangl2^f/f^; Sox9^Cre/+^* mice was associated with thin primary septa. This prompted us to investigate whether proliferation and function of myofibroblasts were affected in the absence of epithelial PCP signaling in *Vangl2^f/f^; Sox9^Cre/+^* lungs. In wild-type lungs, fibroblast proliferation can be detected at P0; this is followed by SMA expression at P2/3 and subsequent migration while proliferation continues (Figure 2I). Indeed, we found that interstitial fibroblasts that expressed PDGFRA failed to proliferate as revealed by reduced EdU incorporation in *Vangl2^f/f^; Pdgfra^H2BGFP/+^; Sox9^Cre/+^* mice in comparison with *Pdgfra^H2BGFP/+^* mice (controls) (Figure 3A, Figure 3—figure supplement 1). Myofibroblasts were labeled by H2BGFP. Moreover, myofibroblasts (SMA^+^) in *Vangl2^f/f^; Sox9^Cre/+^* lungs failed to migrate to the prospective sites of secondary septa formation (Figure 3B). Decreased myofibroblast proliferation first appeared at postnatal day 2 when PDGFRA^+^ interstitial fibroblasts started to express SMA and became myofibroblasts. This suggests that expansion of fibroblasts prior to SMA expression and migration was disrupted in the absence of epithelial *Vangl2*. By postnatal day 3, the number of PDGFRA^+^ interstitial fibroblasts had vastly decreased in the mutant lungs (Figure 3C). This was associated with a major reduction in SMA^+^ cells. Myofibroblasts secrete elastin and collagen, which play an important role in promoting secondary septa formation. As expected, both SMA and elastin production was significantly curtailed in *Vangl2^f/f^; Sox9^Cre/+^* lungs (Figure 3D).

**Figure 3—figure supplement 1.**
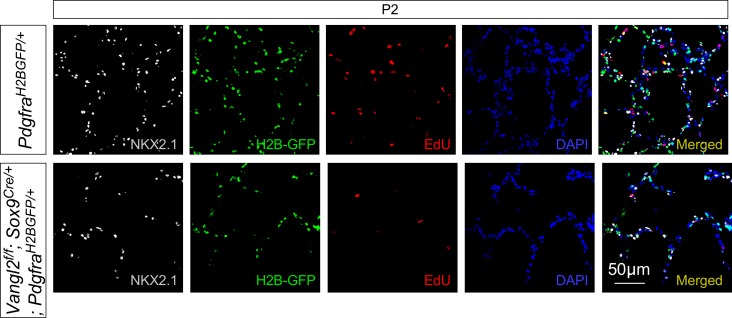
Loss of epithelial *Vangl2* leads to reduced myofibroblast proliferation. Immunostaining of lung sections collected from *Pdgfra^H2BGFP/+^* (control) and *Vangl2^f/f^; Sox9^Cre/+^; Pdgfra^H2BGFP/+^* mice injected with EdU at postnatal (P) day 2. Lung epithelial cells were distinguished by NKX2.1 staining while myofibroblasts were marked by H2B-GFP from the *Pdgfra* locus (*Pdgfra^H2BGFP^*). The number of EdU^+^ cells was reduced in *Vangl2^f/f^; Sox9^Cre/+^; Pdgfra^H2BGFP/+^* lungs compared to controls. Note that quantification of proliferating myofibroblasts (EdU^+^H2BGFP^+^) is shown in Figure 3C.

**Figure 3—figure supplement 2.**
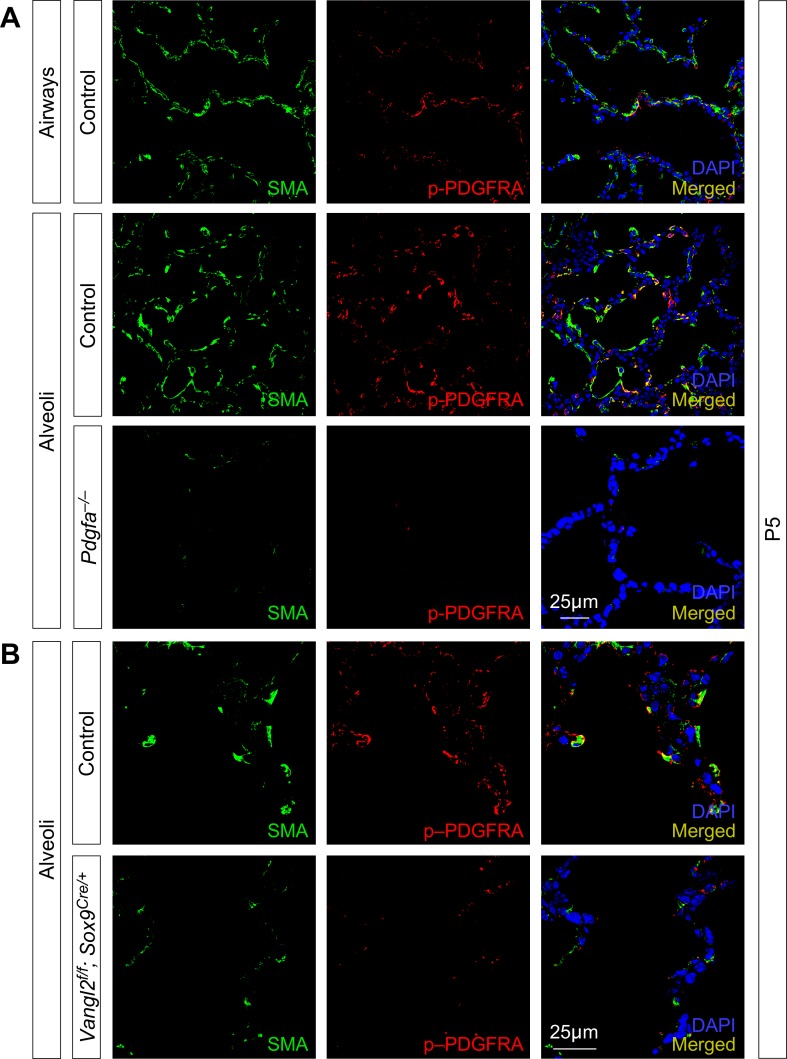
Removal of epithelial *Vangl2* results in reduced levels of phosphorylated PDGFRA. (**A**) Immunostaining of lung sections collected from control and *Pdgfa^ex4COIN/ex4COIN^; Sox2^Cre/+^* (abbreviated as *Pdgfa^–/–^*) mice at postnatal (P) day 5. PDGFRA^+^ myofibroblasts were absent in *Pdgfa^–/–^* lungs. Accordingly, phosphorylated (p) PDGFRA (p-PDGFRA) was barely detectable in *Pdgfa^–/–^* lungs. (**B**) Immunostaining of lung sections collected from control and *Vangl2^f/f^; Sox9^Cre/+^* mice at P5. A significant reduction in the levels of p-PDGFRA in individual myofibroblasts was found in *Vangl2^f/f^; Sox9^Cre/+^* lungs compared to controls. Smooth muscle actin (SMA) was primarily detected in smooth muscles and myofibroblasts.

**Figure 3—figure supplement 3.**
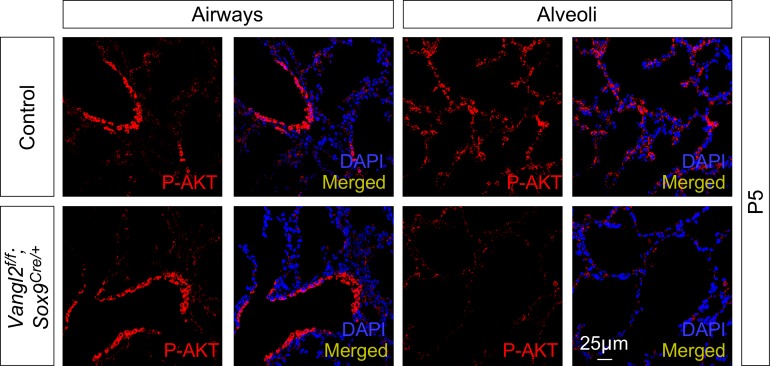
PDGF signal reception is reduced in lungs deficient in *Vangl1/2* signaling. Immunostaining of lung sections collected from control and *Vangl2^f/f^; Sox9^Cre/+^* mice at postnatal (P) day 5. While phosphorylated (p) AKT (p-AKT) showed no difference between control and *Vangl2^f/f^; Sox9^Cre/+^* lungs in the airways, p-AKT levels were significantly reduced in the alveoli of *Vangl2^f/f^; Sox9^Cre/+^* mice.

**Figure 3—figure supplement 4.**
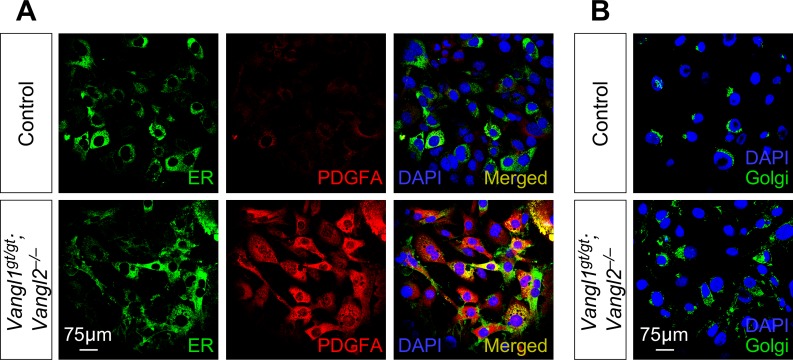
Accumulation of PDGF ligand in the secretory pathway in the absence of VANGL1/2. (**A**) Immunostaining of controls and *Vangl1^gt/gt^; Vangl2^–/–^* cells lentivirally transduced with PDGFA-expressing constructs. Endoplasmic reticulum (ER) was marked by mEmerald-ER-5. The levels of PDGFA were significantly increased in the secretory pathway of *Vangl1/2*-deficient cells. (**B**) Immunostaining of controls and *Vangl1^gt/gt^; Vangl2^–/–^* cells lentivirally transduced with PDGFA-expressing constructs. Golgi was marked by mEmerald-Golgi-7. The Golgi stacks were dispersed in *Vangl1/2*-deficient cells.

**Figure 3—figure supplement 5.**
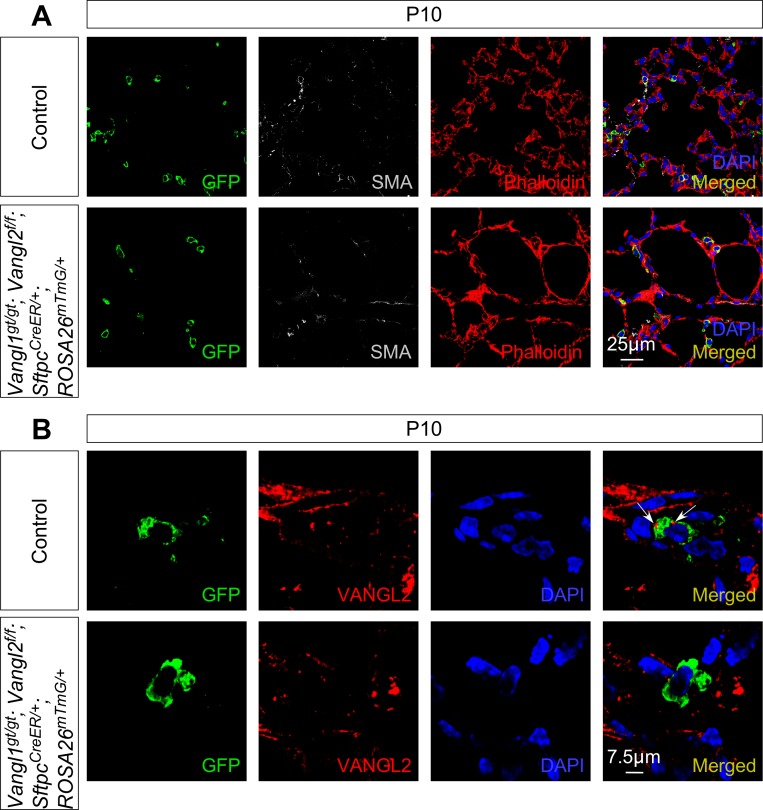
Loss of *Vangl1/2* in alveolar type II cells results in alveolar defects. (**A**) Immunostaining of lung sections collected from control and *Vangl1^gt/gt^; Vangl2^f/f^; Sftpc^CreER/+^; ROSA26^mTmG/+^* mice at postnatal (P) day 10. Tamoxifen was administered at P0. Inactivation of *Vangl1/2* in alveolar type II (AT2) cells led to alveolar defects. (**B**) Immunohistochemical analysis of lung sections collected from control and *Vangl1^gt/gt^; Vangl2^f/f^; Sftpc^CreER/+^; ROSA26^mTmG/+^* mice revealed loss of VANGL2 in AT2 cells (GFP^+^) in the mutant lungs.

**Figure 3—figure supplement 6.**
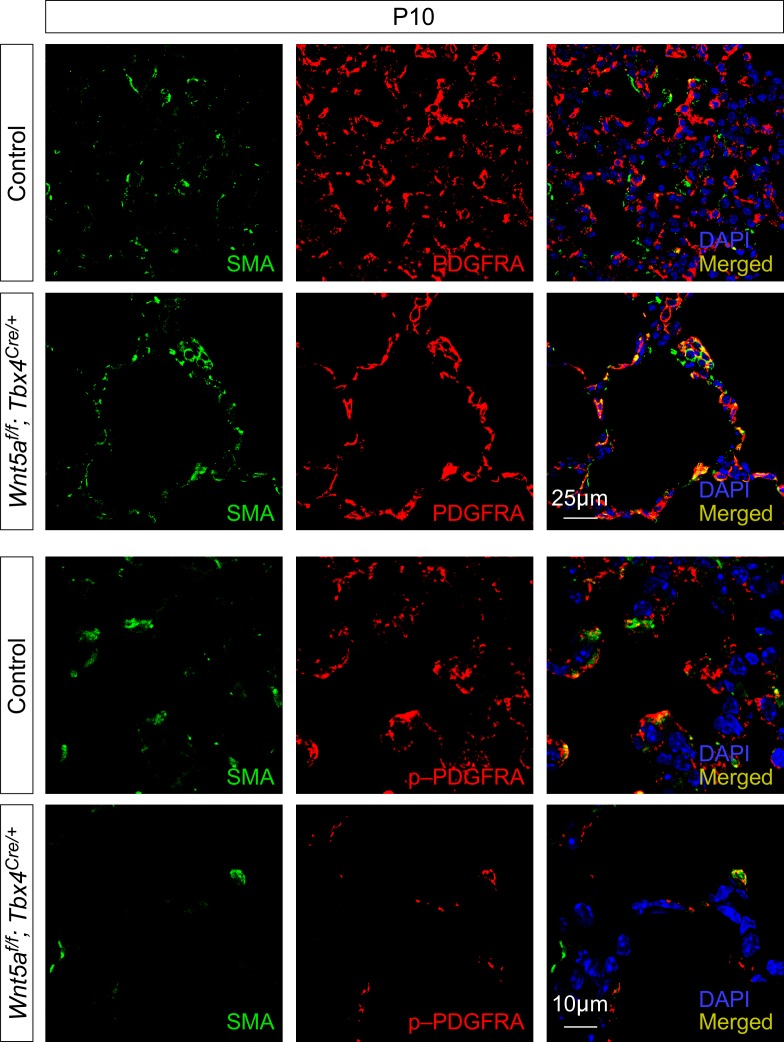
Removal of mesenchymal *Wnt5a* results in reduced levels of phosphorylated PDGFRA. Immunostaining of lung sections collected from control and *Wnt5a^f/f^; Tbx4^Cre/+^* mice at postnatal (P) day 10. A significant reduction in the levels of phosphorylated (p) PDGFRA in individual myofibroblasts was found in *Wnt5a^f/f^; Tbx4^Cre/+^* lungs compared to controls. By contrast, the levels of PDGFRA in individual myofibroblasts were unaffected.

**Figure 3—figure supplement 7.**
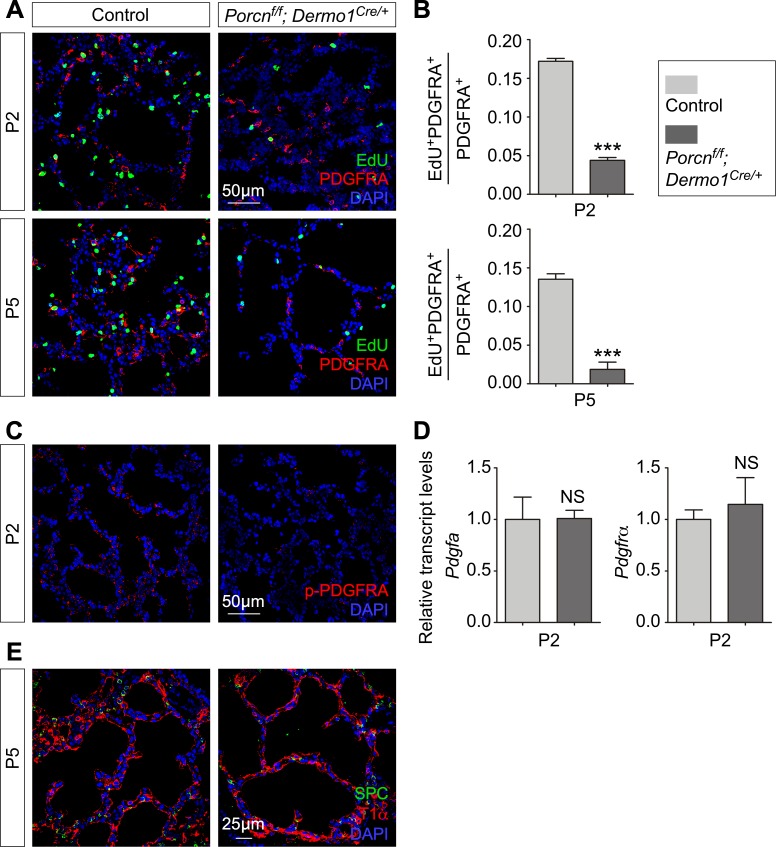
Mesenchymal *Wnt* signaling is required for fibroblast/myofibroblast proliferation but does not affect differentiation of alveolar epithelial cells. (**A, C, E**) Immunostaining of lung sections from control and *Porcn^f/f^; Dermo1^Cre/+^* mice at postnatal (P) day 2 and 5. The number of proliferating myofibroblasts (EdU^+^PDGFRA^+^) was reduced in the absence of mesenchymal *Wnt* signaling (shown in A). Quantification (n = 3 for each group) was shown in (**B**). Phosphorylated (p) PDGFRA was significantly reduced in the mutant lungs (shown in C) while the transcript levels of *Pdgfa* and *Pdgfra* were unaltered (n = 3 for each group) (shown in **D**). Loss of mesenchymal *Wnt* signaling had no effect on differentiation of alveolar epithelial cells (shown in **E**). All values are mean SEM. (***) p<0.001; ns, not significant (unpaired Student’s *t*-test).

**Figure 3—figure supplement 8.**
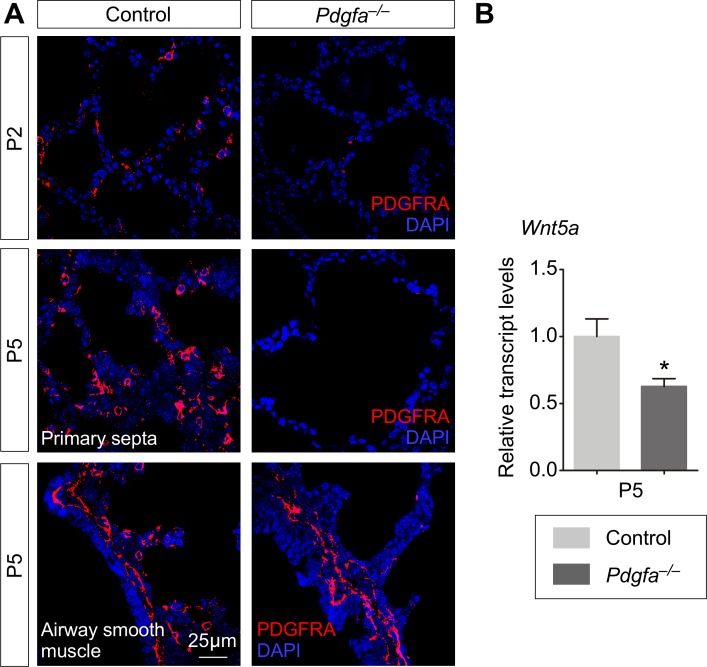
PDGF signaling is required for preserving a pool of WNT5A-secreting myofibroblasts. (**A**) Immunostaining of lung sections collected from control and *Pdgfa^ex4COIN/ex4COIN^; Sox2^Cre/+^* (abbreviated as *Pdgfa^–/–^*) mice at postnatal (P) day 2 and 5. PDGFRA^+^ myofibroblasts were absent in *Pdgfa^–/–^* lungs. This indicates that the source of WNT5A (produced from myofibroblasts) for alveolar development was depleted. By contrast, PDGFRA^+^ smooth muscle cells in the airway were unaffected in the absence of PDGF signaling. (**B**) qPCR analysis of *Wnt5a* transcript levels in control and *Pdgfa^–/–^* lungs (n = 4) at P5. *Wnt5a* mRNA levels were reduced in the absence of PDGF signaling. All values are mean SEM. (*) p<0.05. (unpaired Student’s *t*-test).

Given the reciprocal interaction between the lung epithelium and mesenchyme, we speculate that aberrant myofibroblast development in *Vangl2^f/f^; Sox9^Cre/+^* lungs stems from malfunction of the lung epithelium. We first tested whether perturbation of pathways that signal from the lung epithelium to the mesenchyme would have caused anomalous alveolar formation in *Vangl2^f/f^; Sox9^Cre/+^* mice and found that PDGF signaling was disrupted in myofibroblasts in these animals. PDGFA is produced primarily in alveolar type I and type II cells and signals to *Pdgfra*-expressing fibroblasts/myofibroblasts (Chen and Krasnow, 2012; Gouveia et al., 2017) to regulate their proliferation and migration. While loss of either *Pdgfa* (Boström et al., 1996; Lindahl et al., 1997) or *Pdgfra* (Sun et al., 2000) can result in loss of secondary septa (Noskovičová et al., 2015), we found no difference in PDGFA ligand production between control and *Vangl2^f/f^; Sox9^Cre/+^* lungs by both qPCR analysis and in situ hybridization. This conclusion was substantiated by a PDGFA reporter mouse line (*Pdgfa^ex4COIN^*) (Andrae et al., 2014) that faithfully recapitulates the spatial and temporal expression of *Pdgfa* since no reliable PDGFA antibody is available to detect endogenous PDGFA in tissues (Gouveia et al., 2017; Andrae et al., 2014). In *Pdgfa^ex4COIN/+^; Sox2^Cre/+^* mice, Cre recombinase was broadly expressed and activated *β-galactosidase* (*lacZ*) expression (from the *Pdgfa^ex4COIN^* allele) in *Pdgfa*-expressing cells, including AT1 (HOPX^+^) and AT2 (SPC^+^) cells (Figure 3E). *LacZ* expression in *Pdgfa*-expressing epithelial cells is indicative of PDGFA levels. We found that PDGFA was expressed more frequently in AT1 cells than AT2 cells and PDGFA expression levels were higher in AT1 cells than AT2 cells (Figure 3E), consistent with single-cell RNA-Seq data (Wang et al., 2018). We generated *Vangl2^f/f^; Pdgfa^ex4COIN/+^; Sox9^Cre/+^* to examine the effects of epithelial VANGL2 on PDGFA expression. PDGFA displayed a similar pattern and intensity between control and *Vangl2*-deficient lungs (Figure 3F), consistent with no reduction in *Pdgfa* transcript levels in the absence of *Vangl2* (Figure 3F). These results pointed to defective PDGF ligand trafficking, secretion or delivery in *Vangl2^f/f^; Sox9^Cre/+^* lungs after the PDGF ligand is made. It also predicts that PDGF fails to transduce its signal to mesenchymal myofibroblasts of *Vangl2^f/f^; Sox9^Cre/+^* lungs.

Consistent with our model, while the levels of PDGFRA in a single myofibroblast were unaffected (Figure 3G), phosphorylation of PDGFRA in individual myofibroblasts upon PDGFA ligand binding was significantly reduced in *Vangl2^f/f^; Sox9^Cre/+^* lungs (Figure 3H, Figure 3—figure supplement 2). In addition, activation of the downstream kinases, such as AKT (a serine-threonine kinase) (Demoulin and Essaghir, 2014), was drastically diminished in *Vangl2^f/f^; Sox9^Cre/+^* lungs in comparison with controls by immunostaining (Figure 3—figure supplement 3). Disrupted PDGF signal transduction in myofibroblasts led to reduced proliferation and migration. These findings support our model in which the PDGFA ligand produced in *Vangl2*-deficient alveolar epithelial cells fails to transduce its signal to mesenchymal myofibroblasts.

### Alveolar epithelial cells lacking VANGL2 fail to present the PDGF ligand to mesenchymal myofibroblasts

Our results indicate that loss of PCP signaling in alveolar epithelial cells disrupts their ability to transduce the PDGF signal to myofibroblasts in the mesenchyme. We envision that either the PDGF ligand failed to reach the cell surface of alveolar type I/II cells or failed to be delivered to target cells. To gain insight into this issue, we performed RNA-Seq analysis (Hrdlickova et al., 2017) of control and *Vangl2^f/f^; Sox9^Cre/+^* lungs collected at P2 and P5. Interestingly, pathways that regulate vesicular transport were perturbed in the mutant lungs (details below), suggesting their role in PDGF secretion. To explore this idea, we assayed intracellular trafficking and secretion of PDGFA in control and *Vangl1/2*-deficient cells. Control and *Vangl1/2*-deficient cells were derived from control and *Vangl1^gt/gt^; Vangl2^–/–^* embryos and subsequently lentivirally transduced with constructs that encode a fusion protein of PDGFA and 3xFLAG or GFP. Using this assay, we determined the amount of PDGFA-3xFLAG released from control and *Vangl1/2*-deficient cells. PDGFA-3xFLAG in the media incubated with *Vangl1/2*-deficient cells was significantly reduced (Figure 3I). These findings are consistent with a failure in vesicular transport and ligand release of PDGFA without *Vangl1/2*. Indeed, we found that the intracellular levels of PDGFA-GFP were increased in *Vangl1/2*-deficient cells (Figure 3J, Figure 3—figure supplement 4).

Consistent with our model in which the PDGF ligand fails to be released from alveolar epithelial cells in the absence of *Vangl1/2*, inactivation of *Vangl1/2* in alveolar type II cells (Surfactant protein C [SP-C or SPC]^+^ encoded by *Sftpc*) led to alveolar defects. We generated *Vangl1^gt/gt^; Vangl2^f/f^; Sftpc^CreER/+^* mice and administered tamoxifen to these animals at P2. Activation of CreER by tamoxifen in SPC^+^ cells (Lin et al., 2012) induced *Vangl2* removal. Analysis of lungs at P10 revealed areas of defective alveolar development that was associated with loss of *Vangl1/2* (Figure 3K, Figure 3—figure supplement 5). This supports a critical role of *Vangl1/2* in alveolar type II cells during alveologenesis.

We also discovered that the structure of the Golgi apparatus was altered in *Vangl1/2*-deficient cells. Compared to control cells in which the Golgi apparatus was properly polarized inside the cells, the Golgi stacks were dispersed in *Vangl1/2*-deficient cells (Figure 3L). We speculate that loss of the proper organization of the Golgi apparatus could contribute to disruption of the secretory pathways including PDGF ligand secretion.

Alveolar type II cells make direct cell-cell contacts with myofibroblasts via cellular processes that pass through the basement membrane (Figure 3M; Brody et al., 1982; Adamson and King, 1985). Such cell-cell contacts could facilitate PDGF signaling as supported by recent studies in which most, if not all, major signaling pathways are shown to require cell-cell contacts for ligand-receptor interactions and signal transduction (Kornberg, 2017; Kornberg, 2019). In this scenario, ablation of PCP signaling in alveolar epithelial cells would affect the actomyosin cytoskeleton, a common output of PCP signaling, and consequently either the formation or function of cellular processes that are required for transducing the PDGF signal. Interestingly, TEM studies revealed loss of cellular processes from alveolar type II cells in *Vangl2^f/f^; Sox9^Cre/+^* lungs, which were no longer in direct contact with myofibroblasts (Figure 3M).

Taken together, our studies support a model in which alveolar type I and type II cells utilize a *Wnt5a–Ror2–Vangl2* cascade to control PDGF ligand trafficking and release and contribute to secondary septa formation. We surmise that these effects are mediated by the actomyosin cytoskeleton.

### WNT5A and PDGF forms a positive feedback loop to generate a pool of fibroblasts/myofibroblasts required for alveologenesis

In our model, a *Wnt5a–Ror2–Vangl2* cascade operates in both the epithelial and mesenchymal compartments. We investigated how these signaling events are coordinated during postnatal lung development. We found that phosphorylation of PDGFRA in individual myofibroblasts upon PDGFA ligand binding was reduced in *Wnt5a^f/f^; Tbx4^Cre/+^* lungs (Figure 3—figure supplement 6), while the levels of PDGFRA were unaffected (Figure 3—figure supplement 6). Moreover, elimination of mesenchymal *Wnt* signaling (including WNT5A secretion from fibroblasts) prior to alveologenesis in *Porcn^f/f^; Dermo1^Cre/+^* lungs led to reduced PDGF signal transduction without affecting PDGF ligand production or differentiation of alveolar type I and type II cells (Figure 3—figure supplement 7). This suggests that loss of mesenchymal *Wnt5a* compromises PDGF signal reception but not ligand production in fibroblasts/myofibroblasts. Conversely, loss of PDGF ligand resulted in depletion of WNT5A-producing fibroblasts/myofibroblasts and a reduction in *Wnt5a* transcript levels (Figure 3—figure supplement 8). These results support a positive feedback loop between WNT5A and PDGFA to generate an adequate number of fibroblasts/myofibroblasts required for alveologenesis (Figure 3N).

### Components and regulators of the actomyosin cytoskeleton and vesicular transport show differential expression in *Vangl2* and *Ror2* mutant lungs by RNA-Seq analysis

**Figure 4.**
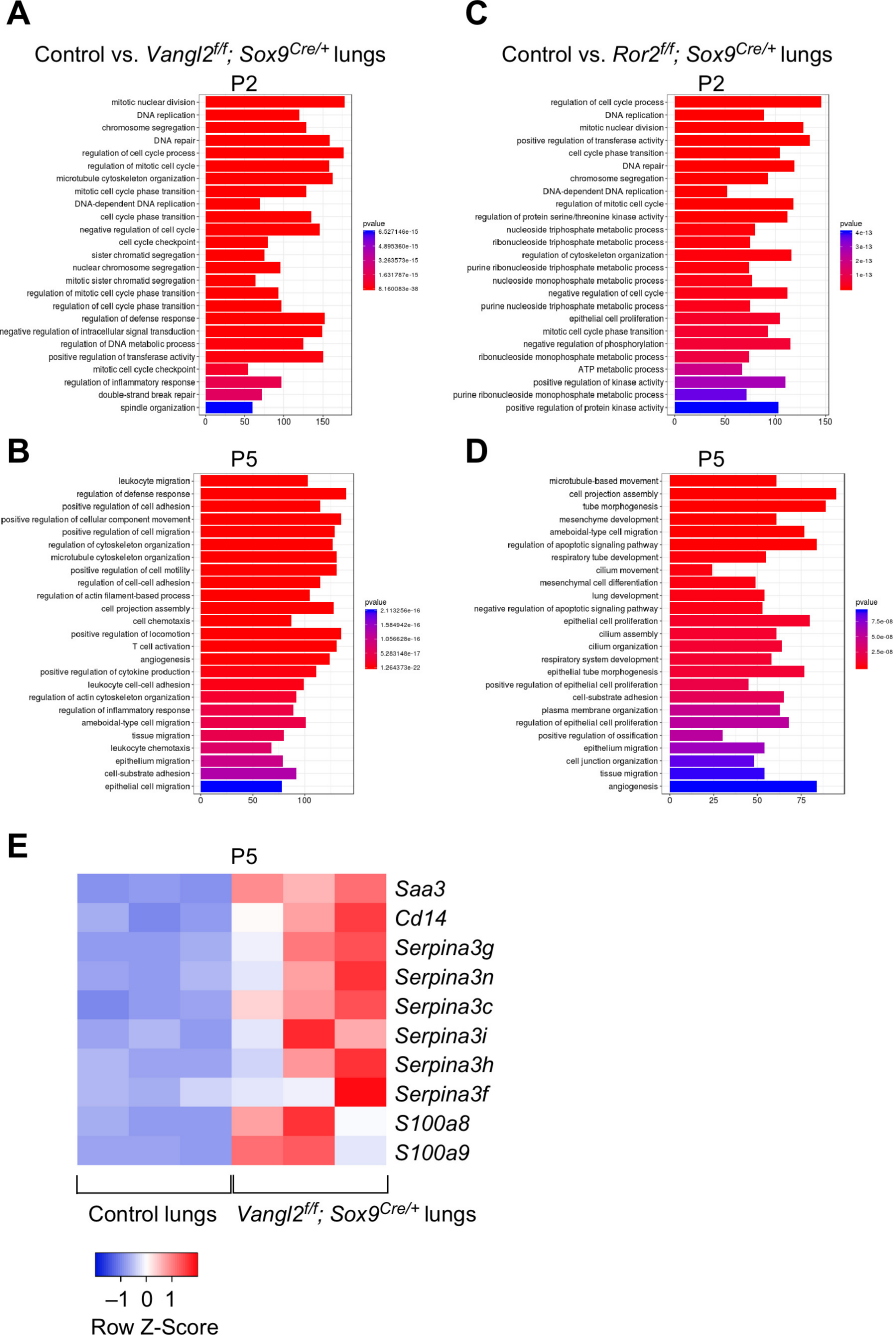
*Vangl2* and *Ror2* regulate similar pathways. (**A–D**) Pathway analysis of transcriptomes derived from RNA-Seq of control, *Vangl2^f/f^; Sox9^Cre/+^*, and *Ror2^f/f^; Sox9^Cre/+^* lungs at postnatal (P) day 2 and 5 (n = 3 for each group). The top 25 enriched terms in GO (gene ontology) biological processes were shown. Loss of *Vangl2* or *Ror2* revealed changes in similar pathways, suggesting that *Vangl2* and *Ror2* function in the same pathway. (**E**) Heatmap of selected mouse genes from control and *Vangl2^f/f^; Sox9^Cre/+^* lungs at P5. Loss of epithelial *Vangl2* in mouse lungs activated these genes. Interestingly, they are known to be elevated in lungs of human emphysema patients and are biomarkers for emphysema.

We performed bulk RNA-Seq analysis of control and *Vangl2^f/f^; Sox9^Cre/+^* lungs collected at P2 and P5. Our phenotypic analysis has revealed a major effect of epithelial PCP signaling on myofibroblasts and perhaps other lung cell types as well. While it would be ideal to use sorted lung cells for RNA-Seq, it would be difficult to sort myofibroblasts away from airway smooth muscle cells and vascular smooth muscle cells since they express a similar set of markers. As expected, *Pdgfra* expression was downregulated in *Vangl2^f/f^; Sox9^Cre/+^* lungs while *Pdgfa* expression was not reduced. Several other pathways that are perturbed were identified (Figure 4A and B, Figure 4—figure supplement 1). They include actomyosin cytoskeleton signaling, Wnt/β-catenin signaling, LXR/RXR signaling, CXCR4 signaling, CDK5 signaling, Toll-like receptor signaling and acute phase response signaling.

**Figure 4—figure supplement 1.**
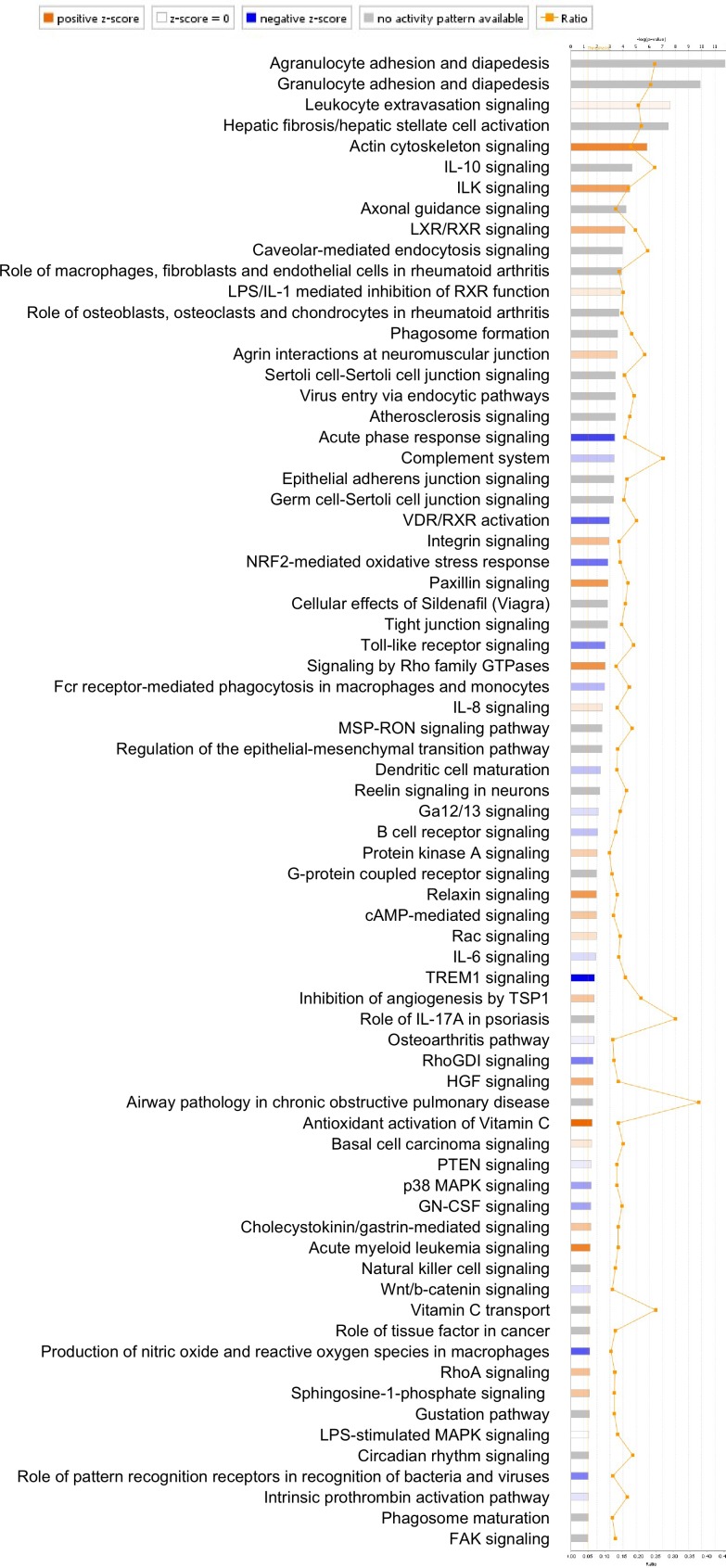
Pathway analysis of differentially expressed genes identified in RNA-Seq analysis of control and *Vangl2^f/f^; Sox9^Cre/+^* lungs at postnatal (P) day 5. p-value and z-score were shown. The calculated z-score indicates the prediction of overall increase or decrease in pathway activity. For a z-score >0, the pathway is expected to be activated; for a z-score <0, the pathway is expected to be inhibited. The ratio indicates the ratio of genes from the dataset that map to the pathway divided by the total number of genes that map to the same pathway. The orange line indicates a threshold of -log(p-value) =1.30 (p<0.05) and the cutoff was set at -log(p-value) =3 (p<0.001).

We also performed RNA-Seq analysis on control and *Ror2^f/f^; Sox9^Cre/+^* lungs collected at P2 and P5 (Figure 4C and D). We expect that many changes in transcriptional responses would follow a similar trend in lungs deficient in epithelial *Vangl2* or *Ror2*, if *Ror2* is responsible for mediating *Vangl2* activity. Indeed, loss of *Vangl2* and *Ror2* showed similar changes in many pathways, affirming the genetic studies in which they function in the same axis.

### *Vangl2*-deficient interstitial fibroblasts/myofibroblasts have disorganized actomyosin cytoskeletons and are defective in migration assays

**Figure 5.**
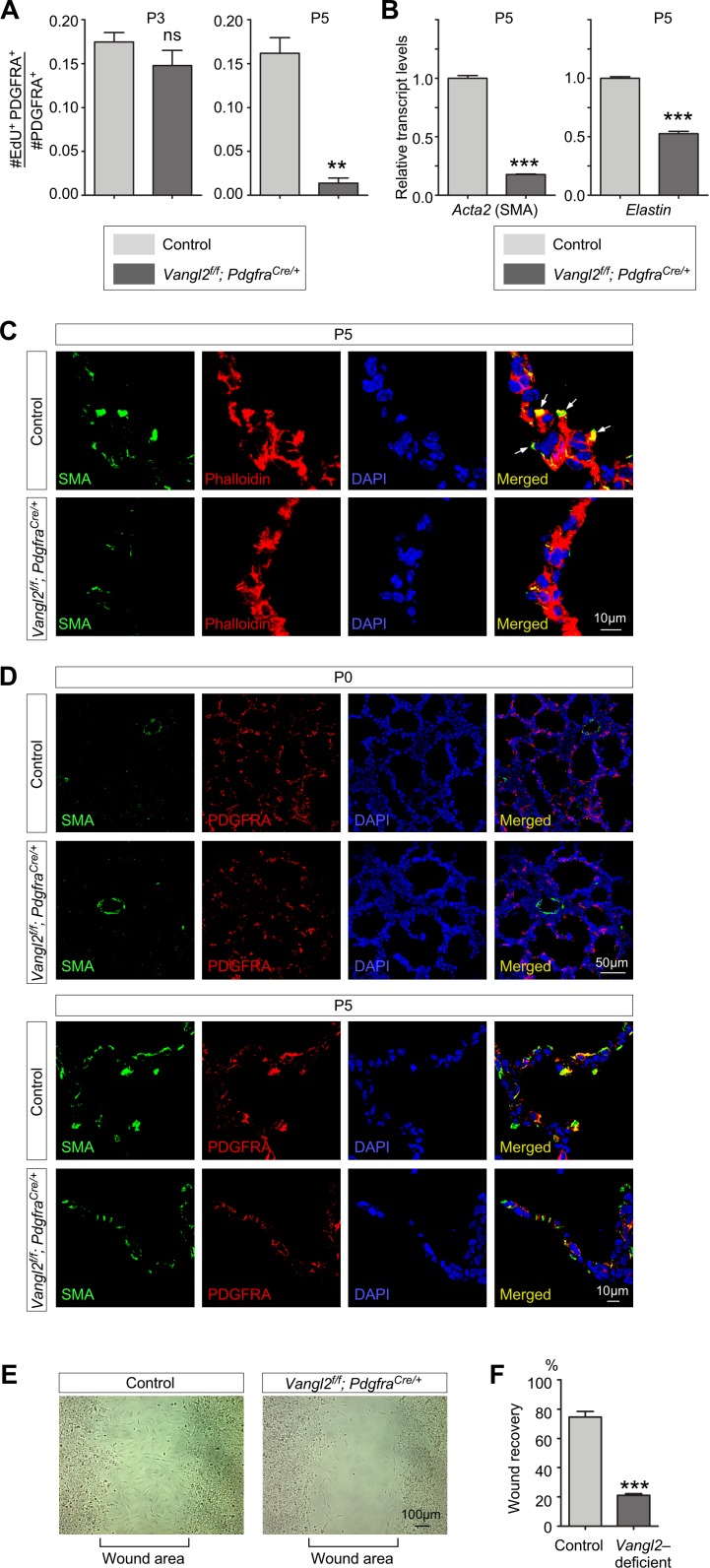
*Vangl2* regulates the cytoskeleton of myofibroblasts and their migration. (**A**) Quantification of myofibroblast proliferation in control and *Vangl2^f/f^; Pdgfra^Cre/+^* lungs at postnatal (P) day 3 and 5. The rate of myofibroblast proliferation was calculated as the ratio of the number of EdU^+^ myofibroblasts (EdU^+^PDGFRA^+^) to the number of myofibroblasts (PDGFRA^+^). An apparent reduction in the percentage of proliferating myofibroblasts was detected in *Vangl2^f/f^; Pdgfra^Cre/+^* lungs compared to controls (n = 3 for each group) at P5 but not at P3. (**B**) qPCR analysis of *Acta2* (*SMA*) and *Elastin* in control and *Vangl2^f/f^; Pdgfra^Cre/+^* lungs at P5. The mRNA levels of *Acta2* and *Elastin* were significantly reduced in the absence of *Vangl2* in myofibroblasts (n = 3 for each group). (**C**) Immunostaining of lung sections collected from control and *Vangl2^f/f^; Pdgfra^Cre/+^* mice at P5. The actomyosin cytoskeleton (stained by phalloidin) failed to organize around the prospective sites of secondary septa formation in *Vangl2^f/f^; Pdgfra^Cre/+^* lungs. In addition, smooth muscle actin (SMA) levels were significantly reduced and did not form stress fibers at the prospective sites of secondary septation. Arrows point to rudimentary secondary septa in control lungs. (**D**) Immunostaining of lung sections collected from control and *Vangl2^f/f^; Pdgfra^Cre/+^* mice at P0 and P5. Migration of myofibroblasts (PDGFRA^+^/SMA^+^) to the prospective sites of secondary septa failed to occur in the mutant lungs. (**E**) Wound recovery assays to assess the migratory ability of myofibroblasts derived from control and *Vangl2^f/f^; Pdgfra^Cre/+^* lungs. Within 36–48 hr, the wound area has been populated by migrating myofibroblasts derived from control lungs. By contrast, few myofibroblasts from *Vangl2^f/f^; Pdgfra^Cre/+^* lungs reached the wound area within the same time frame. (**F**) Quantification of wound recovery by myofibroblasts derived from control and *Vangl2^f/f^; Pdgfra^Cre/+^* lungs within 36–48 hr (n = 3 for each group). These results imply that mesenchymal *Vangl2* controls subsequent myofibroblast proliferation after the initial expansion or the migration defect exerts a secondary effect on myofibroblast proliferation or both. All values are mean ± SEM. (***) p<0.001 (unpaired Student’s *t*-test).

We found that myofibroblast proliferation was also diminished in *Vangl2^f/f^; Pdgfra^Cre/+^* lungs but only at later stages of postnatal development while the initial expansion of fibroblasts/myofibroblasts was unaffected (Figure 5A). This is in contrast to defective fibroblast/myofibroblast expansion in *Vangl2^f/f^; Sox9^Cre/+^* lungs (Figure 3C). These results suggest that the primary role of mesenchymal PCP signaling is to control myofibroblast migration and function while epithelial PCP signaling plays a key role in the initial expansion of fibroblasts/myofibroblasts. Both SMA and elastin production was significantly reduced in *Vangl2^f/f^; Pdgfra^Cre/+^* lungs (Figure 5B). It is expected that failure of myofibroblast migration to the prospective site of secondary septation deprived them of receiving the PDGF ligand for proliferation. It is interesting to note that myofibroblasts send out cellular extensions to form a network of myofibroblasts after expansion of the fibroblast/myofibroblast population (Figure 5—figure supplement 1). In this process, VANGL2 was found to colocalize with SMA on these cellular extensions, suggesting a critical role of PCP signaling in controlling motility of myofibroblasts (Figure 5—figure supplement 1).

**Figure 5—figure supplement 1.**
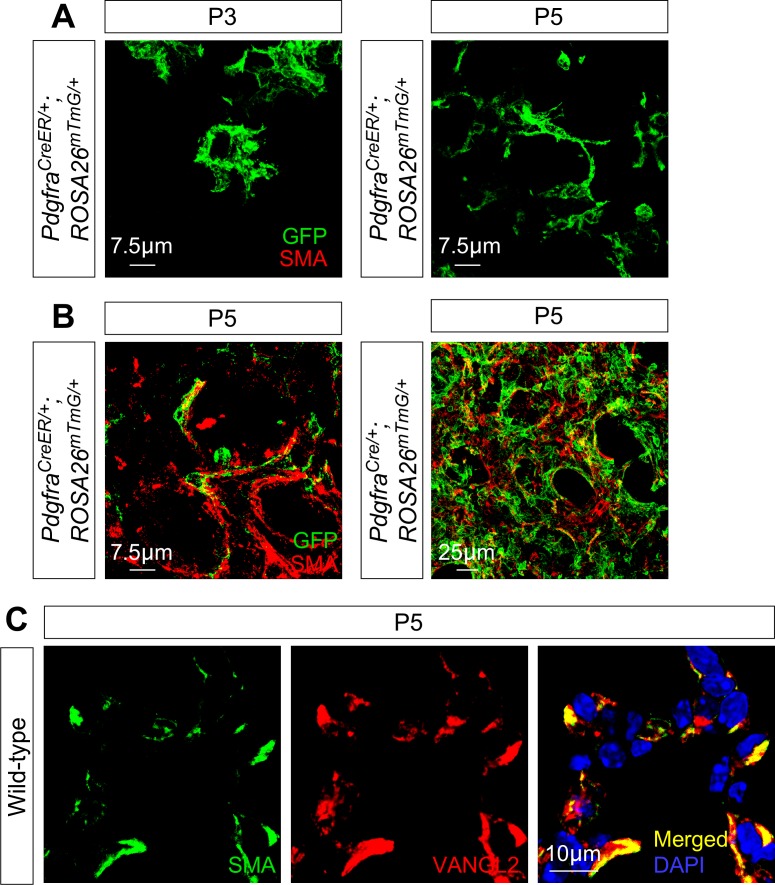
Myofibroblasts send out cellular extensions to form a network during alveologenesis. (**A**) Immunostaining of lung sections from *Pdgfra^CreER/+^; ROSA26^mTmG/+^* mice at postnatal (P) day 3 and 5. Leaky CreER expression labeled myofibroblasts. Cellular extensions of myofibroblasts became apparent at P5. (**B**) Immunostaining of lung sections from *Pdgfra^CreER/+^; ROSA26^mTmG/+^* or *Pdgfra^Cre/+^; ROSA26^mTmG/+^* mice at P5. Confocal stacks were shown to visualize the network of myofibroblasts. Smooth muscle actin (SMA) localized to cellular extensions of myofibroblasts. (**C**) Immunostaining of lung sections from wild-type mice at P5. VANGL2 and SMA colocalized at the cellular extensions of myofibroblasts.

We examined the actomyosin cytoskeleton in *Vangl2*-deficient myofibroblasts from *Vangl2^f/f^; Pdgfra^Cre/+^* lungs in comparison with control myofibroblasts. Phalloidin staining of F-actin (filamentous) revealed a disorganized actomyosin cytoskeleton and smooth muscle actin failed to produce organized stress fibers in mutant myofibroblasts (Figure 5C). We also analyzed fibroblast/myofibroblast distribution in *Vangl2^f/f^; Pdgfra^Cre/+^* lungs at P0 and P5 (Figure 5D). We found no difference in the distribution of PDGFRA^+^ fibroblasts at P0. However, at P5 while PDGFRA^+^/SMA^+^ myofibroblasts had migrated to the prospective sites of secondary septa in control lungs, PDGFRA^+^/SMA^+^ cells in the mutant lungs stayed in the primary septa.

These results predict that migration of *Vangl2*-deficient myofibroblasts would be compromised. To test this idea, we isolated myofibroblasts from control and *Vangl2^f/f^; Pdgfra^Cre/+^* lungs and seeded them onto the migration chamber. The rate of myofibroblast migration into the cell-free area was measured. While control myofibroblasts occupied the cell-free area after 36–48 hr, only scant *Vangl2*-deficient myofibroblasts were detected in the cell-free area during this time period (Figure 5E and F). This indicates that the mobility of myofibroblasts was compromised due to loss of PCP signaling in these cells. Reduced myofibroblast proliferation and migration thus underlies defective alveolar development in *Vangl2^f/f^; Pdgfra^Cre/+^* mice.

### Alveolar type I cells devoid of VANGL2 are associated with disrupted cytoskeletons and fail to encase myofibroblasts necessary for secondary septa formation

**Figure 6.**
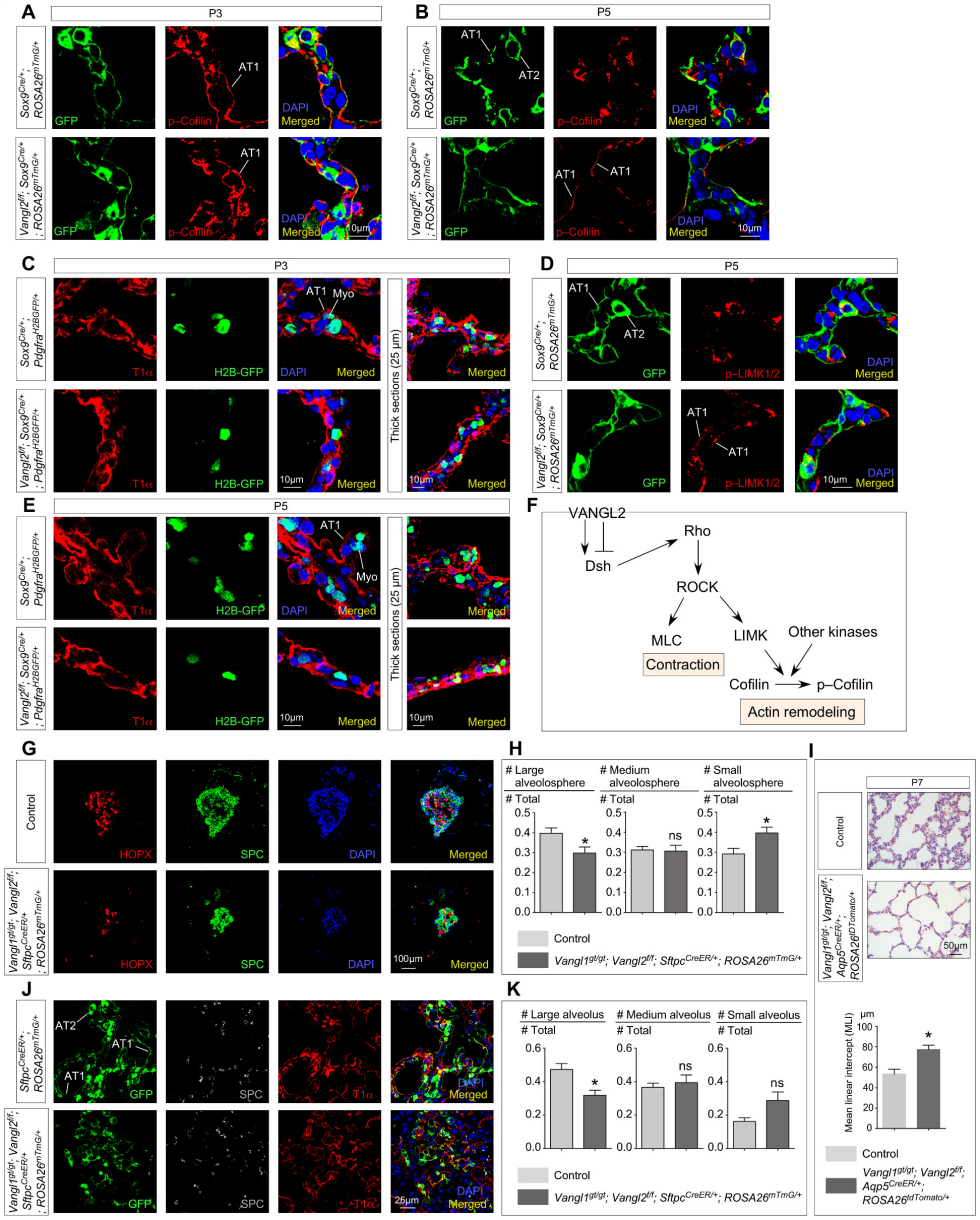
*Vangl2* controls the cytoskeleton of alveolar type I cells, their cell shape changes and their ability in forming alveolospheres and new alveoli. (**A, B, D**) Immunostaining of lung sections collected from *Sox9^Cre/+^; ROSA26^mTmG/+^* (control) and *Vangl2^f/f^; Sox9^Cre/+^; ROSA26^mTmG/+^* mice at postnatal (P) day 3 and 5. GFP from the *ROSA26* locus (*ROSA26^mTmG^*) was activated in both alveolar type I (AT1) and type II (AT2) cells which could be distinguished by their morphology. The levels of phosphorylated (p) Cofilin and LIMK were significantly reduced in AT1 cells in control lungs by P5. By contrast, p-Cofilin and p-LIMK persisted in AT1 cells in *Vangl2^f/f^; Sox9^Cre/+^; ROSA26^mTmG/+^* lungs. (**C, E**) Immunostaining of lung sections collected from *Sox9^Cre/+^; Pdgfra^H2BGFP/+^* (control) and *Vangl2^f/f^; Sox9^Cre/+^; Pdgfra^H2BGFP/+^* mice at P3 and P5. Cell shape change of AT1 cells (T1α^+^) was observed in control lungs at the prospective sites of secondary septation to encase myofibroblasts (H2BGFP^+^) (myo) that had migrated toward AT1 cells. AT1 cells in *Vangl2^f/f^; Sox9^Cre/+^; Pdgfra^H2BGFP/+^* lungs failed to undergo similar morphological changes. (**F**) Schematic diagram of a *Vangl2*-initiated signaling cascade that leads to phosphorylation of Cofilin and actin remodeling. (**G**) Immunostaining of alveolospheres derived from control and *Vangl1^gt/gt^; Vangl2^f/f^; Sftpc^CreER/+^; ROSA26^mTmG/+^* mice injected with tamoxifen. Both SPC^+^ and HOPX^+^ cells were found in alveolospheres derived from control and *Vangl1^gt/gt^; Vangl2^f/f^; Sftpc^CreER/+^; ROSA26^mTmG/+^* mice despite a difference in size and organization. (**H**) Quantification of the percentage of large (cross-sectional area >0.06mm^2^), medium (cross-sectional area between 0.015mm^2^ and 0.06mm^2^) and small (cross-sectional area <0.015mm^2^) alveolospheres derived from control and *Vangl1^gt/gt^; Vangl2^f/f^; Sftpc^CreER/+^; ROSA26^mTmG/+^* mice (n = 5 for each group). The percentage of large alveolospheres derived from *Vangl1^gt/gt^; Vangl2^f/f^; Sftpc^CreER/+^; ROSA26^mTmG/+^* mice was reduced. (**I**) Hematoxylin and eosin-stained lung sections of wild-type and *Vangl1^gt/gt^; Vangl2^f/f^; Aqp5^CreER/+^; ROSA26^tdTomato/+^* mice injected with tamoxifen and collected at P7. Enlarged saccules were found in *Vangl1^gt/gt^; Vangl2^f/f^; Aqp5^CreER/+^; ROSA26^tdTomato/+^* lungs with an increased MLI in comparison with controls (n = 3 for each group). (**J**) Immunostaining of lung sections collected from *Sftpc^CreER/+^; ROSA26^mTmG/+^* (control) and *Vangl1^gt/gt^; Vangl2^f/f^; Sftpc^CreER/+^; ROSA26^mTmG/+^* mice injected with tamoxifen and treated with bleomycin. Lungs were harvested at 30 days post-bleomycin administration. In control lungs, GFP-labeled AT2 cells proliferated and differentiated into AT1 cells to form new alveoli. By contrast, fewer alveoli were produced in *Vangl1^gt/gt^; Vangl2^f/f^; Sftpc^CreER/+^; ROSA26^mTmG/+^* lungs. (**K**) Quantification of the percentage of large (cross-sectional area >0.6mm^2^), medium (cross-sectional area between 0.2mm^2^ and 0.6mm^2^) and small (cross-sectional area <0.2mm^2^) alveoli derived from *Sftpc^CreER/+^; ROSA26^mTmG/+^* (control) and *Vangl1^gt/gt^; Vangl2^f/f^; Sftpc^CreER/+^; ROSA26^mTmG/+^* mice (n = 5 for each group). The percentage of large alveoli in *Vangl1^gt/gt^; Vangl2^f/f^; Sftpc^CreER/+^; ROSA26^mTmG/+^* lungs was reduced. All values are mean ± SEM. (*) p<0.05; (**) p<0.01; (***) p<0.001; ns, not significant (unpaired Student’s *t*-test).

Abnormal secondary septation in *Vangl2^f/f^; Sox9^Cre/+^* lungs due to loss of epithelial *Vangl2* suggests that the primary defect originates from faulty alveolar epithelial cells. This is in contrast to the traditional view of secondary septa formation in which the main driving force of septa formation is derived from myofibroblast migration and elastin deposition by myofibroblasts (Chao et al., 2016). Having established a critical role of alveolar type I and type II cells in PDGF secretion during alveologenesis, we assessed the function of *Vangl2* in alveolar type I cells during alveolar formation. We conjectured that abrogated PCP signaling in alveolar type I cells would perturb the actomyosin cytoskeleton and impair their ability to adopt the cell shape necessary for encasing myofibroblasts during secondary septation. To validate this model, we initially used phalloidin to label F-actin and examined the actomyosin cytoskeleton in alveolar type I cells of control and *Vangl2^f/f^; Sox9^Cre/+^* lungs. Due to the extended morphology of alveolar type I cells, it was very difficult to obtain a clear view of the actomyosin cytoskeleton. We then searched for other markers for the actomyosin cytoskeleton and found that phosphorylated Cofilin and LIM-kinase (LIMK) were significantly increased in *Vangl2*-deficient alveolar type I cells, compared to controls (Figure 6A, B and D). Phosphorylation of Cofilin by LIMK inhibited its ability to depolymerize actin, leading to a failure in actin bundle remodeling (Figure 6F). This would generate disorganized stress fibers in *Vangl2*-deficient AT1 cells.

**Figure 6—figure supplement 1.**
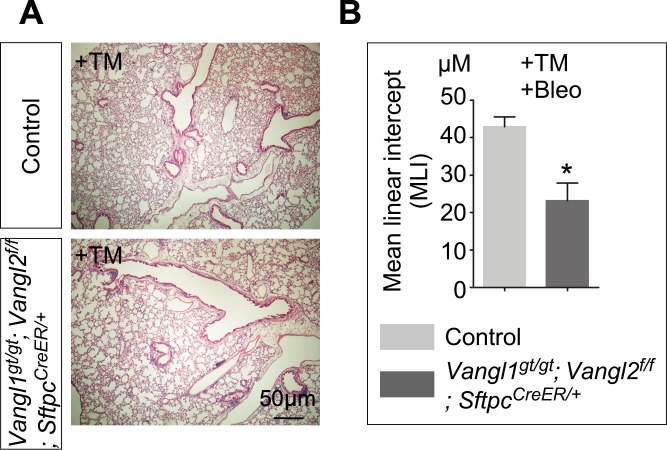
Bleomycin-induced lung injury results in a reduced MLI in the absence of *Vangl1/2.* (**A**) Hematoxylin and eosin-stained lung sections of control and *Vangl1^gt/gt^; Vangl2^f/f^; Sftpc^CreER/+^* mice, which received tamoxifen (TM) injection. Lungs collected at 3 months post-TM (without bleomycin treatment) showed no evidence of lung fibrosis. (**B**) Measurement of the mean linear intercept (MLI) in control and *Vangl1^gt/gt^; Vangl2^f/f^; Sftpc^CreER/+^* mice, which received TM and subsequently bleomycin. Lungs collected at one month post-bleomycin showed a reduction in the MLI of the mutant lungs. The reduced MLI in *Vangl1^gt/gt^; Vangl2^f/f^; Sftpc^CreER/+^* lungs indicates a reduced distance between two primary or secondary septa in the regenerating alveoli, consistent with the definition of smaller alveoli. Smaller alveoli may be related to alterations in the cytoskeleton in the absence of *Vangl1/2*. All values are mean SEM. (*) p<0.05 (unpaired Student’s *t*-test).

Disruption of the actomyosin cytoskeleton in alveolar type I cells in the absence of PCP signaling raised the possibility that AT1 cells failed to undergo morphogenetic adjustment to sheathe myofibroblasts and capillaries during secondary septa formation. Indeed, immunohistochemical analysis revealed slender *Vangl2*-deficient AT1 cells that failed to fold up to encase myofibroblasts (Figure 6C and E). This observation was further validated by TEM studies of control and *Vangl2^f/f^; Sox9^Cre/+^* lungs. For instance, AT1 cells in control lungs at P3 displayed folding to sheathe myofibroblasts that had migrated to the prospective sites of secondary septa formation (Figure 1C). By contrast, such coordinated morphological changes in AT1 cells did not occur in the mutant lungs (Figure 1C). These results imply an active role of AT1 cells in secondary septation likely through cell shape change, which would complement the action of myofibroblast migration and contraction in this highly coordinated process.

Taken together, we conclude that PCP signaling operates in both the lung epithelium and mesenchyme and control distinct aspects of cellular properties in each compartment necessary for alveologenesis. This includes ligand secretion, cell shape change and migration.

### Alveolar type I cells lacking VANGL1/2 are compromised in alveolosphere formation

To further investigate the role of AT1 cells in alveolar formation, we adopted the alveolosphere assay (Barkauskas et al., 2013) and examined the cellular behavior of control and *Vangl1/2*-deficient alveolar cells in spheroid formation. This organoid system retains many important aspects of alveolar formation and would reveal the intrinsic cellular defects in AT1 cells during alveologenesis (Barkauskas et al., 2017). In this assay, fibroblasts/myofibroblasts are in direct contact with AT1 cells and obviate the need of PDGF-mediated migration.

We isolated alveolar type II cells (GFP^+^) from *Sftpc^CreER/+^; ROSA26^mTmG/+^* mice (Lin et al., 2012; Muzumdar et al., 2007) by fluorescence-activated cell sorting (FACS). Tamoxifen was injected into *Sftpc^CreER/+^; ROSA26^mTmG/+^* mice to activate Sftpc^CreER^ and label SPC^+^ AT2 cells with GFP. Sorted alveolar type II cells were mixed with fibroblasts and cultured in 3D. In this process, AT2 (SPC^+^) cells proliferated and differentiated into AT1 (HOPX^+^) cells. After two weeks, lung organoids formed, in which alveolus-like structures expressing markers for alveolar type I cells were produced from alveolar type II cells in the presence of fibroblasts (Figure 6G and H). We performed a similar experiment using *Vangl1/2*-deficient AT2 cells purified from *Vangl1^gt/gt^; Vangl2^f/f^; Sftpc^CreER/+^; ROSA26^mTmG/+^* mice that had been administered with tamoxifen to delete *Vangl2* in SPC^+^ AT2 cells and in all AT1 cells originated from AT2 cells. In the absence of *Vangl1/2*, differentiation of AT2 cells and proliferation of AT2 and AT1 cells were unaffected. However, although a similar number of alveolospheres was obtained, the size distribution of the alveolospheres derived from *Vangl1/2*-deficient AT1 cells was dominated by those with a smaller size (Figure 6G and H). In control spheres, HOPX^+^ AT1 cells are located inside the spheres and are surrounded by SPC^+^ AT2 cells. This organization was not observed in spheres, in particular, medium and small alveolospheres, derived from *Vangl1/2*-deficient cells. This is consistent with defective cellular properties in *Vangl1/2*-deficient AT1 cells required for morphogenesis.

To assess the role of AT1 cells in alveologenesis, we generated *Vangl1^gt/gt^; Vangl2^f/f^; Aqp5^CreER/+^* mice and administered tamoxifen to these animals at P2. Activation of CreER by tamoxifen in Aquaporin 5 (AQP5)^+^ cells induced *Vangl2* removal in AT1 cells. Analysis of lungs at P7 revealed areas of defective alveolar development due to loss of *Vangl1/2* (Figure 6I). This suggests a vital function of *Vangl1/2* in alveolar type I cells during alveologenesis. Together, our results support a central role of *Vangl1/2* in controlling the cellular behavior of AT1 cells during alveolar formation.

### Loss of *Vangl1/2* in alveolar type I cells impairs their ability to form new alveoli following lung injury

To further assess the function of *Vangl1/2* in AT1 cells during alveolar formation, we took advantage of the observation that bleomycin applied to adult mouse lungs induces formation of new alveoli (Walters and Kleeberger, 2008; Mouratis and Aidinis, 2011; Egger et al., 2013). In this setting, in response to bleomycin-induced loss of alveoli and fibrosis, alveolar type II cells produce alveolar type I cells, which contribute to new alveoli and lung repair in mice. This feature provided an ideal setup for testing how loss of *Vangl1/2* in AT1 cells affects their ability in alveolar formation. To this end, we generated *Sftpc^CreER/+^; ROSA26^mTmG/+^* (control) and *Vangl1^gt/gt^; Vangl2^f/f^; Sftpc^CreER/+^; ROSA26^mTmG/+^* adult mice and injected them with tamoxifen to inactivate *Vangl2* in alveolar type II cells, which were also labeled by GFP (from the *ROSA26^mTmG^* locus). Alveolar type I cells produced from alveolar type II cells would also be lineage-labeled by GFP. We then subjected these animals to bleomycin following the standard protocol. After 4 weeks post-bleomycin administration, lung repair was extensive in control lungs and newly formed alveoli had replaced the damaged lung tissues (Figure 6J and K). By contrast, fewer GFP^+^ alveoli in *Vangl1^gt/gt^; Vangl2^f/f^; Sftpc^CreER/+^; ROSA26^mTmG/+^* mice were found and they failed to reach the same size as that in control lungs (Figure 6J and K, Figure 6—figure supplement 1). This suggests that *Vangl1/2*-deficient AT1 cells were compromised in their ability to form new alveoli although *Vangl1/2*-deficient AT2 cells could also contribute to the observed defects in alveolar repair. While additional studies are required, these findings suggest a critical role of *Vangl1/2* in controlling the cellular behavior of alveolar type I (and/or type II) cells during alveolar formation.

### COPD patients have reduced levels of *WNT5A* and *VANGL2* expression

**Figure 7.**
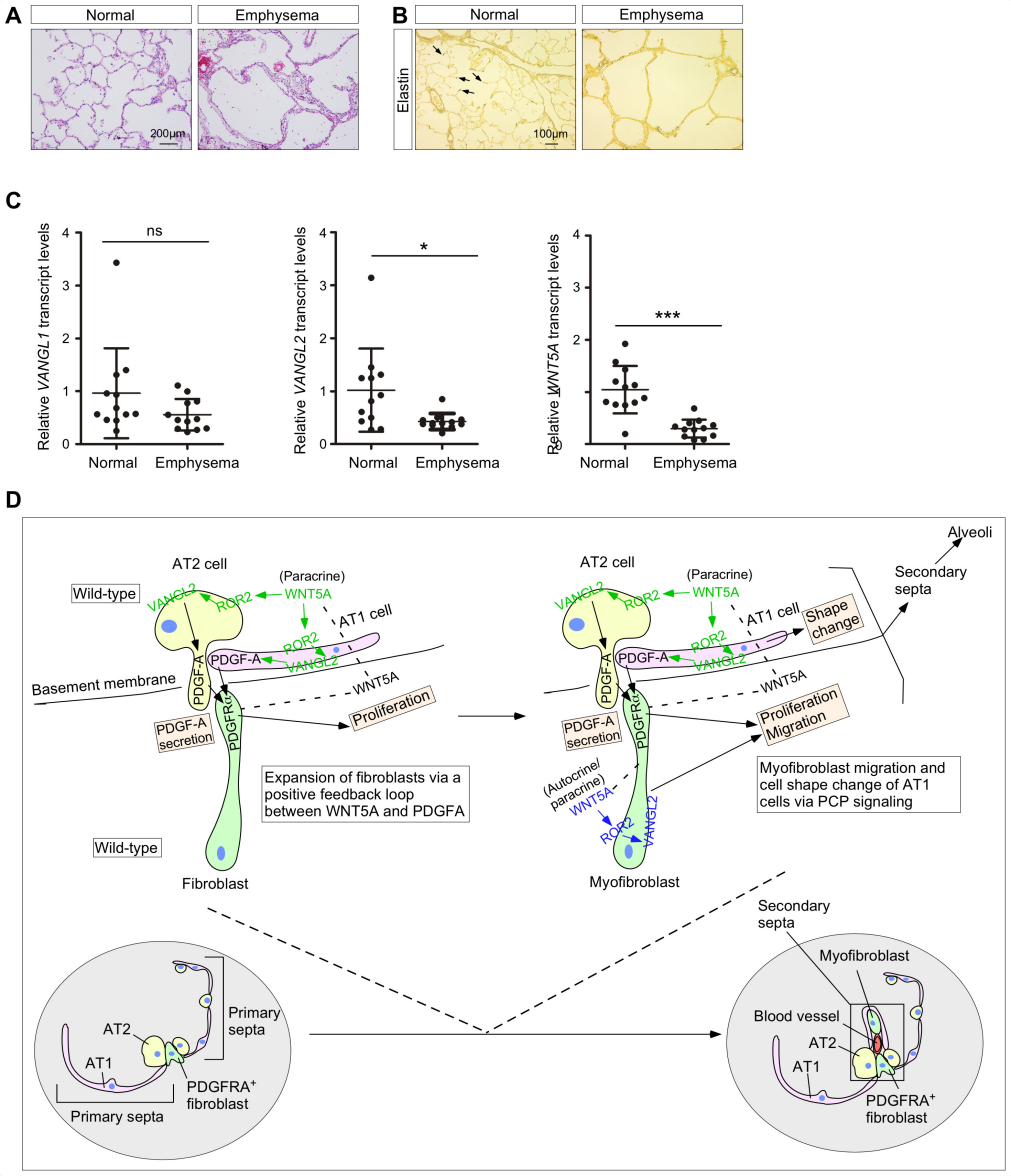
The WNT–VANGL axis is downregulated in the lungs of human emphysema patients. (**A**) Hematoxylin and eosin-stained lung sections of normal and emphysema patients. Characteristic disruption of alveoli was observed in emphysema patients, resulting in increased airspace. (**B**) Elastin staining of lung sections of normal and emphysema patients. The slides were counterstained with tartrazine. Elastin, which was detected at the secondary septa in normal lungs (arrows), was greatly reduced in the lungs of emphysema patients. (**C**) qPCR analysis of *VANGL1, VANGL2 and WNT5A* in lungs from normal and emphysema patients (n = 12 for each group). The mRNA levels of *VANGL2* and *WNT5A* were significantly reduced in emphysema patients. All values are mean ± SEM. (*) p<0.05; (**) p<0.01; (***) p<0.001; ns, not significant (unpaired Student’s *t*-test). (**D**) A new model of alveolar formation through control of cellular properties by PCP signaling. We propose that epithelial PCP signaling via the *Wnt5a–Ror2–Vangl2* axis (colored green) controls PDGF secretion from alveolar type I and type II cells to promote proliferation of mesenchymal fibroblasts. In this process, a positive feedback loop between WNT5A and PDGF leads to expansion of the fibroblast pool required for subsequent alveologenesis. The expanded fibroblast population expresses SMA and becomes myofibroblasts, which continue to proliferate. In addition, myofibroblasts migrate to the prospective site of secondary septation in response to PCP signaling (colored blue). Likewise, epithelial PCP signaling (colored green) instructs cell shape changes of alveolar type I cells necessary for encasing myofibroblasts that migrate toward the site of secondary septation. All of these cellular events (orange-colored boxes) are due to modulation of the actomyosin cytoskeleton via the *Wnt5a–Ror2–Vangl2* axis.

To explore whether studies of PCP signaling in alveolar formation in mice recapitulate human diseases, we assessed PCP signaling in lung tissues of COPD/emphysema patients (Figure 7A and B). We found that the expression levels of *WNT5A* and *VANGL2* were reduced (Figure 7C), suggesting a link between PCP signaling and alveolar loss/regeneration in these patients. This finding is consistent with other studies in the literature, in which *VANGL2* expression was reported to be downregulated in a small cohort of COPD patient lungs (Poobalasingam et al., 2017). In addition, polymorphisms of human *VANGL2* have been associated with negative smoking impacts on lung function in humans (Poobalasingam et al., 2017).

It is interesting to note that SERPINA3 (a protease inhibitor), SAA3 (serum amyloid A) and CD14, whose expression was increased in *Vangl2*-deficient murine lungs (Figure 4E), are activated in COPD patients and are biomarkers for COPD (Bozinovski et al., 2008; Wood et al., 2009; von Scheele et al., 2011). This supports the idea that the PCP pathway is compromised in COPD patients and may contribute to their inability to form new alveoli.

Taken together, our studies have provided new mechanistic insight into alveolar formation and PCP signaling (Figure 7D). They also form the basis for future work to understand how different signaling pathways are integrated during alveologenesis in development and following injury.

## Discussion

Our studies have addressed a central question in alveolar development, the molecular basis of secondary septa formation (Figure 7D). Through genetic and molecular approaches, we decipher PCP signaling through a *Wnt5a–Ror2–Vangl2* axis as a key element in mediating cell-cell interactions between alveolar cells (type I and type II) and myofibroblasts. The PCP pathway controls PDGF signaling from alveolar type I and II cells to fibroblasts/myofibroblasts to promote their proliferation. This essential step ensures the production an adequate number of fibroblasts/myofibroblasts required for alveologenesis. A positive feedback loop between WNT5A and PDGF facilitates expansion of fibroblasts/myofibroblasts. Subsequently, orchestrated movement between alveolar type I cells and myofibroblasts is regulated by PCP signaling, including cell shape change of alveolar type I cells and migration of myofibroblasts. As a result, they come in close proximity to each other and undergo coordinated morphogenesis to produce secondary septa and alveoli. In this process, myofibroblasts also send out cellular processes to form a network that likely functionally connects alveoli. These critical events require changes in cellular properties mediated by PCP signaling and reflect a novel facet of PCP function. We speculate that this new insight can serve as a paradigm for understanding PCP function in other tissues beyond the classical tissue polarity. Moreover, our findings suggest that the developmental programs executed by PCP are utilized for tissue repair in the lung. This establishes the foundation for elucidating disease mechanisms of BPD and COPD caused by alveolar loss.

Genetic and molecular analyses of *Wnt5a*, *Ror2* and *Vangl2*-deficient lungs demonstrate a critical role of these PCP components in controlling the actomyosin cytoskeleton, which underlies ligand secretion/delivery, and cell migration and interaction during secondary septa formation. Surprisingly, these processes do not appear to involve the classical tissue polarity, in which asymmetric distribution of PCP components is associated with polarization of cells (Ossipova et al., 2015). Instead, the primary function of the *Wnt5a–Ror2–Vangl2* axis is to modulate the cellular properties of lung cells required for alveolar formation. Thus, while the PCP pathway functions in many cellular processes that rely on the actomyosin cytoskeleton, our studies revealed that PCP activity is not confined to tissue polarity. Our work shows that PCP signaling exerts its effects at the subcellular levels via controlling the cytoskeleton in alveolar type I, type II cells and myofibroblasts. This notion is consistent with studies that reported a purported function of PCP in axonal guidance (Zou, 2012) or postsynaptic compartmentalization (Nagaoka and Kishi, 2016), in which PCP was proposed to also operate at the subcellular level. Moreover, our findings suggest that the PCP pathway controls important cellular processes beyond epithelial layers as demonstrated by the involvement of PCP signaling in myofibroblast migration. Hence, our investigation establishes novel modes of PCP function in tissue patterning. We anticipate that additional cellular processes that utilize similar modes of PCP signaling as reported here will be uncovered in other tissues.

Loss of *Ror2* in either lung epithelium or mesenchyme recapitulates phenotypes due to lack of *Vangl2*. This supports a crucial role of ROR2 in activating VANGL2 and downstream events. However, we cannot rule out the possibility that a Fz receptor (Wang et al., 2016) is also involved in relaying the WNT5A signal to VANGL2. In this case, Fz and ROR2 would cooperate in a non-redundant manner to transduce the WNT5A signal. Identifying the putative Fz receptor and elucidating its functions would significantly increase our understanding of the signaling cascade that regulates secondary septa formation.

Our genetic and molecular studies support a new model in which morphological changes of AT1 cells induced by PCP signaling are required for secondary septa elongation. An active role of AT1 cells in secondary septation contrasts their passive role implied from the existing models in which myofibroblasts drive secondary septa formation. Nevertheless, further studies are required to define the functional consequence of PCP signaling in AT1 cells and delineate the relative contributions of AT1 and AT2 cells to alveologenesis. We also showed that morphological changes in myofibroblasts are also critical for secondary septa formation. Thus, secondary septa development relies on coordinated cell shape changes in both alveolar epithelial cells and myofibroblasts. A key element in this process is an altered actomyosin cytoskeleton controlled by the *Wnt5a–Ror2–Vangl2* axis. How this axis is integrated with other regulators of the cytoskeleton requires further investigations. We also do not have the cellular resolution to discern the exact sequence of events. This would rely on improved microscopy and the ability to image lung slices for an extended period of time. New culture conditions are also needed to recapitulate alveolar formation in ex vivo lung explants or lung slices. Future development of fluorescent probes that enable monitoring lung development in vivo would be an important step forward to validate these models.

In this study, we have focused on AT1/2 cells and myofibroblasts in secondary septa formation. A third component of the secondary septa is endothelial cells. It is unclear whether PCP signaling controls capillary morphogenesis during alveolar development. This would require additional genetic and molecular studies, similar to those described in this work. Interestingly, PCP signaling has been implicated in network organization of vessels in other organs (Sewduth and Santoro, 2016).

Our analysis of *Vangl2*-deficient lungs revealed that PDGF ligands produced in alveolar cells fail to signal to myofibroblasts. Our data point to defective delivery of the PDGF ligand due to disrupted cytoskeleton in the absence of PCP signaling. Additional cell biological and biochemical studies are required to pinpoint the defects in PDGFA trafficking in alveolar type I and type II cells. We propose that PDGF signal reception is compromised by loss of cell-cell contacts between alveolar cells and mesenchymal myofibroblasts. Further validation of this model would require direct visualization of ligand–receptor interactions on juxtaposed membranes between ligand-producing and –receiving cells, for instance, using split GFP technology (Feinberg et al., 2008) where GFP signal is reconstituted at sites of ligand–receptor contact. An alternative possibility of cell-cell contacts between alveolar cells and mesenchymal myofibroblasts involves actin-based filopodia. This is similar to the cytonemes described in other systems including the mouse limb (Sanders et al., 2013). The function of cytonemes has been debated but recent data show that they are required for mediating ligand-receptor signaling for major signaling pathways in lieu of diffusion (Roy et al., 2014; Huang and Kornberg, 2015). Cytonemes originate from ligand-producing and receptor-producing cells, but cytonemes have not been reported in lung cells likely due to technical issues.

In conclusion, our work has provided key new insights into the cellular and molecular basis of secondary septation and alveolar formation. Moreover, we have revealed a novel mode of PCP signaling beyond tissue polarity. These studies set the stage for further investigation to obtain a complete mechanistic understanding of alveolar development and repair.

## Materials and methods

### Animal husbandry

Mouse strains used in this study are listed in the Key Resources table (Supplementary file 1). Matings were set up to obtain mice with the indicated genotypes described in this study. This study was performed in strict accordance with the recommendations in the Guide for the Care and Use of Laboratory Animals of the National Institutes of Health. The Institutional Animal Care and Use Committee (IACUC) at the University of California, San Francisco, approved all experiments performed in this study (protocol #AN173680-03).

### Generation of *Aqp5^CreER^* mice

To produce the *Aqp5^CreER^* mouse line, the translational start ATG in the mouse *Aqp5* genomic locus was replaced with a CreER-FRT-PGK-Neo-FRT cassette though gene targeting in E14 embryonic (ES) cells. The FRT-PGK-Neo-FRT cassette was subsequently removed by crosses with *FLPe* mice (Rodríguez et al., 2000).

### Histology and immunohistochemistry

Mouse lungs were dissected at the indicated time points and fixed in 4% paraformaldehyde (PFA) in PBS on ice for 1 hr. Tissues were embedded in paraffin wax or OCT (frozen sections) and sectioned at 7 μm. Thick sections were prepared in a similar manner. Histological analysis, such as hematoxylin and eosin (H&E) staining, was performed as described (Lin et al., 2017).

Elastin fiber staining was performed following Hart's staining method with minor modifications. Lung sections were deparaffinized with xylene, rehydrated with ethanol and water, and then placed in the working solution (10% Weigert's iron resorcin-fuchsin stock solution, 2% HCl and 70% ethanol) overnight at room temperature. After three washes in water, the sections were counterstained in 0.5% tartrazine/0.5% acetic acid for 3 min. Sections were rinsed three times in water and dehydrated with ethanol and xylene. The elastin fibers were stained black.

Immunohistochemistry was performed following standard procedures (Lin et al., 2017). Antibodies used in this study are listed in the Key Resources table (Supplementary file 1). The following primary antibodies were used for paraffin wax sections: rabbit anti-NKX2.1 (1:100, Epitomics), chicken anti-GFP (1:200, abcam), goat anti-CC10 (1:200, Santa Cruz Biotechnology), rabbit anti-prosurfactant protein C (proSP-C) (1:200, MilliporeSigma), hamster anti-T1α (1:200, Developmental Studies Hybridoma Bank), mouse anti-HOPX (1:100, Santa Cruz Biotechnology), and rabbit anti-phospho-AKT (Ser473) (1:150, Cell Signaling Technology). The following primary antibodies were used for frozen sections: rat anti-E-cadherin (1:200, Invitrogen), chicken anti-GFP (1:300, abcam), mouse anti-ACTA2 (1:200, Thermo Scientific Lab Vision), rat anti-PECAM-1 (CD31) (1:150, Santa Cruz Biotechnology), rabbit anti-PDGFRA (1:150, Cell Signaling Technology), rabbit anti-phospho-PDGFRA (Tyr754) (1:100, Cell Signaling Technology), rabbit anti-Cofilin (1:150, Cell Signaling Technology), rabbit anti-phospho-Cofilin (Ser3) (1:100, Cell Signaling Technology), rabbit anti-phospho-LIMK1 (Thr508)/LIMK2 (Thr505) (1:100, Cell Signaling Technology), and rat anti-VANGL2 (1:150, MilliporeSigma). Secondary antibodies and conjugates used were donkey anti-rabbit Alexa Fluor 488 or 594 (1:1000, Life Technologies), donkey anti-goat Alexa Fluor 488, donkey anti-chicken Alexa Fluor 488 or 647 (1:1000, Life Technologies), donkey anti-mouse Alexa Fluor 488 or 594 (1:1000, Life Technologies), and donkey anti-rat Alexa Fluor 594 (1:1000, Life Technologies). For biotinylated secondary antibodies, goat anti-hamster (1:1000, Jackson ImmunoResearch Laboratories), donkey anti-rabbit (1:1000, Jackson ImmunoResearch Laboratories), donkey anti-rat (1:1000, Jackson ImmunoResearch Laboratories) and horse anti-mouse (1:1000, Jackson ImmunoResearch Laboratories) were used. The signal was detected using streptavidin-conjugated Alexa Fluor 488, 594, or 647 (1:1000, Life Technologies). For antibodies against PDGFRA, phospho-PDGFRA (Tyr754), phospho-Cofilin (Ser3), phospho-AKT, phospho-LIMK1 (Thr508)/LIMK2 (Thr505) and VANGL2, instead of using streptavidin-conjugated Alexa Fluor 488, 594, or 647 as the tertiary antibody, HRP-conjugated streptavidin (1:1000, Perkin-Elmer) was used in combination with fluorogenic substrate Alexa Fluor 594 tyramide for 30 s (1:200, TSA kit; Perkin Elmer). F-actin was stained with rhodamine-conjugated phalloidin (1:200; Sigma).

For determining VANGL2 localization and distribution in the lung, frozen sections (at 20 and 50 μm) were prepared from the lungs of *Sox9^Cre/+^; ROSA26^mTmG/+^* and *Pdgfra^CreER/+^; ROSA26^mTmG/+^* mice. Sections were incubated with chicken anti-GFP and rat anti-VANGL2 at 4°C overnight. Donkey anti-chicken Alexa Fluor 488 and biotinylated donkey anti-rat secondary antibodies were incubated at 4°C overnight. Streptavidin-conjugated HRP tertiary antibody was incubated at room temperature for 2 hr to detect the VANGL2 signal.

For visualizing PDGFA localization and distribution in the lung, lungs from *Pdgfa^ex4COIN/+^; Sox2^Cre/+^* mice at P2 were collected and fixed in 4% PFA on ice for 1 hr. Lungs were washed in 0.02% NP40 in PBS for 2 hr, and placed in X-gal staining solution (5 mM K_3_Fe(CN)_6_, 5 mM K_4_Fe(CN)_6_, 2 mM MgCl_2_, 0.01% sodium deoxycholate, 0.02% NP-40, 1 mg/ml X-gal) for 16 hr at 37°C. LacZ-stained lungs were paraffin embedded and sectioned. Immunohistochemistry was subsequently performed on LacZ-stained sections using antibodies against HOPX (AT1 cell marker) and SPC (AT2 cell marker).

Images for H&E staining, elastin staining and LacZ staining were taken using a SPOT 2.3 CCD camera connected to a Nikon Eclipse E1000 microscope. Confocal images were captured on a Leica SPE laser-scanning confocal microscope. Adjustment of red/green/blue/grey histograms and channel merges were performed using LAS AF Lite (Leica Microsystems).

### Transmission electron microscopy (TEM)

Lungs from *Vangl2^f/f^; Sox9^Cre/+^* mice and their littermate controls were infused with EM fixative (2% glutaraldehyde and 4% paraformaldehyde in PBS) through the trachea. Dissected lungs were immersed in ice-cold EM fixative for at least 2 hr. The samples were trimmed to 1 mm cubes and sectioned at 0.1 μm. The ultrathin sections were placed on copper grids and post-stained. Grids were viewed and photographed with a JEOL JEM-1400 Transmission Electron Microscope at the Cell Sciences Imaging Facility at Stanford University.

### Measurement of the mean linear intercept (MLI)

The mean linear intercept (MLI) (Lm) was measured according to the previously reported methods (Nagendran et al., 2018) with minor modification. In brief, for each animal 15 fields of hematoxylin and eosin-stained lung sections from three different slides were imaged at 20x magnification using a Nikon Eclipse E1000 Microscope. Fields that contained visible blood vessels or airways were excluded. A grid with 10 horizontal and 10 vertical lines was superimposed on the images using ImageJ. The number of times that the alveolar walls (the primary and secondary septa) intercepted the grid lines was counted. Lm was calculated using the following equation: Lm = (Lh + Lv)/m, where Lh is the total length of horizontal line, Lv is the total length of vertical line, and m is the total number of intercepts.

### Cell proliferation assays

The rate of cell proliferation was determined through a short pulse of EdU labeling (Lin et al., 2017). Briefly, mouse pups at postnatal day 0, 2, 5 and 7 were intraperitoneally injected with 0.25 mg of EdU (50 μl of EdU/PBS solution at 5 mg/ml) and lungs were collected 1 hr following EdU injection. Assessment of EdU incorporation was performed on paraffin-embedded lung sections using the Click-iT EdU Alexa Fluor 488 Imaging Kit (Life Technologies). These lung sections were co-stained with antibodies against NKX2.1 or GFP.

For quantifying proliferation of alveolar fibroblasts and myofibroblasts, proliferating cells were labeled by EdU while fibroblasts/myofibroblasts were distinguished by H2BGFP expressed from the *Pdgfra* locus (the *Pdgfra^H2BGFP^* allele). The proliferation rate of alveolar fibroblasts/myofibroblasts was calculated as the ratio of (EdU^+^H2BGFP^+^ cells)/(H2BGFP^+^ cells).

Alternatively, alveolar fibroblasts and myofibroblasts were identified by anti-PDGFRA antibodies. In this case, the proliferation rate of alveolar fibroblasts/myofibroblasts was calculated as the ratio of (EdU^+^PDGFRA^+^ cells)/(PDGFRA^+^ cells).

### Proximity Ligation In Situ Hybridization (PLISH)

In situ hybridization was performed following the procedure of Proximity Ligation In Situ Hybridization (PLISH) (Nagendran et al., 2018) with minor modifications. Lungs from *Pdgfra^H2BGFP/+^* mice were dissected and fixed in 4% paraformaldehyde overnight and embedded in OCT for frozen sections at 16 μm. Lung sections were incubated with H probe pairs, which were targeted to the adjacent positions in a tiled manner along the mRNA of interest. Nine H probe pairs for mouse *Wnt5a* and ten H probe pairs for mouse *Ror2* were used (see Supplementary file 2). In the circularization reaction, the barcode sequences in the paired probes hybridized to the 'bridge' (phosphorylated VB02) and 'circle' (phosphorylated CCC2.1) oligonucleotides to form a closed circle, which underwent rolling circle replication to generate a long single-stranded amplicon of tandem repeats. Fluorescently-labeled oligonucleotides were hybridized to the complementary tandem repeats and generated bright puncta for visualization.

For combined in situ hybridization/immunohistochemistry, samples were washed 3 times in PBST (0.05% Tween-20 and 5 mM EDTA in PBS) after PLISH and incubated in blocking buffer (3% BSA, 0.1% Triton X-100 and 5 mM EDTA in PBS) for 30 min. The sections were then incubated with primary antibodies (goat anti-CC10 or chicken anti-GFP in blocking buffer) for 2 hr at room temperature, washed in PBST and then incubated with secondary antibodies (donkey anti-goat Alexa Fluor 488 or donkey anti-chicken Alexa Fluor 488) for 1 hr at room temperature.

### Lentivirus production and transduction

cDNAs encoding PDGFA-3xFLAG and PDGFA-eGFP fusions were cloned into the modified pSECC lentiviral vector. The mouse PDGFA (NM_008808.4) coding sequence was amplified from mouse P3 lung cDNA library.

Lentiviruses were produced in HEK293T cells grown in DMEM containing 10% FBS, 1x penicillin/streptomycin and 1x L-glutamine. One day before transfection, HEK293T cells were seeded at 50–60% confluence in a 6 cm dish; cells reached 80–90% confluence for transfection the following day. For HEK293T cells on one 6 cm dish, 1 μg of pMD2.G, 1 μg of psPAX2 and 2 μg of the plasmid of interest (PDGFA-3xFLAG or PDGFA-eGFP) were mixed in 500 μl OPTI-MEM and 30 μl of polyethylenimine (PEI) (1 μg/μl, transfection reagent) was added. Media were replaced 24 hr post-transfection and viral supernatants were harvested 48 hr post-transfection. The viral supernatants were filtered through 0.45 μm PVDF filters and then added to control and *Vangl1^gt/gt^; Vangl2^–/–^* adherent mouse cells together with 8 μg/ml polybrene. 12 hr after transduction, the media were replaced with regular culture media (DMEM with 10% FBS and 1% penicillin/streptomycin).

### PDGFA secretion assay

Control and *Vangl1^gt/gt^; Vangl2^–/–^* cells were derived from mouse embryos at 13.5 and 18.5 *dpc*, authenticated by methods such as PCR and STR profiling, and tested negative for mycoplasma contamination. Loss of *Vangl1/2* ensures a complete removal of signaling through VANGL1/2, These cell lines were lentivirally transduced with constructs that expressed 3xFLAG-tagged PDGFA as described above. Cells were seeded onto 6 cm dishes. Once cells reached 100% confluence, the media were replaced with OPTI-MEM supplemented with insulin, transferrin and selenium (ITS) and cells were cultured for another 16 hr. The supernatants were then collected and filtered through 0.45 μm filters. Protein inhibitor cocktails were added to the filtrates, which were centrifuged at high speed (>12000 rpm) for 15 min at 4° to remove cell debris and protein aggregates. The filtrates were further concentrated in protein concentration columns (Millipore CENTRICON YM-10 Centrifugal Filter Unit 2 mL 10 kDa) through centrifugation at 2000 g for 1 hr at 4°C. In parallel, cells on the 6 cm dishes were scraped for lysis. Immunoprecipitation (IP) buffer (50 mM Tris pH 7.4, 2 mM EDTA, 150 mM NaCl, 0.5% Triton X-100, 1x protein inhibitor cocktail) was added to the concentrated filtrates or scraped cells in a total volume of 500 μl. Immunoprecipitation was performed using FLAG-M2 beads following standard procedures. SDS-PAGE sample buffer was added to the immunoprecipitates for western blot analysis. PDGFA secretion was calculated using the following equation: P_secretion_ = P_s_/(P_s_ + P_c_) where P_s_ = PDGFA in supernatant/medium, P_c_ = PDGFA in the cell lysate.

### Alveolosphere assays

Alveolosphere assays were performed as previously described (Barkauskas et al., 2013) with minor modifications. Briefly, *Sftpc^CreER/+^; ROSA26^mTmG/+^* and *Vangl1^gt/gt^; Vangl2^f/f^; Sftpc^CreER/+^; ROSA26^mTmG/+^* mice were injected with tamoxifen to label alveolar type II (AT2) cells with eGFP. One month post-tamoxifen injection, lungs were dissected and digested in dispase (1.2 U/ml), collagenase B (0.5 mg/ml) together with 50 U/ml DNase at 37°C. After incubation for 1 hr, an equal volume of sorting media (phenol red free DMEM with 2% FBS and 2% penicillin/streptomycin) was added to the dissociated cells. The mixture was then passed through 70 μm cell strainers and centrifuged at 600 g for 10 min. Cell pellets were resuspended in 3 ml RBC lysis buffer (Invitrogen) for 2 min on ice and 5 ml sorting medium was added. The mixture was passed through the 40 μm cell strainer. After centrifugation at 600 g for 10 min, cell pellets were resuspended in 1 ml sorting buffer for fluorescence-activated cell sorting (FACS). eGFP-labeled AT2 cells were isolated using a BD FACSAria III sorter. 5 × 10^3^ sorted AT2 cells and 5 × 10^4^ Mlg 2908 cells (ATCC CCL-206, a lung fibroblast cell line) were added to 100 μl culture medium (DMEM/F12 supplemented with Glutamax, 10% FBS, 1x insulin/transferrin/selenium, 2x penicillin/streptomycin, 0.25 μg/ml Amphotericin B, 0.04 μg/ml EGF, 0.05 μg/ml bFGF, 0.02 μg/ml KGF, and 0.02 μg/ml HGF), mixed with an equal volume of Matrigel (BD Biosciences), and then seeded into a 0.4 μm Transwell insert (Corning) in a 24-well plate. The medium was replaced every two days. After three weeks of culture, the Transwell inserts were fixed in 4% paraformaldehyde at 4°C overnight and embedded in OCT. The organoid/Matrigel-containing inserts were sectioned at 50 μm for immunohistochemistry.

### RNA-Seq analysis

RNA-Seq was performed as previously described (Lin et al., 2017). In brief, the left lung lobe from control, *Vangl2^f/f^; Sox9^Cre/+^* and *Ror2^f/f;^ Sox9^Cre/+^* mice were homogenized in 1 ml TRIzol (Life Technologies) and 200 μl chloroform was then added. After centrifugation, the upper aqueous layer was removed and mixed with an equal volume of 70% ethanol. RNA was extracted with the RNeasy Mini Kit (Qiagen) following the manufacturer’s instructions. RNA quality was evaluated using the Agilent 2100 Bioanalyzer. Samples were sequenced on an Illumina HiSeq 2000 or HiSeq4000. Functional enrichment analysis was performed using Ingenuity Pathway Analysis software (version 7.1). Differential gene expression and gene ontology (GO) enrichment analyses were performed with RStudio. The barplot of gene ontology enrichment was created in order to visualize differentially expressed genes that are associated with a certain biological process (BP). Datasets have been deposited in NCBI’s Gene Expression Omnibus database and are accessible through GEO Series accession number GSE140779.

### qPCR analysis

RNAs extracted from lung tissues were reverse-transcribed with the Maxima First Strand cDNA Synthesis Kit (Thermo Scientific). Quantitative PCR (qPCR) was carried out on the ABI Prism 7900HT Sequence Detection System. Primers for qPCR are listed in the Sequence-based reagent table.

### Cell migration (wound recovery or healing) assay

The migratory capacity of lung myofibroblasts was determined using the Culture-Insert 2 Well system (ibidi). Briefly, lungs from control and *Vangl2^f/f^; Pdgfra^Cre/+^* mice at P3 were dissected and digested in dispase (1.2 U/ml) and collagenase B (0.5 mg/ml) at 37°C to release single cells. After incubation for 1 hr, an equal volume of culture medium (DMEM with 10% FBS and 2% penicillin/streptomycin) was added to dissociated cells. The mixture was then passed through a 40 μm cell strainer and centrifuged at 600 g for 10 min. Cell pellets were resuspended in 200 μl culture medium and seeded into wells (100 μl per well). Myofibroblasts were allowed to attach to fibronectin–coated plates for 2–3 hr. Non-adherent cells were washed out and myofibroblasts were cultured to reach 100% confluence in ~2–3 days. Confluent myofibroblasts were switched to starvation medium (DMEM with 0.2% FBS and 1% penicillin/streptomycin) for 16 hr prior to removal of the insert. Migration of myofibroblasts into the wounded area (insert) was allowed to continue for another 36–48 hr.

### Human lung tissues

Lung samples were obtained at the time of lung transplantation performed for severe emphysema (Global Initiative for Chronic Obstructive Lung Disease Criteria, stages III or IV) during the study period 2015‐2019. Control lung tissues were obtained from donor lungs not utilized for lung transplantation. Our studies indicate that these lungs are physiologically and pathologically normal (Ware et al., 2002). Written informed consent was obtained from all subjects and the study was approved by the University of California, San Francisco Institutional Review Board (IRB approval # 13–10738).

### Statistical analysis

Both technical replicates and biological replicates (using different cell lines and mice) were performed. All results were presented as mean ± SEM. Two-tailed Student’s *t*-tests were used to calculate the P values. Statistical significance was considered only when the P value was less than 0.05.

## Data Availability

RNA-Seq data have been deposited in GEO (Series accession number GSE140779). The following dataset was generated: ZhangKYaoEChuangPT2020A mammalian Wnt5a-Ror2-Vangl2 axis controls the cytoskeleton and confers cellular properties required for alveologenesisNCBI Gene Expression OmnibusGSE14077910.7554/eLife.53688PMC721770232394892 The following previously published dataset was used: GuoMDuYGokeyJJRayS2019Single cell RNA analysis identifies cellular heterogeneity and adaptive responses of the lung at birthNCBI Gene Expression OmnibusGSE12233210.1038/s41467-018-07770-1PMC631831130604742

## Additional files

Supplementary file 1. The Key Resources table lists antibodies, chemicals, peptides, recombinant proteins, commercial assays, cell lines, mouse strains, biological samples, recombinant DNA reagents, and software and algorithms used in this study.

Supplementary file 2. The Sequence-based reagent table lists oligonucleotides used in this study.

Transparent reporting form
