# Supplementary material for: A mammalian Wnt5a–Ror2–Vangl2 axis controls the cytoskeleton and confers cellular properties required for alveologenesis
Source: eLife. 2020 May 12;9:e53688. doi: 10.7554/eLife.53688 (PMC7217702; doi:10.7554/eLife.53688)
Supplement: Supplementary file 1. [file elife-53688-supp1.docx]

| **Key Resources Table** | | | | |
| --- | --- | --- | --- | --- |
| **Reagent type (species) or resource** | **Designation** | **Source or reference** | **Identifiers** | **Additional information** |
| antibody | Mouse anti-ACTA2 | Thermo Scientific Lab Vision | Cat# MS-113-P0; RRID:AB_64001 | IF (1:200) |
| antibody | Rabbit anti-Phospho-AKT (Ser473) (D9E) | Cell Signaling Technology | Cat# 4060; RRID:AB_2315049 | IF (1:150) |
| antibody | Goat anti-CC10 | Santa Cruz Biotechnology | Cat# sc-9773; RRID:AB_2183391 | IF (1:200) |
| antibody | Rabbit anti-Cofilin (D3F9) | Cell Signaling Technology | Cat# 5175; RRID:AB_10622000 | IF (1:150) |
| antibody | Rabbit anti-Phospho-Cofilin (Ser3) (77G2) | Cell Signaling Technology | Cat# 3313; RRID:AB_2080597 | IF (1:100) |
| antibody | Rat anti-E-cadherin | Invitrogen | Cat# 13–1900; RRID:AB_2533005 | IF (1:200) |
| antibody | Chick anti-GFP | Abcam | Cat# ab13970; RRID:AB_300798 | IF (1:200) |
| antibody | Mouse anti-HOPX | Santa Cruz Biotechnology | Cat# sc-398703; RRID:AB_2687966 | IF (1:100) |
| antibody | Rabbit anti-Phospho-LIMK1 (Thr508)/LIMK2 (Thr505) | Cell Signaling Technology | Cat# 384; RRID:AB_2136943 | IF (1:100) |
| antibody | Rabbit anti-NKX2.1 | Epitomics | Cat#2044–1; RRID:AB_1267367 | IF (1:100) |
| antibody | Rabbit anti-PDGF receptor alpha | Cell Signaling Technology | Cat# 3164; RRID:AB_2162351 | IF (1:150) |
| antibody | Rabbit anti-Phospho-PDGF Receptor α (Tyr754) (23B2) | Cell Signaling Technology | Cat# 2992; RRID:AB_390728 | IF (1:100) |
| antibody | Rat anti-PECAM-1 (MEC 13.3) | Santa Cruz Biotechnology | Cat# sc-18916; RRID:AB_627028 | IF (1:150) |
| antibody | Rabbit anti-prosurfactant protein C (proSP-C) | MilliporeSigma | Cat# AB3786; RRID:AB_91588 | IF (1:200) |
| antibody | Hamster anti-T1α | Developmental Studies Hybridoma Bank | Cat# 8.1.1;RRID:AB_531893 | IF (1:200) |
| antibody | Rat anti-VANGL2 clone 2G4 | MilliporeSigma | Cat# MABN750; RRID:AB_2721170 | IF (1:150) |
| chemical compound, drug | Amphotericin B | Sigma-Aldrich | Cat#A2942 |  |
| other | ANTI-FLAG® M2 Affinity Gel | Sigma-Aldrich | Cat#A2220 |  |
| peptide, recombinant protein | Basic Fibroblast Growth Factor (bFGF) | Gibco | Cat#13256-029 |  |
| other | Biotin-XX Phalloidin | Molecular Probes | Cat#B7474 |  |
| other | DMEM/F12 plus Glutamax | Mediatech | Cat#10-092-CV |  |
| other | Dynabeads | Invitrogen | Cat#11205D |  |
| peptide, recombinant protein | Epidermal Growth Factors (EGF) | Corning | Cat#354001 |  |
| other | Fetal Bovine Serum (FBS) | Gibco | Cat#10437-028 |  |
| peptide, recombinant protein | Fibronectin | Corning | Cat#354008 |  |
| other | Glutaraldehyde, 8% aqueous solution, EM grade | Electron Microscopy Sciences | Cat#16000 |  |
| peptide, recombinant protein | Hepatocyte Growth Factor (HGF) | Gibco | Cat#PHG0254 |  |
| other | Insulin, Transferrin and Selenium (ITS) | Gibco | Cat#51300-044 |  |
| peptide, recombinant protein | Fibroblast Growth Factor 7 (KGF/FGF-7) | R&D Systems | Cat#5028-KG-025 |  |
| chemical compound, drug | LGK974 | Toronto Research Chemicals | Cat# L397640 |  |
| other | Matrigel, Growth Factor Reduced (GFR) | Corning | Cat#356230 |  |
| other | Paraformaldehyde, 16% solution, EM grade | Electron Microscopy Sciences | Cat#15700 |  |
| other | Paraformaldehyde | Sigma-Aldrich | Cat# P6148 |  |
| other | Polyethylenimine (PEI) | Polysciences, Inc. | Cat# 23966-2 |  |
| other | Penicillin/Streptomycin | Gibco | Cat#15070-063 |  |
| other | Protease Inhibitor Cocktail | Biotool | Cat#B14001 |  |
| other | Rhodamine-conjugated phalloidin | Molecular Probes | Cat#R415 |  |
| other | TRIzol® Reagent | Ambion | Cat#15596018 |  |
| chemical compound, drug | Wnt-C59 | Toronto Research Chemicals | Cat# W499300 |  |
| commercial assay, kit | Click-iT™ EdUAlexa Fluor™ 488 Imaging Kit | Thermo Fisher | Cat#C10337 |  |
| commercial assay, kit | CentriprepUltracelYM-10 Centrifugal Filter Devices | MilliporeSigma | Cat#4305 |  |
| commercial assay, kit | RNeasy Mini Kit | QIAGEN | Cat#74104 |  |
| commercial assay, kit | TSA Plus Cyanine 3 (Cy3) Fluorescein detection kit | Perkin-Elmer | Cat#NEL753001KT |  |
| commercial assay, kit | Two-well culture insert | Ibidi | Cat#80209 |  |
| cell line | Mouse: *Vangl1 Vangl2* double knockout MEF cells | This paper | N/A | Refer to “Lentivirus production and transduction” section in Materials and methods. |
| genetic reagent (*Mus musculus*) | *Aqp5CreER* | This paper | N/A | Refer to “Generation of *Aqp5CreER* mice” section in Materials and methods. |
| genetic reagent (*Mus musculus*) | *Dermo1Cre* [*Twist2tm1.1(cre)Dor*/J] | David Ornitz,  (Yu et al.,2003) | N/A |  |
| genetic reagent (*Mus musculus*) | *FLPe* [Tg(ACTFLPe)9205Dym] | Susan Dymecki (Rodriguez et al., 2000) | RRID:IMSR_EM:02520 |  |
| genetic reagent (*Mus musculus*) | *PDGFαex4COIN* [Pdgfaex4COIN] | ChristerBetsholtz  (Johanna et al.,2014) | N/A |  |
| genetic reagent (*Mus musculus*) | *PDGFRαCre* [C57BL/6-Tg(Pdgfra-cre)1Clc/J] | The Jackson Laboratory | Stock# 013148, RRID:IMSR_JAX:013148 |  |
| genetic reagent (*Mus musculus*) | *PDGFRαCreER* [B6N.Cg-Tg(Pdgfra-cre/ERT)467Dbe/J] | The Jackson Laboratory | Stock# 018280, RRID:IMSR_JAX:018280 |  |
| genetic reagent (*Mus musculus*) | *PDGFRαH2BGFP* [B6.129S4-*Pdgfratm11(EGFP)Sor*/J] | The Jackson Laboratory | Stock# 007669, RRID:IMSR_JAX:007669 |  |
| genetic reagent (*Mus musculus*) | *Porcnf* [129S-*Porcntm1.1Vdv*/J] | The Jackson Laboratory | Stock# 020994, RRID:IMSR_JAX:020994 |  |
| genetic reagent (*Mus musculus*) | *Ror2f* [B6;129S4-*Ror2tm1.1Meg*/J] | The Jackson Laboratory | Stock#018354, RRID:IMSR_JAX:018354 |  |
| genetic reagent (*Mus musculus*) | *ROSA26mTmG* [*Gt(ROSA)26Sortm4(ACTB-tdTomato,-EGFP)Luo*/J] | The Jackson Laboratory | Stock# 007576, RRID:IMSR_JAX:007576 |  |
| genetic reagent (*Mus musculus*) | *ROSA26tdTomato* [B6;129S6-*Gt(ROSA)26Sortm14(CAG-tdTomato)Hze*/J] | The Jackson Laboratory | Stock# 007908, RRID:IMSR_JAX:007908 |  |
| genetic reagent (*Mus musculus*) | *SftpcCreER* [*Sftpctm1.1(cre/ERT2)Ptch*] | Pao-Tien Chuang  (Lin et al., 2012) | N/A |  |
| genetic reagent (*Mus musculus*) | *ShhCre* [B6.Cg-*Shhtm1(EGFP/cre)Cjt*/J] | The Jackson Laboratory | Stock#005622, RRID:IMSR_JAX:005622 |  |
| genetic reagent (*Mus musculus*) | *Sox2Cre* [B6.Cg-*Edil3Tg(Sox2-cre)1Amc*/J] | The Jackson Laboratory | Stock#008454, RRID:IMSR_JAX:008454 |  |
| genetic reagent (*Mus musculus*) | *Sox9Cre* [Sox9*tm3(Cre)Crm*] | Benoit de Crombrugghe (Haruhiko et al.,2005) | N/A |  |
| genetic reagent (*Mus musculus*) | *Tbx4Cre* [Tg*(Tbx4-cre)6Kras*] | Mark Krasnow  (Kumar et al.,2014) | RRID:MGI:5635890 |  |
| genetic reagent (*Mus musculus*) | *Vangl1gt* [Vangl1*GT(XL802)Byg*] | Yingzi Yang  (Song et al.,2010) | N/A |  |
| genetic reagent (*Mus musculus*) | *Vangl2f* [Vangl2*tm1.1Yy*] | Yingzi Yang  (Song et al.,2010) | N/A |  |
| genetic reagent (*Mus musculus*) | *Wnt5af* [B6;129S-*Wnt5atm1.1Krvl*/J] | The Jackson Laboratory | Stock# 026626, RRID:IMSR_JAX:026626 |  |
| biological sample (*Homo sapiens*) | Human Emphysema/COPD patient tissue (deidentified) | Paul Wolters | N/A |  |
| recombinant DNA reagent | Lentiviral vector backbone for *Pdgfa* cloning, modified from pSECC | This paper | N/A | Refer to the “Lentivirus production and transduction” section of Materials and methods. |
| recombinant DNA reagent | pSECC | Sanchez-Rivera et al., 2014 | Addgene Plasmid #60820 |  |
| recombinant DNA reagent | pMD2.G | A gift from Didier Trono | Addgene Plasmid #12259 |  |
| recombinant DNA reagent | psPAX2 | A gift from Didier Trono | Addgene Plasmid #12260 |  |
| recombinant DNA reagent | mEmerald-ER-5 | A gift from Michael Davidson | Addgene Plasmid #54083 |  |
| recombinant DNA reagent | mEmerald-Golgi-7 | A gift from Michael Davidson | Addgene Plasmid #54108 |  |
| software, algorithm | Heatmapper |  | RRID:SCR_016974 | http://www.heatmapper.ca/ |
| software, algorithm | ImageJ |  | RRID:SCR_003070 | https://imagej.nih.gov/ij/ |
| software, algorithm | Prism 5.0 | GraphPad | RRID:SCR_002798 | https://www.graphpad.com/ |
| software, algorithm | RStudio | RStudio® | RRID:SCR_000432 | https://www.rstudio.com/ |
