# Supplementary material for: A mammalian Wnt5a–Ror2–Vangl2 axis controls the cytoskeleton and confers cellular properties required for alveologenesis
Source: eLife. 2020 May 12;9:e53688. doi: 10.7554/eLife.53688 (PMC7217702; doi:10.7554/eLife.53688)
Supplement: Supplementary file 2. [file elife-53688-supp2.docx]

**Supplementary File**

| Sequence-based reagent |
| --- |
| PLISH primer: *Mus musculus Ror2* (fwd):  5’-AGGTCAGGAATACTTAGCTATTGATGGTGGCTCCAGAAAATTCAGAA-3’ |
| PLISH primer: *Mus musculus Ror2* (rev):  5’-ACAATGGTGATATTGTTGACTTCTGTGTAGACGACTATAGCCAGGTT-3’ |
| PLISH primer: *Mus musculus Ror2* (fwd):  5’-AGGTCAGGAATACTTAGCTATTGATGGATAGAACCCCAGTGGCAGTG-3’ |
| PLISH primer: *Mus musculus Ror2* (rev):  5’-CGTCGGACCGAGCCGCACATTTCTGTGTAGACGACTATAGCCAGGTT-3’ |
| PLISH primer: *Mus musculus Ror2* (fwd):  5’-AGGTCAGGAATACTTAGCTATTGATGGACAGAATCCCCATCTTGCTG-3’ |
| PLISH primer: *Mus musculus Ror2* (rev):  5’-AATGCTGGGGACCAGGATGTTTCTGTGTAGACGACTATAGCCAGGTT-3’ |
| PLISH primer: *Mus musculus Ror2* (fwd):  5’-AGGTCAGGAATACTTAGCTATTGATGGTTTGAGTTTGGCCTGTTTGT-3’ |
| PLISH primer: *Mus musculus Ror2* (rev):  5’-ACTGTGGACAAGCTGATCTCTTCTGTGTAGACGACTATAGCCAGGTT-3’ |
| PLISH primer: *Mus musculus Ror2* (fwd):  5’-AGGTCAGGAATACTTAGCTATTGATGGTCACATTGCTTACAGGGCTG-3’ |
| PLISH primer: *Mus musculus Ror2* (rev):  5’-GGCCATATAGCGGGCATTGCTTCTGTGTAGACGACTATAGCCAGGTT-3’ |
| PLISH primer: *Mus musculus Ror2* (fwd):  5’-AGGTCAGGAATACTTAGCTATTGATGGTCTCACAGTCTCAGGTGGGT-3’ |
| PLISH primer: *Mus musculus Ror2* (rev):  5’-GCAGGAGATAGTAAAAAATCTTCTGTGTAGACGACTATAGCCAGGTT-3’ |
| PLISH primer: *Mus musculus Ror2* (fwd):  5’-AGGTCAGGAATACTTAGCTATTGATGGTGTCCTGTTTCTGAGGTGGT-3’ |
| PLISH primer: *Mus musculus Ror2* (rev):  5’-AGACAGAGGCGGGGTCTCCCTTCTGTGTAGACGACTATAGCCAGGTT-3’ |
| PLISH primer: *Mus musculus Ror2* (fwd):  5’-AGGTCAGGAATACTTAGCTATTGATGGTGTTTCTGTGCCCAATATCC-3’ |
| PLISH primer: *Mus musculus Ror2* (rev):  5’-AGGGCGGTGAAGATGTAGCCTTCTGTGTAGACGACTATAGCCAGGTT-3’ |
| PLISH primer: *Mus musculus Ror2* (fwd):  5’-AGGTCAGGAATACTTAGCTATTGATGGTAAGCTCCAAGGTGCCTCTC-3’ |
| PLISH primer: *Mus musculus Ror2* (rev):  5’-CAGGGGGCTCACGGAGCATCTTCTGTGTAGACGACTATAGCCAGGTT-3’ |
| PLISH primer: *Mus musculus Ror2* (fwd):  5’-AGGTCAGGAATACTTAGCTATTGATGGTCCCAAGTGTTCAGAAGCAA-3’ |
| PLISH primer: *Mus musculus Ror2* (rev):  5’-CTCTCTTTAATACAAGGCACTTCTGTGTAGACGACTATAGCCAGGTT-3’ |
| PLISH primer: *Mus musculus Wnt5a* (fwd):  5'-AGGTCAGGAATACTTAGCTATTGATGGAAGCCTTGGGGGACAATCCA-3' |
| PLISH primer: *Mus musculus Wnt5a* (rev):  5'-TCAAGCGAAGCGTCGGGGTTTTCTGTGTAGACGACTATAGCCAGGTT-3' |
| PLISH primer: *Mus musculus Wnt5a* (fwd):  5'-AGGTCAGGAATACTTAGCTATTGATGGTCCCGGGCTTAATATTCCAA-3' |
| PLISH primer: *Mus musculus Wnt5a* (rev):  5'-CCAGCGGTCCCCAAAGCCACTTCTGTGTAGACGACTATAGCCAGGTT-3' |
| PLISH primer: *Mus musculus Wnt5a* (fwd):  5'-AGGTCAGGAATACTTAGCTATTGATGGAGGAAGAACTTGGAAGACAT-3' |
| PLISH primer: *Mus musculus Wnt5a* (rev):  5'-AAAACGTGGCCAAAGCCATTTTCTGTGTAGACGACTATAGCCAGGTT-3' |
| PLISH primer: *Mus musculus Wnt5a* (fwd):  5'-AGGTCAGGAATACTTAGCTATTGATGGATAACAACCTGGGCGAAGGA-3' |
| PLISH primer: *Mus musculus Wnt5a* (rev):  5'-ACCACCAAGAATTAGCTTCTTTCTGTGTAGACGACTATAGCCAGGTT-3' |
| PLISH primer: *Mus musculus Wnt5a* (fwd):  5'-AGGTCAGGAATACTTAGCTATTGATGGTGCCTATTTGCATCACCCTG-3' |
| PLISH primer: *Mus musculus Wnt5a* (rev):  5'-CGTGAAGGCCGTCTCTCGGCTTCTGTGTAGACGACTATAGCCAGGTT-3' |
| PLISH primer: *Mus musculus Wnt5a* (fwd):  5'-AGGTCAGGAATACTTAGCTATTGATGGTCTAGCGTCCACGAACTCCT-3' |
| PLISH primer: *Mus musculus Wnt5a* (rev):  5'-GCGTGGATTCGTTCCCTTTCTTCTGTGTAGACGACTATAGCCAGGTT-3' |
| PLISH primer: *Mus musculus Wnt5a* (fwd):  5'-AGGTCAGGAATACTTAGCTATTGATGGACTGTCCTACGGCCTGCTTC-3' |
| PLISH primer: *Mus musculus Wnt5a* (rev):  5'-CTACATCTGCCAGGTTGTATTTCTGTGTAGACGACTATAGCCAGGTT-3' |
| PLISH primer: *Mus musculus Wnt5a* (fwd):  5'-AGGTCAGGAATACTTAGCTATTGATGGTAATTTCTGGGGACCTCCCT-3' |
| PLISH primer: *Mus musculus Wnt5a* (rev):  5'-GTAGGAAACTTCAAGTTTTCTTCTGTGTAGACGACTATAGCCAGGTT-3' |
| PLISH primer: *Mus musculus Wnt5a* (fwd):  5'-AGGTCAGGAATACTTAGCTATTGATGGATGTGGTGAGCTGGTTTGCT-3' |
| PLISH primer: *Mus musculus Wnt5a* (rev):  5'-ATTGTTTAAACTAGCTATCTTTCTGTGTAGACGACTATAGCCAGGTT-3' |
| qPCR primer: *Mus musculus Acta2* (fwd):  5’-ATGCAGAAGGAGATCACAGC-3’ |
| qPCR primer: *Mus musculus Acta2* (rev):  5’-GAAGGTAGACAGCGAAGCC-3’ |
| qPCR primer: *Mus musculus Eln*(fwd):  5’-GCCAAAGCTGCCAAATACG-3’ |
| qPCR primer: *Mus musculus Eln*(rev):  5’-CTCCAGCTCCAACACCATAG-3’ |
| qPCR primer: *Mus musculus Pdgfrα* (fwd):  5’-CTGGCTCGAAGTCAGATCCACA-3’ |
| qPCR primer: *Mus musculus Pdgfrα* (rev):  5’-GACTTGTCTCCAAGGCATCCTC-3’ |
| qPCR primer: *Mus musculus Pdgfrα* (fwd):  5’-TGCAGTTGCCTTACGACTCCAGAT-3’ |
| qPCR primer: *Mus musculus Pdgfrα* (rev):  5’-AGCCACCTTCATTACAGGTTGGGA-3’ |
| qPCR primer: *Mus musculus Gapdh* (fwd):  5’-AGGTTGTCTCCTGCGACTTCA-3’ |
| qPCR primer: *Mus musculus Gapdh* (rev):  5’-CCAGGAAATGAGCTTGACAAAGTT-3’ |
| qPCR primer: *Homosapiens VANGL1* (fwd):  5’-TGAAGAGGCCGAACATGAAC-3’ |
| qPCR primer: *Homo sapiens VANGL1* (rev):  5’-AGACGCTGAATGTGGATGAAG-3’ |
| qPCR primer: *Homo sapiens VANGL2* (fwd):  5’-CTCAGTTCACGCTCAAGGTC-3’ |
| qPCR primer: *Homo sapiens VANGL2* (rev):  5’-GTAATACTTCTCCAGGATCCACAC-3’ |
| qPCR primer: *Homo sapiens WNT5A* (fwd):  5’-AGTTCTTCCTAGTGGCTTTGG-3’ |
| qPCR primer: *Homo sapiens WNT5A* (rev):  5’-TCTGACATCTGAACAGGGTTATTC-3’ |
| qPCR primer: *Homo sapiens GAPDH* (fwd):  5’-CTGACTTCAACAGCGACACC-3’ |
| qPCR primer: *Homo sapiens GAPDH* (rev):  5’-TAGCCAAATTCGTTGTCATACC-3’ |
| qPCR primer: *Homo sapiens ACTB* (fwd):  5’-GGATGCAGAAGGAGATCACTG-3’ |
| qPCR primer: *Homo sapiens ACTB* (rev):  5’-CGATCCACACGGAGTACTTG-3’ |
| qPCR primer: *Homo sapiens TUBA* (fwd):  5’-CGGAAGACAGAAGTACAGAGC-3’ |
| qPCR primer: *Homo sapiens TUBA* (rev):  5’-CAGCTCATTTGGGTTTCGTG-3’ |
