# Supplementary material for: A mammalian Wnt5a–Ror2–Vangl2 axis controls the cytoskeleton and confers cellular properties required for alveologenesis
Source: eLife. 2020 May 12;9:e53688. doi: 10.7554/eLife.53688 (PMC7217702; doi:10.7554/eLife.53688)
Supplement: Peer review history

# Decision letter

**DOI:** 10.7554/eLife.53688.sa1

**Affiliations:** University of PennsylvaniaUnited States

In the interests of transparency, eLife publishes the most substantive revision requests and the accompanying author responses.

Thank you for submitting your article "A *Wnt5a-Ror2-Vangl2* axis controls the cytoskeleton and confers cellular properties required for alveolar formation" for consideration by *eLife*. Your article has been reviewed by three peer reviewers, and the evaluation has been overseen by a Reviewing Editor and Edward Morrisey as the Senior Editor. The reviewers have opted to remain anonymous.

The reviewers have discussed the reviews with one another and the Reviewing Editor has drafted this decision to help you prepare a revised submission.

As you will see, while the reviewers acknowledged the merits in your study, there were many concerns related to both lack of data for the conclusions made and the confusing manner in which the manuscript is organized. All of the reviewers felt that the current manuscript should be pared down to focus on the points that can be substantiated with data. Please note in particular the concerns raised by reviewer #1 in relation to data needed to support the major conclusions of this manuscript.

Reviewer #1:

In Zhang et al., the authors investigate the role of *Wnt5a-Ror2-Vangl2* in alveologenesis and alveoli injury repair. They show extensive data from both epithelium and mesenchymal knockouts of a number of genes in this pathway, as well as Pdgfa mutants. Multiple mechanisms have been implicated, including affecting cell signaling, morphology, migration, cell-cell junction, ER and Golgi morphology. The findings do demonstrate a role for *Wnt5a-Ror2-Vangl2* in alveologenesis. However, many of the conclusions on mechanisms are not well supported. There is lack of logic in the organization of the results, which jump from epithelium to mesenchyme and back, from bleomycin in mice and then to COPD patient samples.

1) Throughout the paper, there is an overall paucity of quantification, of MLI, of protein expression level, etc.

2) Figure 1—figure supplement 1, *Sox2*-Cre;*Vangl2* mutant was not really described in the text. Where is the cre active, and how results from this mutant fit in with the rest of the data is unclear.

3) *Vangl1;2;Sox9^cre^* mutant, it will be informative to know lung phenotype, on whether the defects are in branching or sacculation.

4) Why *Shh^cre^*, a robust cre, is unable to recombine *Vangl2*-fl is not explained. Is there a change in RT-PCR? Why is *Sox9^cre^* able to recombine *Vangl2*-fl but not *Shh^cre^*?

5) Does inactivation of Porc in the epithelium lead to a defect?

6) Figure 3E*Pdgfa* expression in AT1 and AT2 cells, not convincing.

7) "….levels of PDGFRa were unaffected (Figure 3G)". However, by RNAseq, "*Pdgfra* expression was downregulated." Discrepancy?

8) The specificity of p-PDGFRa antibody needs to be demonstrated.

9) Decrease in AKT signaling needs to be shown.

10) The secretion assay, double null mutants were used. It is unclear what cells were isolated.

11) Given that ER and Golgi are affected, is protein processing/secretion a general defect, or is PDGFA signal specifically affected?

12) Figure 3J, 3L, need to show a field of cells before zooming in. Quantification is needed.

13) The finding that *Vangl;Spc-^cre^* mutants showed simplification phenotype suggests that Vangl is required in AT2 cells. It does support that the phenotype is due to secretion defect, as concluded in subsection “Alveolar epithelial cells lacking *VANGL2* fail to present the PDGF ligand to mesenchymal myofibroblasts”.

14) Figure 3K, only show histology, not "areas of defective alveolar development that was associated with loss of *Vangl1/2*". Antibody staining is needed to link mosaic inactivation to regional phenotype.

15) Subsection “Alveolar epithelial cells lacking *VANGL2* fail to present the PDGF ligand to mesenchymal myofibroblasts”, unclear how the defect in cell contact is needed for PDGF secreted signaling.

16) "Conversely, loss of PDGF ligand resulted in depletion of WNT5A-producing fibroblasts/myofibroblasts. These results support a positive feedback loop between WNT5A and PDGFA…" The staining shows in Pdgfa mutants the expression of PDGFRA expression. It does not show regulation of WNT5A. To demonstrate feedback, Wnt5a expression needs to be assayed in PDGF pathway mutants.

17) Aside from in vitro migration assay, there is no other support for migration defect of Vangl mutants. Myofibroblast migration defects shown in *Pdgfa* mutant was found during sacculation stages. Is there an in vivo defect during sacculation of altered distribution of myofibroblasts?

18) Figure 6A, it is unclear how AT1 that lack GFP, which presumably is not cre-recombined, show increase of p-Cofilin.

19) To really demonstrate AT1 wrapping of other cell types, thick section and 3D rendition is needed.

20) In the smaller spheres, are there fewer cells, or smaller AT1 cells?

21) For bleomycin experiment, het mutant control needs to be used. What is the phenotype on fibrosis?

22) It is not clear why fibrosis was studied in mouse, but COPD was studied in patients.

Reviewer #2:

In the manuscript, "A *Wnt5a-Ror2-Vangl2* axis controls the cytoskeleton and confers cellular properties required for alveolar formation" by Kuan Zhang and colleagues, the authors examine the role of the planar cell polarity (PCP) pathway in alveologenesis. The authors identify a *Wnt5a-Ror2-Vangl2* cascade that involves PDGF secretion from alveolar type I and type II cells, and results in cell shape changes of type I cells and migration of myofibroblasts due to changes in the cytoskeleton.

The studies presented in the manuscript are comprehensive, well-designed and presented, and provide novel insights into the role of the PCP pathway in alveologenesis. These findings are an important and useful addition to the field. However, there are several issues that could be addressed prior to publication.

1) It is interesting that the *Shh-Cre* did not result in deletion of *Vangl2*, particularly given that the authors appear to show excellent recombination of the *ROSA26-mTmG* allele, and that is Cre is known to be strongly expressed in the lung epithelium. Do the authors have any explanation why?

2) Although SOX9 is well known to be expressed in the distal lung epithelium during development, it is also expressed in the lung mesenchyme. Because the authors use the SOX9-Cre to characterize the effects of epithelial-specific loss of *Vangl2*, they should provide (ideally quantitative) data about the degree of recombination that the *Sox9-Cre* induces in the lung mesenchyme. One straight forward way to do this would be to add data using their *Sox9-Cre;ROSA26-mTmG* mice co-stained with a pan-epithelial marker such as *Cdh1* or *Nkx2-1*. Significant recombination in the mesenchyme could significantly impact the interpretation of their data.

3) Why did the authors choose to use the *Sox2*-*Cre* when they were primarily interested in the distal epithelium (Figure 3E) – why not just stick with the *Sox9-Cre* (especially since these data are shown in the next panel, Figure 3F)

4) I don't understand the rationale for using whole-lung as the samples for RNA-Seq instead of FACS-sorted cells, especially when the authors had *Sox9-Cre;ROSA26-mTmG;Vangl2* mice available, which would of easily permitted isolation of epithelial (and if desired, separate mesenchymal) cell population for RNA-Seq. The problem with a whole-lung bulk-seq analysis in this case, particularly when the authors have showed that epithelial loss of *Vangl2* results in decreased proliferation in the mesenchyme is that the differential expressed gene list will be dramatically skewed by the alterations in cell types. I don't think the authors necessarily need to re-do these experiments for publication, but they should significantly limit the conclusions that they draw from these data. FACS-sorted cell populations or scRNA-Seq would have been preferred.

5) In Figure 1—figure supplement 4, the alveoli appear somewhat compressed for P7-P30, particular P12-P30. Are these samples inflation-fixed? If not, this would be ideal, as this will impact the appearance of the alveolar structure, especially the relative thinness or thickness, as is trying to be illustrated. This may impact the authors’ conclusion about how the relative thinness/thickness changes throughout alveolar development.

6) The organoid data are somewhat difficult to interpret and doesn't seem to add much to the paper, but probably no harm in showing it either. While the organoids appear to be smaller, none of the organoid data provides any direct evidence why this would be. The fact that AT1 and AT proliferation appeared to be unaffected is probably the most useful observation. It may be worth just showing this directly in their knockout mouse model.

7) Although the authors showed that AT1 and AT2 cell proliferation seemed to be unaffected following loss of VANGL1/VANGL2, it is probably a bit of a stretch (without additional data) that the reason for the impaired lung regeneration following bleomycin injury with *Vangl1/Vangl2* deletion induced by *SPC-^Cre^-ERT2* is an AT1 mediated defect.

Reviewer #3:

This is a very timely and informative work. The authors have done a great and quite extensive analysis of the role of Wnt5a in the lung. The results will be of general interest and citable by other investigators in the field.

There are some problems:

1) The manuscript is unnecessarily extensive and diffused. The authors have piled up a vast amount of experimental designs some of which were just negative findings that lead them to the next set of studies, which turned out to be positive. The authors need to cull these extra data out of the manuscript and make it cogent as the alternative (the present form) reduces the impact of their findings.

2) The bulk of the genetic experiments used conventional knockout models. To be able to use the title that refers to alveologenesis, the authors had the option of using conditional (temporal) KO models, or they will have to show that the phenotypes they observe is not due to secondary causes of early development caused by the KO. This requires examining the KO mice in the embryonic stage.

Aletrnativel the authors may choose to change the title.

3) If no "No obvious difference in the gross morphology of the lungs was noticed between control and *Vangl2^f/f^;Sox9^Cre/+^*mice prior to postnatal day 3" what was the reason for death on PN2?

4) The authors claim that Vangl1 plays a minor role in

alveolar formation and loss of Vangl1 is compensated by *Vangl2* in alveologenesis". As these were conventional KO mice, how can the authors know that Vangl1 has a role in alveolar formation?

5) It is interesting, as the authors state, that a small number of *Vangl2^f/f^; Sox9^Cre/+^* mice survived beyond postnatal day 7." What was the histology of these lungs?

6) In *Vangl2^f/f^; Sox9^Cre/+^* embryos, is there a phenotype?

7) In the studies using cells from control and *Vangl1/2*-deficient cells, what type of cells were isolated and how?

8) Figure 3E is supposed to show the level of PDGFA (LacZ). But, B-gal staining is not quantifiable. Nor is there an apparent significant difference.

9) In the bleo model in which *Vangl1/2*-deficient AT1 cells were compromised in their ability to form new alveoli, the authors should use a quantifying method such as MLI in areas of destroyed alveoli.

10) The authors state: "We noticed that the primary septa in control lungs became visibly thinner during the first three days of postnatal life likely due to flattening of AT1 cells". Better and more convincing data would be required to include here.

[Editors' note: further revisions were suggested prior to acceptance, as described below.]

Thank you for resubmitting your work entitled "A mammalian *Wnt5a-Ror2-Vangl2* axis controls the cytoskeleton and confers cellular properties required for alveologenesis" for further consideration by *eLife*. Your revised article has been evaluated by Edward Morrisey (Senior Editor) and a Reviewing Editor.

The manuscript has been improved but there are some remaining issues that need to be addressed before acceptance, as outlined below:

We ask that you revise the text of your manuscript to more accurately describe your results. As reviewer 2 has noted, there are several unsubstantiated claims and unclear descriptions of some of your experiments. While we do not anticipate additional experiments are required to address these issues, please be advised that accuracy and rigor are important hallmarks of all *eLife* papers.

Reviewer #1:

The authors made a tremendous effort in revision. The study is much improved and is now suitable for publication.

Reviewer #2:

In their manuscript "A mammalian *Wnt5a-Ror2-Vangl2* axis controls the cytoskeleton and confers cellular properties required for alveologenesis", Dr. Zhang and colleagues present exciting and timely data from their studies of the role of PCP signaling during alveologenesis. In response to reviewers, they have made a number of significant improvements to the manuscript, including the additional of substantial new data. However, the manuscript itself continues to be limited by a number of conclusions that are either over-stated or not well supported by the data, and benefits from further revision.

1) Given the mortality timeline described in the manuscript, including some long-term survivors, in *Sox9 ^Cre^/wt; Vangl2 ^f/f^* mice, the addition of a Kaplan-Meier survival curve would be more robust than a text description of their observations.

2) The authors state that the "initial expansion of fibroblasts/myofibroblasts was unaffected (Figure 5A)" (in *Pdgfra ^Cre^/wt; Vangl2 ^f/f^* mice) and that "the primary role of mesenchymal PCP signaling is to control myofibroblast migration and function." In Figure 5A, while there appears to be no affect on proliferation of these cells at P3, there is a dramatic reduction in the number of EdU^+^ cells at P5.

3) The authors state that "abnormal myofibroblast development first appeared on day 2…" What exactly do they mean by this? If it’s decreased proliferation then this should be stated. If they mean something else, then the data to support it should be directly referenced.

4) The authors state (in regards to the mesenchymal conditional knock-out of Porcupine), that "these results support the notion that non-canonical Wnt signaling in the lung mesenchyme initiate PCP signaling for alveolar development." Although the paper as a whole presents a variety of data that supports this concept, the manuscript contains a number of statements like this over-interpreting individual experiments. As the authors themselves state, porcupine is required for the release of all Wnt ligands, so this particular experiment does not differentiate between the roles of canonical and non-canonical Wnt signaling in alveolar development.

5) Similarly, from the same set of experiments the authors conclude that there is "no effect on differentiation of AECs." The only data presented to support this statement are two images from T1a/SPC double-stains of wild-type versus conditional KO lungs. At most, from these data you could say that there was no obvious difference in the number of type 1 and type 2 AECs, but even that statement would require counting cell numbers across a number of animals and sections.

6) The inclusion of the SPC conditional knockout seems out of place. The rest of the manuscript focuses on type 1 AECs, so these data do not seem to add much to an already long manuscript.

7) In the earlier portion of the manuscript, the authors focus on conditional knockouts of *Vangl2* in various cellular compartments, but then for the intracellular trafficking studies, used cells derived from *Vangl1^gt/gt^; Vangl2^-/-^* embryos. Why was the double-knockout studied instead cells deficient in Vangl2 only? Did the *Vangl2* deficient cells not have the same phenotype?

8) The images shown do not really seem to support the conclusion that PGFA-GFP is trapped within the ER. In Figure 3J, the PDGFA expression seems to be in a fairly uniform peri-nuclear distribution (not in the same punctate loci as the ER stain), and in the images in Figure 3—figure supplement 4 the PDGFA seems to just be uniformly distributed through the cytoplasm rather than "trapped in the ER."

9) Although I don't think there is anything wrong with the RNA-Seq data presented, I think is value to the manuscript continues to be significantly limited by the fact that it is derived from whole lung tissue in a knockout where the authors have already shown has dramatic alterations in the ratios of certain cell populations (e.g. depletion of myofibroblasts). In this situation, the conclusions that can be drawn a gene ontology analysis of these data are very limited (the GO categories will largely represent biological functions of cell populations that are enriched or depleted in the cKO relative to control). It really adds little to the data already presented in the paper, and doesn't really address the question that set out to answer with the experiment (e.g. "…to reveal the pathways controlled by epithelial PCP signaling.").

10) The manuscript could benefit from more clarity about what the authors think is happening in their injury model. Looking at the two images shown in Figure 6—figure supplement 1, the airspaces appear to be larger. However, the measured MLI for the conditional knockout is smaller. Do the authors think the alveoli are smaller after injury? If so, why? Also, their conclusions that "AT1 cells were compromised in their ability to form new alveoli" is a dramatic over-interpretation of these data, particularly given that the *Sftpc-^CreER^* driver was used. While I agree there definitely appears to be an affect on injury repair, whether the defect is with type 1 or type 2 AECs or both, and what exactly the defect is remains entirely unclear.

11) Again, the organoid data are intriguing but I'm not sure how much it really adds to the story. I suppose it supports the general concept that *Vangl2* is required for general cellular organization, but again exactly what is happening is pretty difficult to really understand.

Reviewer #3:

This was a work that in my first review, I found to be very timely and significant. I was impressed by the number of approaches and the volume of the obtained data. I had concerns about the way the data were presented. The authors have rewritten the manuscript and it is now in much shape. I also had concern as to whether the authors were actually examining the process of alveologenesis or rather the events prior to it that ultimately lead to defects in this process.

The authors have responded to my concerns and I am satisfied with their response. They have additionally added data that strengthens their conclusions. I am still not absolutely convinced that their findings are directly relevant to postnatal alveologenesis, but I admit this is splitting hair. So, all in all, I think the manuscript represents high quality multidimensional data that make it meritorious for dissemination to the field. The work will definitely have an impact on our understanding of alveologenesis.

# Reply: Author response

**DOI:** 10.7554/eLife.53688.sa2

Reviewer #1:

1) Throughout the paper, there is an overall paucity of quantification, of MLI, of protein expression level, etc.

In response to the reviewer’s comment, we have added quantification of MLI (*e.g.*, in Figures 1, 2, 6, and in figure supplements), of protein expression levels (*e.g.*, in Figure 3), etc. We also would like to point out it is not practical to use Western blots to quantify protein expression for this purpose. For instance, PDGFRA is expressed in airway smooth muscle cells, vascular smooth muscle cells and alveolar myofibroblasts. The mutants we study in this manuscript selectively affect alveolar myofibroblasts. Thus, western blots of the whole lungs will not reveal changes in PDGFRA in these mutants. Also, there is no easy way to sort alveolar myofibroblasts away from airway smooth muscle cells and vascular smooth muscle cells for protein analysis. As a result, we have relied on quantification of immunoreactivity (*e.g.*, PDGFA, PDGFRA) in individual lung cells.

2) Figure 1—figure supplement 1, Sox2-cre;Vangl2 mutant was not really described in the text. Where is the cre active, and how results from this mutant fit in with the rest of the data is unclear.

*Sox2-Cre* is known to induce recombination in all epiblasts by embryonic (E) day 6.5 and has been used to generate the null mutant from a floxed allele. In this regard, *Vangl2^f/f^; Sox2^Cre/+^* is effectively *Vangl2^–/–^*. This provides an important tool to test the validity of VANGL2 antibodies. In fact, several commercially available VANGL2 antibodies we have tested yielded identical signals between control and *Vangl2^–/–^* lungs.

3) Vangl1;2;Sox9^cre^ mutant, it will be informative to know lung phenotype, on whether the defects are in branching or sacculation.

*Vangl1/2; Sox9-Cre* mutant lungs display no apparent defects in branching or sacculation. This information is now included in Figure 1—figure supplement 3.

4) Why Shh^cre^, a robust cre, is unable to recombine Vangl2-fl is not explained. Is there a change in RT-PCR? Why is Sox9^cre^ able to recombine Vangl2-fl but not Shh^cre^?

We were also surprised that *Shh-Cre*, which is broadly expressed and widely used, was unable to effectively remove VANGL2 in the lung. We observed the presence of *Vangl2^–^* (null; converted from *Vangl2^f^* by *Shh-Cre*) by PCR but only a few cells in the proximal airway lost VANGL2 (Figure 1—figure supplement 4) and thus no phenotypes were observed. We do not know why *Sox9-Cre* was more effective than *Shh-Cre* in converting *Vangl2^f^* to *Vangl2^–^*. Perhaps, this is related to the relatively large distance between the two loxP sites in *Vangl2^f^* and a higher level of Cre expression is required. We also won't be surprised if *Shh-Cre* is unable to effectively convert other floxed alleles.

5) Does inactivation of Porc in the epithelium lead to a defect?

We have utilized *Sox9-Cre, Shh-Cre* and *Nkx2.1-Cre* to inactivate *Porcn* and generated *Pornc^f/f^; Sox9^Cre/+^; Pornc^f/f^; Shh^Cre/+^* and *Pornc^f/f^; Nkx2.1^Cre/+^* mice. *Pornc^f/f^; Sox9^Cre/+^* mice died soon after birth due to craniofacial defects but lung branching appeared to be normal. *Pornc^f/f^; Shh^Cre/+^* and *Pornc^f/f^; Nkx2.1^Cre/+^* mice survived after birth without apparent branching or alveolar defects. These results suggest that inactivation of epithelial *Porcn* does not lead to a defect.

6) Figure 3E Pdgfa expression in AT1 and AT2 cells, not convincing.

We have quantified PDGFA expression in AT1 and AT2 cells (Figure 3E). More AT1 cells expressed a higher level of PDGFA than AT2 cells.

7) "…levels of PDGFRa were unaffected (Figure 3G)". However, by RNAseq, "Pdgfra expression was downregulated." Discrepancy?

Downregulation of *Pdgfra* expression by bulk RNA-Seq primarily reflects the reduced number of myofibroblasts in the mutant lungs (Figure 3C). However, the level of PDGFRA in individual myofibroblasts was unaffected (Figure 3G), implying that *Pdgfra* transcription at the single cell level was unaltered.

8) The specificity of p-PDGFRa antibody needs to be demonstrated.

We have added data (in Figure 3—figure supplement 2) to demonstrate the specificity of p-PDGFRA. We showed that p-PDGFRA displayed a similar expression pattern to that of PDGFRa since both co-localized with SMA in both airway smooth muscle cells and alveolar myofibroblasts. In *Pdgfa^–/–^* lungs, myofibroblasts were lost and p-PDGFRA immunoreactivity also disappeared. This confirmed the specificity of p-PDGFRA on lung tissues. Of note, p-PDGFRA antibodies only worked on frozen sections with TSA amplification.

9) Decrease in AKT signaling needs to be shown.

This piece of information has been added to Figure 3—figure supplement 3.

10) The secretion assay, double null mutants were used. It is unclear what cells were isolated.

We have employed two types of cells, one from E13.5 mouse embryonic fibroblasts and another from E18.5 lung fibroblasts.

11) Given that ER and Golgi are affected, is protein processing/secretion a general defect, or is PDGFA signal specifically affected?

We noticed that the amount of secreted proteins (normalized to the cell number) from *Vangl1/2* mutant cells was reduced compared to controls. This suggests a general defect in protein processing/secretion in the absence of *Vangl1/2*.

12) Figure 3J, 3L, need to show a field of cells before zooming in. Quantification is needed.

We have included images of a field of cells in Figure 3—figure supplement 4. Quantification of the percentage of high and low PDGFA-expressing cells is shown in Figure 3—figure supplement 4.

13) The finding that Vangl;Spc-^cre^ mutants showed simplification phenotype suggests that Vangl is required in AT2 cells. It does support that the phenotype is due to secretion defect, as concluded in subsection “Alveolar epithelial cells lacking VANGL2 fail to present the PDGF ligand to mesenchymal myofibroblasts”.

Indeed, the in vivo phenotypes in *Vangl1/2/Sftpc^CreER^* mutant lungs and the secretion defect in *Vangl1/2*-deficient cells support a model in which defective PDGFA secretion underlies the alveolar phenotype. However, we cannot rule out the possibility that other secreted ligands also contribute to the alveolar phenotype.

14) Figure 3K, only show histology, not "areas of defective alveolar development that was associated with loss of Vangl1/2". Antibody staining is needed to link mosaic inactivation to regional phenotype.

We have added this piece of data to Figure 3—figure supplement 5.

15) Subsection “Alveolar epithelial cells lacking VANGL2 fail to present the PDGF ligand to mesenchymal myofibroblasts”, unclear how the defect in cell contact is needed for PDGF secreted signaling.

There is evidence to suggest that cell-cell contact is required for signal transduction of several major signaling pathways. This could serve to deliver ligands directly to their receptors on the cell surface of responsive cells. Whether this is a universal phenomenon is unclear. In this regard, we do not have additional mechanistic insight into how loss of cell contact could affect PDGF signaling.

16) "Conversely, loss of PDGF ligand resulted in depletion of WNT5A-producing fibroblasts/myofibroblasts. These results support a positive feedback loop between WNT5A and PDGFA…" The staining shows in Pdgfa mutants the expression of PDGFRA expression. It does not show regulation of WNT5A. To demonstrate feedback, Wnt5a expression needs to be assayed in PDGF pathway mutants.

We have added qPCR analysis of *Wnt5a* in the revision; *Wnt5a* expression was reduced in *Pdgfa* knockout lungs (Figure 3—figure supplement 8). This further supports our model in which PDGF signaling preserves a pool of *Wnt5a*-expressing myofibroblasts.

17) Aside from in vitro migration assay, there is no other support for migration defect of Vangl mutants. Myofibroblast migration defects shown in Pdgfa mutant was found during sacculation stages. Is there an in vivo defect during sacculation of altered distribution of myofibroblasts?

We have added analysis of fibroblast/myofibroblast distribution in *Vangl2^f/f^; Pdgfra^Cre/+^* lungs at P0 and P5 (Figure 5D). We found no difference in the distribution of PDGFRA^+^ fibroblasts at P0. However, at P5 while PDGFRA^+^/SMA^+^ myofibroblasts had migrated to the prospective sites of secondary septa in control lungs, PDGFRA^+^/SMA^+^ cells in the mutant lungs stayed in the primary septa.

18) Figure 6A, it is unclear how AT1 that lack GFP, which presumably is not cre-recombined, show increase of p-Cofilin.

In fact, AT1 cells in the original Figure 6A had weak GFP expression. Nevertheless, to avoid confusion, we have replaced the original images with new ones that show stronger GFP expression in AT1 cells.

19) To really demonstrate AT1 wrapping of other cell types, thick section and 3D rendition is needed.

As suggested by the reviewer, we have used thick sections and 3D rendition to show AT1 wrapping of other cell types (Figure 6C, 6E).

20) In the smaller spheres, are there fewer cells, or smaller AT1 cells?

We placed the same number of cells onto each insert at the beginning of the experiment. The smaller spheres contain fewer cells derived from either control or *Vangl1/2*-deficient lungs. In control spheres, HOPX^+^ AT1 cells are located inside the spheres and are surrounded by SPC^+^ AT2 cells. This organization was not observed in spheres, in particular medium and small alveolospheres, derived from *Vangl1/2*-deficient cells. This is consistent with defective cellular properties in *Vangl1/2*-deficient AT1 cells required for morphogenesis.

21) For bleomycin experiment, het mutant control needs to be used. What is the phenotype on fibrosis?

Since het mutant (for *Vang2^f^)* and wild-type mice showed a similar response to bleomycin, we did not show both. Fibrosis occurred in control and *Vangl1/2*-deficient lungs and resolution of fibrosis took longer in the absence of *Vangl1/2*.

22) It is not clear why fibrosis was studied in mouse, but COPD was studied in patients.

Bleomycin-induced lung injury is traditionally used as a model of lung fibrosis. Perhaps, less publicized is the fact that this model also offers an opportunity to assess alveolar repair following bleomycin-induced alveolar loss. We have exploited this aspect of the bleomycin model in this manuscript and showed that loss of *Vangl1/2* compromises alveolar repair. In this context, our mouse studies have direct relevance to our findings that COPD patients displayed reduced levels of PCP pathway components. We have clarified these points in the revised manuscript.

Reviewer #2:

1) It is interesting that the Shh-Cre did not result in deletion of Vangl2, particularly given that the authors appear to show excellent recombination of the ROSA26-mTmG allele, and that is Cre is known to be strongly expressed in the lung epithelium. Do the authors have any explanation why?

We were also surprised that *Shh-Cre*, which is broadly expressed and widely used, was unable to effectively remove VANGL2 in the lung. The *ROSA26^mTmG^* allele is known to undergo recombination by even low levels of Cre activity; GFP was efficiently induced from the *ROSA26^mTmG^* allele by *Shh-Cre*. We observed the presence of *Vangl2^–^* (null; converted from *Vangl2^f^* by *Shh-Cre*) by PCR but only a few cells in the proximal airway had lost VANGL2 (Figure 1—figure supplement 4) and thus no phenotypes were observed. We do not know why *Sox9-Cre* was more effective than *Shh-Cre* in converting *Vangl2^f^* to *Vangl2^–^*. Perhaps, this is related to the relatively large distance between the two loxP sites in *Vangl2^f^* and a higher level of Cre expression is required. We also won't be surprised if *Shh-Cre* is unable to effectively convert other floxed alleles.

2) Although SOX9 is well known to be expressed in the distal lung epithelium during development, it is also expressed in the lung mesenchyme. Because the authors use the SOX9-Cre to characterize the effects of epithelial-specific loss of Vangl2, they should provide (ideally quantitative) data about the degree of recombination that the Sox9-Cre induces in the lung mesenchyme. One straight forward way to do this would be to add data using their Sox9-Cre;ROSA26-mTmG mice co-stained with a pan-epithelial marker such as Cdh1 or Nkx2-1. Significant recombination in the mesenchyme could significantly impact the interpretation of their data.

We have included data on analysis of *Sox9^Cre/+^; ROSA26^mTmG^/^+^* lungs in Figure 1—figure supplement 3. We found that GFP induced by *Sox9-Cre* was not found in the mesenchyme of the distal lung and was detected in few scattered mesenchymal cells in the large airway. The only place where a substantial number of mesenchymal cells expressed GFP was the trachea. This piece of information in conjunction with the alveolar phenotype induced by *Sftpc^CreER^* or *Aqp5^CreER^* support our model in which epithelial *Vangl1/2* plays a key role in controlling alveolar formation.

3) Why did the authors choose to use the Sox2-Cre when they were primarily interested in the distal epithelium (Figure 3E) – why not just stick with the Sox9-Cre (especially since these data are shown in the next panel, Figure 3F)

It turned out that *Sox9-Cre* was less efficient in inducing recombination of the *Pdgfa^ex4COIN^*allele than *Sox2-Cre*. In Figure 3E, we aimed to uncover the site and level of PDGFA expression in the lung and *Sox2-Cre* was more suitable for this purpose. As such, we were able to conclude that (1) PDGFA is expressed in both AT1 and AT2 cells and (2) not only more AT1 cells express PDGFA but AT1 cells also harbor higher levels of PDGFA than AT2 cells.

4) I don't understand the rationale for using whole-lung as the samples for RNA-Seq instead of FACS-sorted cells, especially when the authors had Sox9-Cre;ROSA26-mTmG;Vangl2 mice available, which would of easily permitted isolation of epithelial (and if desired, separate mesenchymal) cell population for RNA-Seq. The problem with a whole-lung bulk-seq analysis in this case, particularly when the authors have showed that epithelial loss of Vangl2 results in decreased proliferation in the mesenchyme is that the differential expressed gene list will be dramatically skewed by the alterations in cell types. I don't think the authors necessarily need to re-do these experiments for publication, but they should significantly limit the conclusions that they draw from these data. FACS-sorted cell populations or scRNA-Seq would have been preferred.

We agree with the reviewer about the pitfalls of using the whole lung as the source for RNA-Seq and have limited the conclusions that we can draw from these data. In the future, we will sort epithelial cells for RNA-Seq. Ideally, we also would like to sort myofibroblasts for RNA-Seq. However, this is complicated by the fact that it would be difficult to sort myofibroblasts away from airway smooth muscle cells and vascular smooth muscle cells since they express a similar set of markers.

5) In Figure 1—figure supplement 4, the alveoli appear somewhat compressed for P7-P30, particular P12-P30. Are these samples inflation-fixed? If not, this would be ideal, as this will impact the appearance of the alveolar structure, especially the relative thinness or thickness, as is trying to be illustrated. This may impact the authors’ conclusion about how the relative thinness/thickness changes throughout alveolar development.

We did not inflate the lungs when we fixed the tissues except for those used for EM studies. We have provided quantification of the relative thickness of the primary septa at different stages of development in Figure 1—figure supplement 5 (the old Figure 1—figure supplement 4).

6) The organoid data are somewhat difficult to interpret and doesn't seem to add much to the paper, but probably no harm in showing it either. While the organoids appear to be smaller, none of the organoid data provides any direct evidence why this would be. The fact that AT1 and AT proliferation appeared to be unaffected is probably the most useful observation. It may be worth just showing this directly in their knockout mouse model.

We agree with the reviewer that the organoid data added little insight into how *Vangl1/2* controls alveologenesis. The organoid system does not fully recapitulate alveologenesis in vivo. While AT1/2 proliferation does not seem to be affected in the organoids, AT1/2 proliferation was affected in mice. We should add that in control spheres, HOPX^+^ AT1 cells are located inside the spheres and are surrounded by SPC^+^ AT2 cells. This organization was not observed in spheres, in particular medium and small alveolospheres, derived from *Vangl1/2*-deficient cells. This is consistent with defective cellular properties in *Vangl1/2*-deficient AT1 cells required for morphogenesis.

7) Although the authors showed that AT1 and AT2 cell proliferation seemed to be unaffected following loss of VANGL1/VANGL2, it is probably a bit of a stretch (without additional data) that the reason for the impaired lung regeneration following bleomycin injury with Vangl1/Vangl2 deletion induced by SPC-^Cre-ERT2^ is an AT1 mediated defect.

We agree with the reviewer that additional studies are required to elucidate the molecular basis of impaired alveolar repair in the absence of *Vangl1/2*. We have modified our description to acknowledge this point.

Reviewer #3:

There are some problems:

1) The manuscript is unnecessarily extensive and diffused. The authors have piled up a vast amount of experimental designs some of which were just negative findings that lead them to the next set of studies, which turned out to be positive. The authors need to cull these extra data out of the manuscript and make it cogent as the alternative (the present form) reduces the impact of their findings.

We have reorganized the manuscript and culled the extra data as much as possible as suggested by the reviewer. We have to admit that this manuscript delivers so many new points that there is no easy way to present them without giving the impression that some are out of place.

2) The bulk of the genetic experiments used conventional knockout models. To be able to use the title that refers to alveologenesis, the authors had the option of using conditional (temporal) KO models, or they will have to show that the phenotypes they observe is not due to secondary causes of early development caused by the KO. This requires examining the KO mice in the embryonic stage.

Alternatively the authors may choose to change the title.

In the original submission, we showed that postnatal removal of *Vangl2* by *Sftpc^CreER^* induced alveolar phenotypes (Figure 3K). In the revised manuscript, we have added analysis of alveolar phenotypes induced by postnatal removal of *Vangl2* via *Aqp5^CreER^* (Figure 6I). Together, these studies on conditional knockouts further support the conclusions from conventional knockouts.

3) If no "No obvious difference in the gross morphology of the lungs was noticed between control and Vangl2^f/f^;Sox9^Cre/+^ mice prior to postnatal day 3" what was the reason for death on PN2?

In fact, there was no lethality at P2. Most of the *Vangl2^f/f^; Sox9^Cre/+^* mice died after P4/5. We have clarified this point in the revised manuscript. We should add that *Sox9^Cre^* exhibits extrapulmonary expression (Figure 1—figure supplement 3) and we cannot exclude the possibility that defects in other tissues may have contributed to the lethality.

4) The authors claim that Vangl1 plays a minor role in alveolar formation and loss of Vangl1 is compensated by Vangl2 in alveologenesis". As these were conventional KO mice, how can the authors know that Vangl1 has a role in alveolar formation?

We suggest that *Vangl1* plays a minor role in alveolar formation and loss of *Vangl1* is compensated by *Vangl2* in alveologenesis. This is based on the following observations: (1) *Vangl1^gt/gt^* mice have no apparent phenotypes; (2) *Vangl2^f/f^; Sox9^Cre/+^* mice display alveolar defects; (3) a fraction of *Vangl1^gt/+^; Vangl2^f/f^; Sox9^Cre/+^* mice exhibit more severe alveolar defects than *Vangl2^f/f^; Sox9^Cre/+^* mice; (4) no survivors beyond four weeks carry the genotype of *Vangl1^gt/+^; Vangl2^f/f^; Sox9^Cre/+^*. We also agree with the reviewer that a floxed allele of *Vangl1* is a better tool to define the role of *Vangl1* in alveologenesis. These points have been clarified in the revised manuscript.

5) It is interesting, as the authors state, that a small number of Vangl2^f/f^; Sox9^Cre/+^ mice survived beyond postnatal day 7." What was the histology of these lungs?

Histology of the survivors was shown in Figure 1—figure supplement 6 of the original submission (Figure 1—figure supplement 7 in the revised manuscript). Alveolar defects were observed in the survivors although not as severe as those in the non-survivors. In the revision, we also showed that IL13 expression was increased in the mutant lungs, suggesting altered immune responses.

6) In Vangl2^f/f^; Sox9^Cre/+^ embryos, is there a phenotype?

*Vangl2^f/f^; Sox9^Cre/+^* mutant lungs display no apparent defects in branching or sacculation. This information is now included in Figure 1—figure supplement 3.

7) In the studies using cells from control and Vangl1/2-deficient cells, what type of cells were isolated and how?

We have employed two types of cells for these studies, one from E13.5 mouse embryonic fibroblasts and another from E18.5 lung fibroblasts.

8) Figure 3E is supposed to show the level of PDGFA (LacZ). But, B-gal staining is not quantifiable. Nor is there an apparent significant difference.

The main purpose of Figure 3E was to show that PDGFA (LacZ) was expressed at a higher level in AT1 cells than AT2 cells, to which we have provided cell counting in the revision. Although β-gal staining is not indicative of the absolute level of PDGFA, staining on the same section does allow one to differentiate high expressers from low expressers.

Figure 3F showed that there is no apparent difference in PDGFA (LacZ) expression between control and *Vangl2^f/f^; Sox9^Cre/+^* lungs. We have added qPCR analysis of *Pdgfa* transcript levels in lungs of control and *Vangl2^f/f^; Sox9^Cre/+^* mice. There is no reduction (actually a slight increase) in *Pdgfa* mRNA levels in the mutant lungs.

9) In the bleo model in which Vangl1/2-deficient AT1 cells were compromised in their ability to form new alveoli, the authors should use a quantifying method such as MLI in areas of destroyed alveoli.

We have included measurement of MLI in areas of destroyed alveoli in control and *Vangl1/2/Sftpc^CreER^* lungs (Figure 6—figure supplement 1). As expected, the MLI was reduced in the mutant lungs.

10) The authors state: "We noticed that the primary septa in control lungs became visibly thinner during the first three days of postnatal life likely due to flattening of AT1 cells". Better and more convincing data would be required to include here.

We have provided quantification of the relative thickness of the primary septa at different stages of development in Figure 1—figure supplement 5 (the old Figure 1—figure supplement 4).

[Editors' note: further revisions were suggested prior to acceptance, as described below.]

We ask that you revise the text of your manuscript to more accurately describe your results. As reviewer 2 has noted, there are several unsubstantiated claims and unclear descriptions of some of your experiments. While we do not anticipate additional experiments are required to address these issues, please be advised that accuracy and rigor are important hallmarks of all eLife papers.

Reviewer #2:

1) Given the mortality timeline described in the manuscript, including some long-term survivors, in Sox9 ^Cre^/wt; Vangl2 ^f/f^ mice, the addition of a Kaplan-Meier survival curve would be more robust than a text description of their observations.

We do not have an accurate count of mortality at different postnatal stages. Once *Vangl2^f/f^; Sox9^Cre/+^* mice had shown signs of sickness. They were collected for phenotypic and molecular analysis. We suspect that these mice could have lived for a few more days. Thus, our methodology does not allow us to generate an accurate survival curve. By contrast, most survivors did not display signs of sickness and were collected at a later stage. We were able to conclude that the percentage of survivors is small. For instance, in our study that spanned several years, 7 mice survived beyond P21, in comparison with 68 *Vangl2^f/f^; Sox9^Cre/+^* mice collected at or prior to P7.

2) The authors state that the "initial expansion of fibroblasts/myofibroblasts was unaffected (Figure 5A)" (in Pdgfra ^Cre^/wt; Vangl2 ^f/f^ mice) and that "the primary role of mesenchymal PCP signaling is to control myofibroblast migration and function." In Figure 5A, while there appears to be no affect on proliferation of these cells at P3, there is a dramatic reduction in the number of EdU^+^ cells at P5.

Fibroblasts/myofibroblasts from *Vangl2^f/f^; Pdgfra^Cre/+^* lungs did not show proliferative defects at P3 when expansion of fibroblasts/myofibroblasts occurred. Therefore, we stated that the initial expansion of fibroblasts/myofibroblasts was unaffected. This finding also implies that epithelial *Vangl2* plays a key role in the initial expansion of fibroblasts/myofibroblasts. By contrast, myofibroblast migration was affected in *Vangl2^f/f^; Pdgfra^Cre/+^* lungs, suggesting that the primary role of mesenchymal *Vangl2* is to control myofibroblast migration and function. Reduced myofibroblast proliferation was observed in *Vangl2^f/f^; Pdgfra^Cre/+^* lungs at P5 when myofibroblast migration was active and pervasive. This observation suggests that either mesenchymal *Vangl2* controls subsequent myofibroblast proliferation or the migration defect exerts a secondary effect on myofibroblast proliferation or both. We have clarified these points in the revised manuscript.

3) The authors state that "abnormal myofibroblast development first appeared on day 2…" What exactly do they mean by this? If it’s decreased proliferation then this should be stated. If they mean something else, then the data to support it should be directly referenced.

We have revised our statement to indicate that “decreased myofibroblast proliferation first appeared on day 2…”

4) The authors state (in regards to the mesenchymal conditional knock-out of Porcupine), that "these results support the notion that non-canonical Wnt signaling in the lung mesenchyme initiate PCP signaling for alveolar development." Although the paper as a whole presents a variety of data that supports this concept, the manuscript contains a number of statements like this over-interpreting individual experiments. As the authors themselves state, porcupine is required for the release of all Wnt ligands, so this particular experiment does not differentiate between the roles of canonical and non-canonical Wnt signaling in alveolar development.

As suggested by the reviewer, we have removed “non-canonical” in the revised manuscript.

5) Similarly, from the same set of experiments the authors conclude that there is "no effect on differentiation of AECs." The only data presented to support this statement are two images from T1a/SPC double-stains of wild-type versus conditional KO lungs. At most, from these data you could say that there was no obvious difference in the number of type 1 and type 2 AECs, but even that statement would require counting cell numbers across a number of animals and sections.

We have changed the statement to “…differentiation of alveolar type I and type II cells” as suggested by the reviewer.

6) The inclusion of the SPC conditional knockout seems out of place. The rest of the manuscript focuses on type 1 AECs, so these data do not seem to add much to an already long manuscript.

We included the SPC conditional knockouts to support the involvement of type II AECs in alveolar formation and to respond to critiques from the other reviewers.

7) In the earlier portion of the manuscript, the authors focus on conditional knockouts of Vangl2 in various cellular compartments, but then for the intracellular trafficking studies, used cells derived from Vangl1^gt/gt^; Vangl2^-/-^ embryos. Why was the double-knockout studied instead cells deficient in Vangl2 only? Did the Vangl2 deficient cells not have the same phenotype?

Our genetic studies suggest that *Vangl1* plays a minor role in alveolar formation (due to the presence of *Vangl2*). To ensure a complete removal of signaling through *Vangl1/2*, we only derived cells from *Vangl1^gt/gt^; Vangl2^–/–^* embryos.

8) The images shown do not really seem to support the conclusion that PGFA-GFP is trapped within the ER. In Figure 3J, the PDGFA expression seems to be in a fairly uniform peri-nuclear distribution (not in the same punctate loci as the ER stain), and in the images in Figure 3—figure supplement 4 the PDGFA seems to just be uniformly distributed through the cytoplasm rather than "trapped in the ER."

The reviewer is correct that ER trapping occurred in a significant fraction of but not all of *Vangl1/2*-deficient cells. We have removed “trapped in the ER” in the revised manuscript.

9) Although I don't think there is anything wrong with the RNA-Seq data presented, I think is value to the manuscript continues to be significantly limited by the fact that it is derived from whole lung tissue in a knockout where the authors have already shown has dramatic alterations in the ratios of certain cell populations (e.g. depletion of myofibroblasts). In this situation, the conclusions that can be drawn a gene ontology analysis of these data are very limited (the GO categories will largely represent biological functions of cell populations that are enriched or depleted in the cKO relative to control). It really adds little to the data already presented in the paper, and doesn't really address the question that set out to answer with the experiment (e.g. "…to reveal the pathways controlled by epithelial PCP signaling.").

As suggested by the reviewer, we have deleted “To discover pathways that are affected by epithelial PCP signaling…” and also revised this section to indicate the limitation of RNA-Seq of whole lungs without cell sorting.

10) The manuscript could benefit from more clarity about what the authors think is happening in their injury model. Looking at the two images shown in Figure 6—figure supplement 1, the airspaces appear to be larger. However, the measured MLI for the conditional knockout is smaller. Do the authors think the alveoli are smaller after injury? If so, why? Also, their conclusions that "AT1 cells were compromised in their ability to form new alveoli" is a dramatic over-interpretation of these data, particularly given that the Sftpc-^CreER^ driver was used. While I agree there definitely appears to be an affect on injury repair, whether the defect is with type 1 or type 2 AECs or both, and what exactly the defect is remains entirely unclear.

We should emphasize that the two images in Figure 6—figure supplement 1A were derived from mice that received tamoxifen treatment alone without bleomycin. The enlarged airspaces in *Vangl1^gt/gt^; Vangl2^f/f^; Sftpc^CreER/+^*lungs were due to removal of *Vangl1/2*. The MLI measurement in Figure 6—figure supplement 1B was conducted on mice that received both tamoxifen and bleomycin. The reduced MLI in *Vangl1^gt/gt^; Vangl2^f/f^; Sftpc^CreER/+^*lungs indicates a reduced distance between two primary or secondary septa in the regenerating alveoli, consistent with the definition of smaller alveoli. We suspect that smaller alveoli are related to alterations in the cytoskeleton in the absence of *Vangl1/2* but this notion would require additional investigation.

We have revised our statement to indicate that the observed effect on injury repair could be due to AT1 or AT2 or both.

11) Again, the organoid data are intriguing but I'm not sure how much it really adds to the story. I suppose it supports the general concept that Vangl2 is required for general cellular organization, but again exactly what is happening is pretty difficult to really understand.

We agree with the reviewer that the organoid data supports the general notion that the *Wnt5a–Ror2–Vangl2* axis is required for cellular organization. However, the molecular mechanism by which *Wnt5a–Ror2–Vangl2* signaling confers cellular properties requires additional studies, which are beyond the scope of this manuscript.
